# Effect of midwifery students’ continuity of care program on women’s experiences of maternity care: A randomised controlled trial

**DOI:** 10.1371/journal.pone.0353118

**Published:** 2026-07-24

**Authors:** Elham Jafari, Mojgan Mirghafourvand, Shamsi Abbasalizadeh, Shahla Meedya, Leila Doshmangir, Sakineh Mohammad-Alizadeh-Charandabi

**Affiliations:** 1 Department of Midwifery, Faculty of Nursing and Midwifery, Zanjan University of Medical Sciences, Zanjan, Iran; 2 Social Determinants of Health Research Center, Tabriz University of Medical Sciences, Tabriz, Iran; 3 Department of Midwifery, Faculty of Nursing and Midwifery, Tabriz University of Medical Sciences, Tabriz, Iran; 4 Women’s Reproductive Health Research Center, Department of Obstetrics & Gynecology, School of Medicine, Tabriz University of Medical Sciences, Tabriz, Iran; 5 School of Nursing and Midwifery, Western Sydney University, Sydney, Australia; 6 Department of Health Policy and Management, Tabriz Health Services Management Research Center, School of Management and Medical Informatics, Tabriz University of Medical Sciences, Tabriz, Iran; College of Nursing, King Saud University, SAUDI ARABIA

## Abstract

**Background:**

Midwifery continuity of care (CoC) benefits women and families. Engaging midwifery students may help extend such care and improve access to quality maternity services. This study evaluated the impact of a student-led CoC on women’s maternity care experiences during childbirth in a middle-income country. No trials to date have assessed these effects.

**Methods:**

We conducted a randomized controlled trial with low-risk pregnant women at 26–29 weeks of gestation, intending vaginal delivery. Participants were randomized to intervention (n = 45) or control (n = 48) groups through stratified block randomization with central allocation concealment. The intervention group received continuous care from final-year midwifery students, while both groups received usual care. Childbirth experiences were assessed 40−50 days postpartum using the Childbirth Experience Questionnaire (CEQ-2), while secondary outcomes were measured with the Experience of Maternity Care and Maternal Satisfaction scales.

**Results:**

All recruited women were followed up, and baseline characteristics were comparable. In the intervention group, 12 women (27%) had caesarean sections compared to 18 women (38%) in the control group. Among women with experience of labour pain at the hospital (37 intervention, 35 control), the intervention group reported higher mean childbirth experience scores (3.3 vs. 2.3; adjusted mean difference [AMD] 1.03, 95% confidence interval [CI] 0.8 to 1.3). The intervention group scored higher in maternity care experiences during pregnancy (AMD 15.7; 95% CI 13.3 to 18.2), labour and birth (35.6 vs. 23.0; AMD 12.6, 95% CI 7.9 to 17.4), and postnatal care (45.1 vs. 28.2, AMD 17.1; 95% CI 14.0 to 20.2). Maternal satisfaction was greater for normal (187 vs. 130; AMD 56, 95% CI 45–68) and caesarean (162 vs. 130, AMD 30, 95% CI 11–50) births among women in the intervention group.

**Conclusions:**

Undergraduate midwifery students were able to provide CoC that improved women’s experiences of maternity care. These findings are promising for further research and practical implementation.

**Trial registration** Iranian Registry of Clinical Trials, IRCT20100414003706N41, registered prospectively on 30 April 2022, https://trialsearch.who.int/Trial2.aspx?TrialID=IRCT20100414003706N41; https://irct.behdasht.gov.ir/trial/62761.

## Introduction

In recent years, primary focus in maternal health has shifted from enhancing physical survival during childbirth to improving women’s experiences of maternity care and understanding its impact on the well-being of mothers and their families [1]. Key factors influencing women’s experiences of maternity care include organizational and interpersonal aspects of care, individual needs and expectations, as well as physical interventions throughout pregnancy, childbirth, and the postpartum period [2].

A systematic review identified four key themes associated with women’s childbirth experience: women’s perceptions, emotions, physical aspects, and relationships [1]. Negative experiences are often attributed to inadequate preparation, lack of control, abandonment, severe pain, being ignored, disrespectful care, poor communication, emergency caesarean, and prolonged labour [1,3]. A mixed-methods systematic review highlights current mistreatment during women’s childbirth—including physical and verbal abuse, lack of supportive care, neglect, discrimination, and denial of autonomy—occurs universally across all geographic and income-level settings [4].

To achieve the global priority of high-quality maternity care [5], it is essential to implement evidence-based guidelines tailored to women’s individual, cultural, personal, and medical needs [6]. Evidence-based clinical practice guidelines for maternity care recommend midwifery continuity of care (MCOC) due to its respectful, communication-focused approach [7]. The latest Cochrane review indicates that MCOC enhances positive maternity care experiences while simultaneously reducing antenatal and intrapartum costs. The review strongly advocates for the implementation and scaling up of MCOC in low- and middle-income countries (LMICs) [8]. However, access to MCOC remains limited for many women in LMICs [9], and existing reviews indicate limited evidence regarding the effectiveness of midwifery-led care in these settings [8–12].

In Iran, a middle-income country, there are significant concerns regarding maternity care experiences, the performance of midwives at the bedside, and the quality of midwifery education. The finding of qualitative studies conducted in Iran indicated that women often encountered physical and verbal abuse, deprivation of standard care, poor communication, lack of supportive care, neglect, abandonment, exclusion from clinical shared decision-making, and violations of privacy during childbirth [13–15]. Maternity care in Iran is not woman-centred, and midwifery-led models of care are scarce. Care is predominantly provided within a medical model, with most women birthing in hospitals where midwives work under direct obstetric supervision, often lacking professional independence [16,17].

In Iran, midwifery bachelor’s students are admitted through a national exam after high school and complete 130 theoretical and clinical courses over four years. Advanced studies are available at the master’s and doctoral levels [18]. However, midwifery students have limited opportunities to gain practical experience in midwifery-centered care during their course, as the National Curriculum does not mandate MCOC experience [18]. The over-medicalization of maternity care [19], along with negative experiences, such as the gap between theoretical knowledge and clinical practice, lack of instructor support, and feelings of worthlessness, lead to distress, frustration, and reduced self-esteem among midwifery students [20]. The connection between midwifery education and practice is inefficient, with curricula that lack programs to establish clinical preceptors, mentors, and role models [18].

Enhancing midwifery education is a strategic approach to improving the quality of midwifery care and expanding midwifery-led models in LMICs [9,21]. Effective midwifery training serves as a cost-effective tool that reduces the burden on healthcare systems, facilitates the implementation of midwifery-led care, and strengthens the midwifery profession [9,22].

The Midwifery Student Continuity of Care (MSCOC) program provides continuous, relationship-based, individualized, and woman-centred care by midwifery students throughout the pregnancy, childbirth, and postpartum periods. MSCOC is a mandatory component of pre-registration midwifery curricula in several high-income countries [23]. However, this model is not routinely implemented in LMICs, and available studies on the topic are limited. A recent review identified only one of 14 reports on MSCOC from LMICs [9].

Qualitative studies conducted in developed countries indicate that within the MSCOC model, women establish deep relationships with midwifery students, experience emotional security, and receive individualized care tailored to their needs. This model assists women in preparing for childbirth and enhances their ability to focus on the birthing process [24,25]. Additionally, MSCOC enhances students’ motivation to understand and adopt the MCOC model after graduation [26].

Recognizing the importance of women’s experiences with maternity care, the need to expand MCOC in LMICs, and the potential of midwifery education as a cost-effective strategy for promoting MCOC, this trial aimed to determine the effect of a midwifery students’ continuity of care program on women’s experiences of maternity care, with the childbirth experience as the primary outcome.

## Methods

This study is part of a larger mixed-methods project titled “Implementation and evaluation of a continuous care model by midwifery students during pregnancy, childbirth and postpartum: a mixed-methods study with an embedded experimental design”. This manuscript reports the quantitative experimental component of that project. Some results have also been reported in another paper that is currently under review. To avoid duplication, we have summarized portions of the methods section here. Readers are encouraged to refer to the aforementioned paper for detailed information. This study received approval from the Tabriz University of Medical Sciences in Tabriz, Iran (Ethical Code: IR.TBZMED.REC.1401.094).

### Study design

This study was a superiority randomised controlled trial (RCT) with two parallel arms. Due to the nature of the intervention, it was not feasible to blind the participants or care providers. Furthermore, blinding the outcome assessor was impractical, as the outcomes were self-reported by participants.

### Participants

The study included women with a singleton pregnancy between 26 and 29 weeks of gestation, at least six years of education, no more than two prior vaginal births, no previous caesarean sections, at least six years of education, and a willingness to have a vaginal birth at one of the nine city-level main hospitals. Exclusion criteria included medical or obstetric high-risk conditions, current pregnancy complications, major fetal abnormalities, recent major stressful events, lack of smartphone or internet access, or participation in another clinical trial.

Approximately two months after beginning participant recruitment, due to slow progress, we expanded the study setting from two teaching hospitals to all city-level hospitals with high delivery rates, following approval from the research council. This expansion added one social security hospital and six private or semi-private hospitals, while excluding one hospital that did not grant the necessary permit. To control for the effect of hospital type, we stratified randomization by hospital type (teaching, social security, and private/ semi-private).

Twenty-five final-year trained undergraduate midwifery students from Tabriz University of Medical Sciences provided care to women in the intervention group, under the supervision of an experienced mentor in coaching and conducting workshops on physiological childbirth—the principal investigator (PI), who is a PhD candidate (EJ).

### Recruitment

Women were recruited from public health centres in Tabriz, Iran. The PI identified eligible women through the online National Integrated Health System (IHS) called “SIB.” She contacted these women by phone, provided initial study information, and reviewed additional eligibility criteria. Potential participants were invited to the health center for an in-person meeting, where the PI provided detailed study information, reviewed eligibility criteria, obtained written informed consent, and asked the women to complete baseline questionnaires.

Efforts were made to ensure that each day during recruitment visits, at least four eligible pregnant women were present at the recruitment centre or at 2–3 nearby centres. 1–3 (usually two) midwifery students, together with the PI, were present during recruitment visits to enable initiation of the first intervention session immediately for participants allocated to the intervention group.

### Sample size

The sample size was calculated using a t-test for the difference between two independent means in G*Power software. Based on a previous study conducted in the same setting [27], with a mean childbirth experience score of 2.71 (SD1 = 0.73), an anticipated increase to 3.25 (a 20% improvement), SD2 = SD1, a two-sided α = 0.05, and 85% power, the required sample size was 34 per group. Considering a possible dropout rate of 30%, 46 participants were targeted for each group. As the primary outcome scale is not applicable to women who do not experience labour pain, a relatively high dropout rate was anticipated due to the likelihood of caesarean sections being performed before the onset of labour pain.

### Sequence generation and randomization

The allocation sequence for participating women was generated using a computer program with randomly varied block sizes of 4 and 6, a 1:1 ratio. This sequence was stratified by parity (nulliparous, multiparous) and intended hospital type for delivery (teaching, private/semiprivate, and social security-based hospital). A person who was not involved in recruitment, randomization, intervention, or data collection generated the sequence. Allocation was concealed centrally. Simple randomization assigned participants in the intervention group to midwifery students. Each student served as the primary care provider for two (in a few cases, one) pregnant, while also supporting two additional women assigned to another student. On the recruitment day, the PI introduced the participants in the intervention group to their respective primary student care providers and, if feasible, to their additional supporters.

### Intervention

Women in the intervention group received MSCOC. Before the recruitment of the women, the participating midwifery students received specific training that included four workshops. Each workshop lasted four hours, covering professional communication and clinical midwifery skill training, led by the mentor. A Telegram group was established to facilitate ongoing communication between the mentor and the students, serving as a platform for sharing educational content, reminders, and discussions.

Recruitment was conducted at 26–29 weeks of gestation to provide sufficient time to establish continuity relationships between women and midwifery students and to implement the intervention before labour, with continued follow-up throughout childbirth and the postpartum period.

The continuity of care provided by students included about nine antenatal care episodes (two in-person and several scheduled phone/video sessions), birth attendance, and four postnatal follow-ups up to six weeks postpartum. The intervention commenced immediately after recruitment and baseline assessment. During the initial in-person session, the students introduced themselves and explained the two-way communication process. They took a brief history, conducted assessments, and clarified the participants’ preferred communication method (video call or phone). This was followed by four phone/video counselling sessions spaced 7–10 days apart. A second in-person session was conducted at 35–36 weeks of gestation, followed by weekly phone/video counselling sessions from 37 weeks until delivery. Postpartum follow-up included one in-person hospital visit within 12–24 hours after childbirth and three phone/video counselling sessions at 3–5 days, 7–10 days, and 20–30 days postpartum.

Phone/video sessions were conducted using telephone calls or online communication platforms according to women’s preference and accessibility and were intended to provide counselling, education, emotional support, and continuity of communication.

Students provided their contact information to the women for non-emergency queries (8 a.m. to 11 p.m.) and offered 24-hour access for emergencies. The mentor attended the in-person sessions, assessed student skills, and provided feedback. Students recorded adherence to the protocol using a designed form submitted to the mentor.

In all the sessions, students were asked to emphasize on effective communication, risk assessment, and addressing women’s common concerns and questions. The main topics of the antenatal sessions were as follows: 1st session: exercises, breathing, and relaxation techniques during pregnancy; 2nd: stages of natural childbirth; 3rd: fear of childbirth, associated challenges, and management strategies; 4th: labour pain and pain relief methods; 5th: breathing and relaxation techniques during labour and birth; 6th: practical training in breathing and relaxation techniques; 7th: preparation for childbirth; 8th: neonatal care; 9th and beyond: review of topics based on individual needs. Postnatal sessions addressed the following: 1st session: breastfeeding and neonatal care counselling; 2nd: addressing postpartum blues; 3rd: breastfeeding continuation and postpartum depression; 4th: concluding care, acknowledging collaboration, sharing feedback, and ensuring postpartum support up to 6 weeks postpartum.

To ensure continuity of care during labour and birth, women were instructed to inform their primary student care provider when they began experiencing labour symptoms or upon hospital admission. During the active phase of labour, the student provided continuous bedside support, including emotional encouragement, guidance on breathing and relaxation techniques, assistance with position changes and massage, and attention to physiological needs until two hours post-delivery. The mentor, if feasible, was present in the labour ward to monitor and discreetly guide the student. Otherwise, the students worked under the supervision of a registered midwife who was the in-charge of the care. Deliveries were conducted by hospital midwives and obstetric staff according to hospital routine practice. In cases where a caesarean section was indicated during active labour or scheduled electively, to present in the operating room; however, this was occasionally prevented by operating room staff. If the student could not attend during labour and delivery due to late notifications, rapid vaginal birth, or emergency caesarean sections before the active phase of labour, the student would visit the woman in the hospital as soon as she was informed.

Both the intervention and control groups received routine antenatal, intrapartum, and postpartum care in accordance with national guidelines provided by licensed midwives and/or obstetricians. The MSCOC intervention was implemented as a supplementary continuity-of-care model by trained midwifery students and was not intended to replace the routine maternity care services. Additionally, participants in both groups were free to participate in childbirth preparation classes. They also had the option to be accompanied during labor by a private midwife or a female family member, depending on hospital policies.

All participants received routine prenatal and postpartum care at public health centers provided by midwives, with the option to consult private obstetricians or midwives. The routine care for low-risk pregnancies includes eight visits and three postpartum visits. Midwives providing these services are not involved in labor or childbirth care [28].

In Iran, public childbirth preparation classes are offered free of charge and voluntarily at selected hospitals and health centers for pregnant women. These classes, conducted over eight group sessions between the 20th and 37th weeks of pregnancy, cover a range of topics, including anatomical and physiological changes, personal hygiene, nutrition, fetal development, physical and psychological health, stages of natural childbirth, pain management, postpartum care, and neonatal care. Each session incorporates relaxation and breathing exercises, along with stretching techniques. The classes are primarily led by midwives who do not provide care during labor or the postpartum period [29]. However, participation in these classes is very limited.

In teaching hospitals, women receive labour and delivery care from midwives and obstetrics residents. The care is primarily medical, led by obstetrics residents, with midwives having a limited role in decision-making. Overmedicalization often restricts women’s involvement in decision-making, position choices, movement during labour, companion presence, and access to pharmacological and non-pharmacological pain relief. Despite official guidelines on respectful care, these are inconsistently followed, and midwifery-led care, including continuous care, is not routinely implemented [30]. Although private hospitals tend to provide more respectful care, the approach still follows a biomedical model and is not woman-centered [17].

### Outcomes

The primary outcome was the women’s childbirth experience which was assessed using the Childbirth Experience Questionnaire version 2.0 (CEQ-2) at 40–50 days postpartum. The CEQ-2 is typically administered between 4 and 8 weeks postpartum [31–33]. Secondary outcomes included experiences of maternity care during pregnancy, labour and birth, and early postnatal periods, as well as breastfeeding self-efficacy, which were assessed at 40–50 days postpartum (with experiences of maternity care during pregnancy also assessed at 35–36 weeks of gestation) via the Experience of Maternity Care and the Breastfeeding Self-Efficacy Scale. These outcomes, which may also be influenced by postnatal care, were assessed 20–30 days after the intervention ended. Furthermore, other secondary outcomes were maternal satisfaction with normal and caesarean births, support and control in birth, type of birth, and the duration of hospital admission until birth, assessed at 12–24 hours postpartum. The type of birth and length of hospital stays until birth were extracted from birth records. Postpartum depression and fear of childbirth were also assessed, which are the subject of a separate paper. The validated Persian versions of the following scales were utilized to evaluate the outcomes.

**The Childbirth Experience Questionnaire version 2.0 (CEQ-2)** includes 23 items categorized into four domains: own capacity, professional support, perceived safety, and participation. Twenty items are rated on a four-point Likert scale (scored 1–4), while three items utilize a visual analogue scale, which is then converted into values from one to four. Reverse scoring is applied to negatively worded items. Scores for the overall scale and subscales are calculated based on the mean scores of the respective items [31]. This scale has been validated for the Iranian population, showing a Cronbach’s alpha of 0.93 and an intraclass correlation coefficient (ICC) of 0.97 [34]. In our study, the Cronbach’s alpha coefficient was 0.96, with subscale values ranging from 0.74 to 0.91.

**The Experience of Maternity Care (EMC)** includes three scales assessing women’s experiences during pregnancy (EMC-PR, with five subscales), labour and birth (EMC-LB, with two subscales), and early postnatal care (EMC-PN, with three subscales). Each scale has 12 items rated on a Likert scale (scored 0–4), with some items reverse-scored. Scores for the scales and subscales were calculated by summing up the scores of the relevant items [35]. The validity of these scales has been confirmed among Iranian women, with Cronbach’s alpha values exceeding 0.9 and ICC above 0.8 [36]. In this study, the Cronbach’s alpha coefficients were 0.94 for EMC-PR, 0.93 for EMC-LB, and 0.95 for EMC-PN. The subscale values ranged from 0.74 to 0.92, except for the Antenatal Checks subscale, which had a value of 0.58.

**The Scale for Measuring Maternal Satisfaction in Normal and Caesarean Birth (SMMS-normal birth and SMMS-caesarean birth)** evaluates satisfaction with these birth types using two separate scales, each containing 42 Likert-scale items (scored 1–5), and 10 subscales. Scores are determined by summing the relevant item scores. Both scales have been validated for the Iranian population, with Cronbach’s alpha coefficients of 0.91 and ICC values exceeding 0.98 [37,38]. In our study, the Cronbach’s alpha coefficients were 0.98 for SMMS-normal birth and 0.95 for SMMS-caesarean birth. Subscale alpha coefficients ranged from 0.87 to 0.96 for SMMS-normal birth and from 0.73 to 0.90 for SMMS-caesarean birth.

**The Support and Control in Birth (SCIB)** has 31 items rated on a 5-point Likert scale (scored 1–5), including some reverse-scored items across three subscales. This scale has been validated for the Iranian population, with Cronbach’s alpha of 0.95 and an ICC of 0.99 [39]. In our study, the Cronbach’s alpha coefficient was 0.98, with subscale values ranging from 0.93 to 0.96.

**The Breastfeeding Self-Efficacy Scale (BSES)** consists of 33 items rated on a 5-point Likert scale (scored 1–5) [40]. This scale has been validated in Iran, with a Cronbach’s alpha of 0.82 [41]. In our study, the Cronbach’s alpha coefficient was 0.97.

### Statistical analysis

We utilized SPSS version 24 for data analysis. To ensure the accuracy of our data, we conducted a range check and performed an independent review of 10% of randomly selected cases. To mitigate loss to follow-up and address missing data, we reviewed the questionnaires immediately after completion and requested participants to provide any missing information. For the outcome related to EMC-PR, as there was no significant interaction effect of post-intervention time and group on this outcome, we used data collected 40–50 days postpartum to impute values for four individuals (two in each group) who were lost to follow-up at 35–36 weeks of pregnancy.

Our analyses followed a modified intention-to-treat approach, excluding participants who did not experience labour pain from CEQ-2, SCIB, and EMC-LB. Additionally, individuals who did not have a vaginal birth were also excluded from the analysis of the duration of admission until birth.

The quantitative data exhibited skewness of less than 2.5 and kurtosis of less than 4, indicating a normal distribution. Comparisons between the two groups were conducted using the analysis of covariance (ANCOVA) adjusted for parity and hospital type, except for EMC-PR, which was analysed using repeated measures ANOVA, and the delivery type, which was analysed using the chi-square test.

## Results

**Fig 1:**
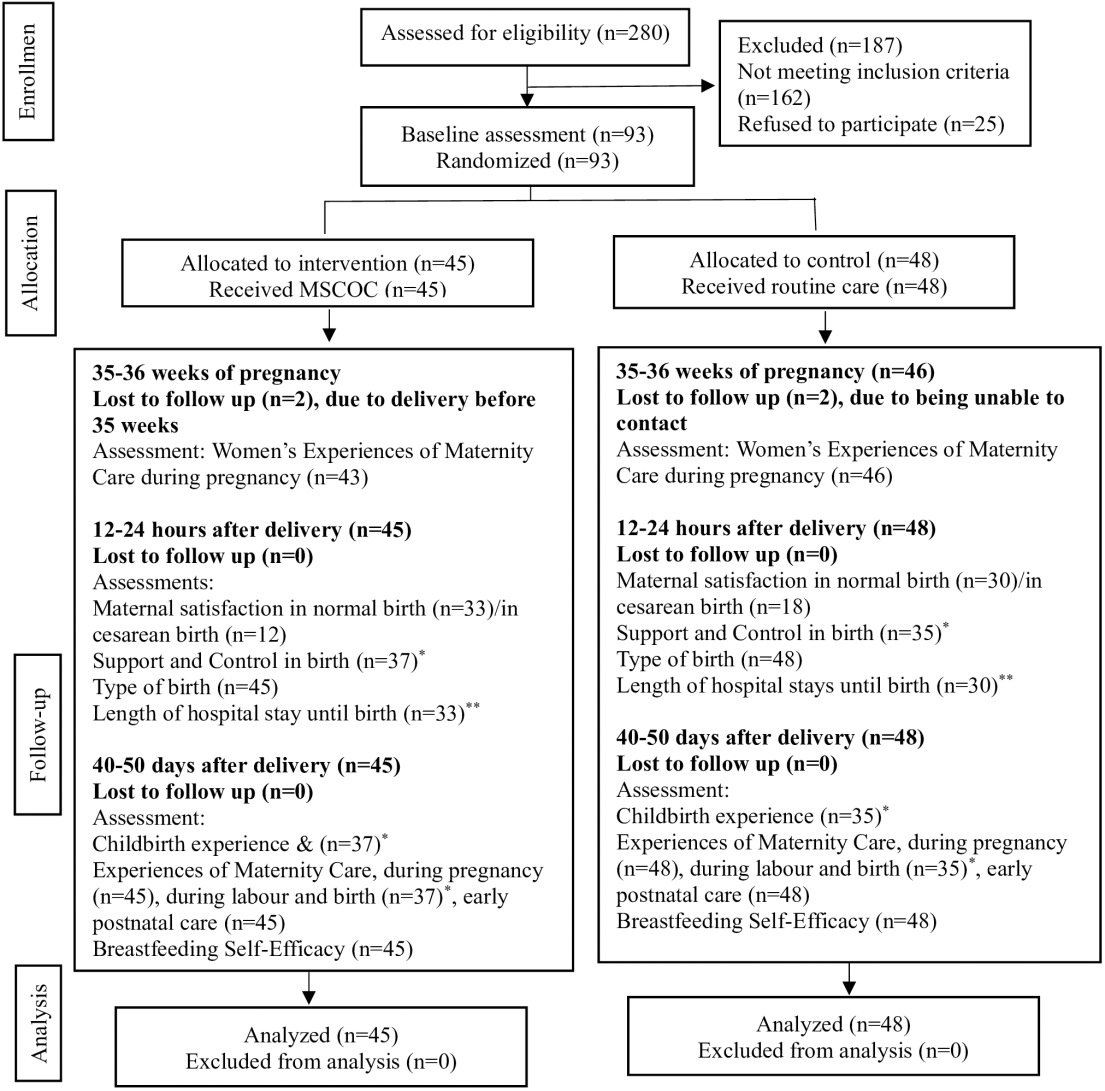
Flow diagram of the trial. MSCOC: Midwifery Student Continuity of Care. * Individuals who experienced labour pain. ^**^Individuals who had a normal birth.

Women were recruited from November 2022 to February 2023, with follow-up concluding in July 2023. Each of the 27 eligible public health centres in Tabriz provided one to six participants. Out of the 280 women assessed, 93 eligible women were randomly assigned to the MSCOC group (n = 45) or the control group (n = 48). Follow-up was completed 40–50 days postpartum for all participants. Data for EMC-PR were imputed for four individuals (two in each group) who missed the follow-up at weeks 35–36 of pregnancy (Fig 1).

In total, 8 women in the intervention group and 13 in the control group, who did not experience labour pain, were excluded from the analyses of CEQ-2, SCIB, and EMC-LB. The reasons for no experience of labour pain were elective caesarean sections (4 vs. 10, including breech presentations (1 vs. 1)), and emergency caesarean sections performed before the active phase of labour (4 vs. 3).

The mean age of the women was 26.9 years (SD 4.6). About half of them had either a diploma or a university degree and were primiparous. Most participants were unemployed, reported relatively sufficient family income, and had received primary antenatal care from obstetricians.

Between-group comparisons of women who experienced labour pain showed that the intervention and control groups were similar in the baseline characteristics (Table 1). Furthermore, when considering all randomized women, the two groups also exhibited similar baseline characteristics, as detailed in the paper under review.

**Table 1:** Characteristics of women who experienced labour pain at the hospital by study groups.

Characteristics	MSCOC (n = 37)	Control (n = 35)
**Baseline characteristics**		
Age (years)	27.2 [6.0]	26.8 [6.7]
Education (Diploma or college)	17 (45.9)	17 (48.6)
Job (Unemployed)	37 (100)	32 (91.4)
Adequacy of family income		
Relatively enough	31 (83.8)	28 (80.0)
Quite enough	2 (5.4)	4 (11.4)
History of infertility (Yes)	3 (8.1)	1 (2.9)
Planned pregnancy	27 (73.0)	21 (60.0)
Primiparous	15 (40.5)	14 (40.0)
Pregnancy interval from previous birth (years)*	7.5 [3.2]	6.3 [3.4]
Gestational age (weeks)	27.7 [1.3]	27.7 [1.2]
Sex of the fetus (Female)	19 (51.4)	16 (45.7)
Main antenatal care provider		
Obstetrician	22 (59.5)	24 (68.6)
Midwife	15 (40.5)	11 (31.4)
Satisfaction from previous childbirth*		
Very satisfied or satisfied	11 (50)	10 (47.6)
Neither satisfied nor dissatisfied	5 (22.7)	6 (28.6)
Dissatisfied or very dissatisfied	6 (27.3)	5 (23.8)
**Other characteristics**		
Attendance in childbirth preparation classes	2 (5.4)	5 (14.3)
Accompanied during labor		
Private midwives	2 (5.4)	4 (11.4)
Family member	9 (24.3)	8 (23)
Place of delivery		
Teaching hospital	16 (43.2)	15 (42.9)
Social Security hospital	7 (18.9)	7 (20)
Private hospital	14 (37.8)	13 (37.1)

MSCOC: Midwifery student continuity of care. The data are presented as numbers (%) or mean [SD]. * Among 22 participants in the MSCOC group and 21 in the control group who had previous births.

In the MSCOC group, 34 women (76%) attended at least six antenatal sessions, and 29 women (64%) participated in three or more postpartum sessions. Student care providers were present during labour and delivery in 21 cases (47%). Among women with labour pain experience, the corresponding figures were 26 (70%), 22 (60%), and 17 (46%), respectively. Except for one case, the primary student midwife who conducted the antenatal sessions during pregnancy and the postpartum period also attended the woman during labor and childbirth.

The mean total childbirth experience scores at 40–50 days postpartum were significantly higher in the intervention group (3.3 vs. 2.3, Adjusted Mean Difference [AMD] 1.03, 95% Confidence Interval [CI] 0.8 to 1.3, P < 0.001), with significantly higher scores across all subscales (P < 0.001) (Table 2).

**Table 2:** Comparison of childbirth experience scores between groups.

Variable	MSCOC n=37*	Control n=35*	AMD (95% CI)**
**Childbirth experience (1–4)**			
**Total**	3.3 (0.5)	2.3 (0.6)	1.0 (0.8 to 1.3)
Own capacity	3.2 (0.6)	2.2 (0.7)	1.0 (0.7 to 1.3)
Professional support	3.5 (0.1)	2.3 (0.1)	1.1 (0.8 to 1.4)
Perceived safety	3.3 (0.1)	2.3 (0.1)	1.0 (0.7 to 1.3)
Participation	3.5 (0.1)	2.4 (0.1)	1.1 (0.8 to 1.4)

MSCOC: Midwifery Students’ Continuity of Care

The data present mean (standard deviation) unless otherwise specified.

The Childbirth Experience Questionnaire-2 (CEQ-2) was employed to evaluate the childbirth experience, with higher scores indicating a more positive childbirth experience at 40–50 days postpartum.

* Individuals who experienced labour pain at the hospital, including four women in the intervention group and five women in the control group, who underwent cesarean section after experiencing labor pain for at least two hours at the hospital.

** The analysis of covariance (ANCOVA), adjusted for parity and hospital type, showed significant differences (P < 0.001) in all comparisons.

At baseline, there were no statistically significant differences between the intervention and control groups in the mean total EMC-PR score (32.8 vs. 33.2) or its subscales. The total EMC-PR score at 35–36 weeks of pregnancy (42.4 vs. 27.9) and 40–50 days postpartum (44.7 vs. 28.1) was significantly higher in the intervention group compared with the control group (AMD 15.3, 95% CI 12.7 to 17.8, P < 0.001). Additionally, post-intervention scores for all EMC-PR subscales were significantly higher in the intervention group (P < 0.001) (Table 3).

**Table 3:** Comparison of experiences of maternity care scores during pregnancy between groups.

Outcomes	Baseline		35–36 weeks of pregnancy		40–50 days postpartum		AMD (95% CI)* after intervention
	MSCOC n = 45	Control n = 48	MSCOC n = 45	Control n = 48	MSCOC n = 45	Control n = 48	
Pregnancy scale (0–48)	32.8 (8.0)	33.2 (9.4)	42.4 (7.7)	28.1 (10.4)	44.7 (6.3)	28.1 (9.3)	15.7 (13.3 to 18.2)
Antenatal checks (0–8)	6.1 (1.5)	6.1 (1.5)	7.1 (1.2)	5.6 (1.5)	7.4 (1.1)	5.2 (1.6)	1.9 (1.5 to 2.4)
Care appraisal (0–12)	8.4 (3.0)	8.5 (2.4)	10.6 (2.4)	6.6 (3.5)	11.2 (2.0)	6.7 (28)	4.2 (3.5 to 5.0)
Information giving (0–8)	4.4 (2.2)	4.9 (2.6)	6.5 (2.2)	3.9 (2.5)	7.1 (1.9)	3.7 (2.5)	3.0 (2.3 to 3.7)
Communication (0–12)	7.3 (3.1)	7.5 (3.0)	10.8 (2.3)	6.4 (3.5)	11.1 (2.1)	5.9 (3.2)	4.8 (4.0 to 5.7)
Continuity (0–8)	6.5 (1.4)	6.4 (1.7)	7.4 (1.2)	5.6 (1.6)	7.8 (0.6)	6.6 (1.5)	1.5 (1.1 to 1.8)

MSCOC: Midwifery Students’ Continuity of Care

The data are presented as mean (standard deviation) unless otherwise specified.

The Experiences of Maternity Care scale for pregnancy (EMC-PR) was used, with higher scores indicating a more positive experience.

* Repeated measures ANOVA was used, adjusted for baseline values after imputing missed values at 35–36 weeks of pregnancy with values recorded at 40–50 days postpartum. The P-values for all of the comparisons were < 0.001. The effects of post-intervention time and time × group were not statistically significant (P > 0.05) in any of the comparisons.

The intervention group had significantly higher mean total scores compared with the control group for the EMC-LB (35.6 vs. 23.0, AMD 47.7, 95% CI 38.0 to 57.4, P < 0.001) and the EMC-PN (45.1 vs. 28.2, AMD 17.1, 95% CI 14.0 to 20.2, P < 0.001). Scores for all subscales in both EMC-LB and EMC-PN were also significantly higher in the intervention group (P < 0.001) (Table 4).

**Table 4:** Comparison of experiences of maternity care scores during labour and birth and postnatal periods between groups.

Outcomes	MSCOC		Control		AMD (95% CI)**
	n	Mean (SD)	n	Mean (SD)	
**EMC-Labour and Birth**					
Total (0–48)*	37^*^	35.6 (10.2)	35^*^	23.0 (12.7)	12.6 (7.9 to 17.4)
Care quality (0–28)	37^*^	22.0 (5.4)	35^*^	16.3 (7.4)	5.7 (3.1 to 8.4)
Care needs (0–20)	37^*^	13.6 (5.4)	35^*^	6.7 (6.0)	6.9 (4.5 to 9.3)
**EMC-Postnatal**					
**Total (0–48)**	45	45.1 (6.5)	48	28.2 (9.8)	17.1 (14.0 to 20.2)
Adequacy of postnatal care (0–16)	45	15.2 (1.9)	48	11.8 (4.2)	3.5 (2.2 to 4.7)
Health professionals’ communication (0–16)	45	14.9 (2.6)	48	8.1 (3.5)	6.9 (5.7 to 8.1)
Individualized care (0–16)	45	15.0 (2.3)	48	8.3 (3.2)	6.7 (5.6 to 7.8)

MSCOC: Midwifery Students’ Continuity of Care

The data are presented as means (standard deviations) unless otherwise specified.

The scales of Experiences of Maternity Care for labour and birth (EMC-LB) and postnatal care (EMC-PN) were used at 40–50 days postpartum, with higher scores indicating a more positive experience.

* Individuals who experienced labour pain.

** The analysis of covariance (ANCOVA), adjusted for parity and hospital type, showed significant differences (P < 0.001) in all comparisons

Significant differences were observed between the intervention and control groups in maternal satisfaction scores at 12–24 hours postpartum for both normal birth (187 vs. 130, AMD 56, 95% CI 45–68, P < 0.001) and caesarean sections (162 vs. 130, AMD 30, 95% CI 11–50, P = 0.004). In the case of normal births, the intervention group also scored significantly higher across all subscales of maternal satisfaction (P < 0.001). However, in caesarean sections, no significant differences were found between the intervention and control groups in the subscales about preparation for caesarean, hospital room, hospital facilities, and meeting expectations (Table 5).

**Table 5:** Comparison of maternal satisfaction and birth support/control scores between groups.

Outcomes	MSCOC	Control		AMD (95% CI)**	P-value
**SMMS-normal birth**	**n = 33**	**n = 30**			
Total (42–210)	186.7 (21.3)	129.6 (29.9)	56.4 (45.0 to 67.9)		<0.001
Perception of health professionals (4–20)	18.3 (2.8)	14.7 (3.7)	3.5 (2.0 to 5.0)		<0.001
Midwifery care in labour (2–10)	9.3 (1.7)	6.6 (2.6)	2.8 (1.7 to 3.9)		<0.001
Comforting (4–20)	16.5 (3.7)	8.6 (2.3)	7.9 (6.3 to 9.4)		<0.001
Information and involvement in decision making (8–40)	35.2 (4.7)	22.6 (6.0)	12.5 (9.9 to 15.1)		
Meeting baby (3–15)	14.7 (0.9)	10.7 (3.8)	4.0 (2.6 to 5.3)		<0.001
Postpartum care (6–30)	28.8 (1.9)	21.1 (5.1)	7.7 (5.8 to 9.5)		<0.001
Hospital room (4–20)	18.7 (2.6)	14.2 (4.5)	4.4 (2.8 to 6.0)		<0.001
Hospital facilities (3–15)	12.4 (2.6)	9.2 (3.1)	3.1 (2.1 to 4.2)		<0.001
Respect for privacy (3–15)	12.7 (3.0)	9.0 (3.4)	3.6 (2.4 to 4.9)		<0.001
Meeting expectations (5–25)	20.1 (5.5)	13.0 (5.1)	7.1 (4.5 to 9.7)		<0.001
**SMMS-caesarean birth**	**n = 12**	**n = 18**			
Total (42–210)	162.1 (28.5)	129.8 (25)	30.4 (10.8 to 50.0)		0.004
perception of health professionals (5–25)	21.5 (3.7)	20.3 (2.7)	0.9 (−1.5 to 3.3)		0.44
preparation for caesarean (2–10)	8.2 (2.3)	7.6 (1.4)	0.3 (−1.2 to 1.8)		0.70
Comforting (3–15)	11.1 (3.9)	6.5 (3.3)	4.1 (1.2 to 7.1)		0.007
Information and involvement in decision making (8–40)	28.6 (6.7)	20.1 (7.2)	7.9 (2.6 to 13.1)		0.005
Meeting baby (3–15)	11.4 (4.0)	6.2 (3.4)	5.0 (2.0 to 8.1)		0.002
Postpartum care (6–30)	25.3 (3.7)	21.8 (4.0)	3.5 (0.5 to 6.5)		0.03
Hospital room (3–15)	12.3 (3.2)	10.8 (3.0)	1.7 (−0.6 to 4.0)		0.14
Hospital facilities (3–15)	11.3 (2.7)	9.9 (3.5)	1.8 (−0.5 to 4.0)		0.11
Respect for privacy (4–20)	17.4 (1.8)	12 (3.7)	5.5 (3.2 to 7.7)		<0.001
Meeting expectations (5–25)	14.9 (4.1)	14.5 (4.6)	−0.2 (−3.9 to 3.4)		0.89
**Support and control in birth (SCIB)**	**n=37** ^*^	**n=35** ^*^			
Total (1–165)	133.9 (22.8)	85.04 (23.4)	47.7 (38.0 to 57.4)		<0.001
Internal control (11–55)	44.7 (9.5)	25.3 (8.7)	19.4 (15.3 to 23.5)		< 0.001
External control (6–30)	24.8 (6.0)	16.6 (6.0)	8.2 (5.6 to 10.7)		< 0.001
Support (14–70)	63.6 (9.3)	43.5 (11.5)	20.1 (15.4 to 24.8)		< 0.001

MSCOC: Midwifery Students’ Continuity of Care

The data are presented as mean (standard deviation) unless otherwise specified.

The Scales for Measuring Maternal Satisfaction in Normal Birth (SMMS-normal birth) and Caesarean Birth (SMMS-caesarean birth) were used to assess maternal satisfaction, while the Support and Control in Birth (SCIB) scale evaluated support and control. Higher scores on both scales indicated a better experience within 12–24 hours postpartum.

* Individuals in two groups who experienced labour pain.

** The analysis of covariance (ANCOVA), adjusted for parity and hospital type was utilized.

The mean total support and control score at 12–24 hours postpartum was significantly higher in the intervention group (134 vs. 85, AMD 48, 95% CI 38–57, P < 0.001), with significantly higher scores across all its subscales (P < 0.001) (Table 5).

In the intervention group, 33 women (73.3%) had a normal vaginal birth, 8 (17.8%) underwent emergency caesarean section, and 4 (8.9%) had elective caesarean section, one due to breech presentation. In the control group, the corresponding numbers were 30 (62.5%), 5 (10.4%), and 13 (27.1%), with two elective cases due to breech presentation. Overall, there was no statistically significant difference between the two groups in the mode of delivery (P = 0.06).

Among women who chose elective cesarean sections, two in the MSCOC group did so with their obstetrician’s encouragement, while one chose it at her own request. In the control group, four women underwent the procedure with the obstetrician’s encouragement, one was motivated by her mother-in-law, and six at their own request.

Among those who had normal vaginal births, the mean time from hospital admission to birth was significantly shorter in the intervention group compared with the control group (4.7 vs. 10.3 hours, mean difference: −5.6, 95% CI −7.6 to −3.6, P < 0.001). The shortest and longest durations in the MCOC group were 30 minutes and 15 hours**,** respectively, whereas in the control group they ranged from 1 to 23 hours. At 40–50 days postpartum, the mean score for breastfeeding self-efficacy in the intervention group (23.7 [SD 4.2]) was significantly higher than that in the control group (21.9 [SD 4.3]) (mean difference 1.8, 95% CI 0.5 to 3.1, P = 0.006).

## Discussion

We implemented the MSCOC model in Iran, a middle-income country, where there is a paucity woman-centred care and the maternity care system is predominantly medicalized. At 40–50 days postpartum, women in the MSCOC group reported significantly more positive experiences related to childbirth, maternity care, and breastfeeding self-efficacy compared with the control group. Additionally, at 12–24 hours postpartum, satisfaction with both normal and caesarean deliveries, as well as perceptions of support and a sense of control, was notably higher in the MSCOC group. Moreover, the time from hospital admission to birth was significantly shorter among women in the MSCOC group. While the rate of normal births was higher in the intervention group, this difference did not achieve statistical significance.

The findings of this study, which indicate a more positive childbirth experience among women in the MSCOC group, are consistent with the results from an RCT in Australia [42] and a cohort study in Sweden [43], both of which demonstrated the positive effect of MCOC on childbirth experiences. Possible key factors contributing to this positive experience include having a known midwife, receiving sufficient information, and experiencing strong support and respectful interaction [3,44]. Final year midwifery students appear to possess the potential to deliver MCOC effectively. Most studies on MSCOC conducted around the world are qualitative or descriptive [23], highlighting relational continuity as a prominent feature. This continuity, which is built on trust, confidence, interdependence, and collaboration, plays a critical role in ensuring a satisfying childbirth experience [10,24]. Women particularly value the individualized, woman-centred care provided by MSCOC, which fosters feelings of worth, acceptance, and empathy, ultimately enhancing their confidence. They also appreciate the close, equal, yet professional relationships that make them more receptive to advice and see their student midwife as an advocate in the delivery room, ensuring that their needs and preferences are effectively communicated [10,24].

Women in the MSCOC group reported significantly more positive perceptions of control and support during labour and birth, consistent with findings from trials in Australia [42] and Turkey [45]. In an Australian RCT, although pain intensity was similar between the MCOC and control groups, women in the MCOC group reported better control and a more positive perception of pain [42]. Similarly, the Turkish RCT found that intrapartum continuity of care by midwifery students positively influenced women’s perceptions of support and control during labour [45]. The higher internal and external control scores in the MSCOC group in the current trial likely stem from extensive educational and counselling sessions provided during pregnancy by the students. These sessions prepare women for labour, making them more responsible and realistic in their expectations, thereby improving their perception of control. Empowering women enhances their readiness and inner strength for childbirth [10]. Having a familiar companion during childbirth can significantly improve women’s confidence and sense of control by providing emotional support and a safe psychological environment [46]. As noted by McLachlan et al. (2016), with midwife support, women feel more empowered, increase their coping capacity, experience greater control, and gain confidence in their own abilities [42].

The results of this study indicate that women in the MSCOC group experience more positive maternity care during pregnancy, childbirth, and postpartum, as well as greater satisfaction with both normal and caesarean deliveries, which are consistent with findings from a pilot study conducted in Norway and an RCT in Australia. These studies demonstrated that MSCOC and MCOC enhance women’s satisfaction with maternity care at all stages [47,48]. Australian cohort studies have also reported that over 70% of women found MSCOC exceeded their expectations. Satisfaction and respect were positively correlated with more frequent antenatal and postnatal contact and the presence of a student midwife during labour and birth. The frequent meetings in the MSCOC model likely foster the development of rich and meaningful relationships [25,49]. Another Australian study found that most women expressed satisfaction with MSCOC, describing it as beneficial, high-quality, evidence-based care that effectively addressed their individual needs. Women appreciated their familiarity with their care provider, eliminating the need to repeatedly present themselves at each visit, and the provider’s clinical expertise [50]. According to a mixed-methods study, while providing specialist information and physical support contributes to women’s satisfaction, key indicators include continuity of care, the quality of communication, and essential communication skills such as empathy, patience, and understanding [51].

Notably, our finding concerning no significant difference between groups in subscales of satisfaction related to physical preparedness for surgery, hospital room, and facilities for caesarean aligns with a Swedish RCT that found that altering labour room conditions did not significantly improve childbirth experiences [52]. These results underscore the greater importance of interpersonal communication over physical factors for childbirth satisfaction.

Our finding concerning the shorter duration from hospital admission to birth in the MSCOC group compared with the control group is in line with other studies where midwifery students provided continuous care [45,53,54]. In our study, women in the intervention group notified their midwifery students when labour symptoms began. They were encouraged to stay at home, utilizing online or phone support from the student midwife (under the supervision of the mentor), until active labour commenced, provided no concerning symptoms were present. This approach likely explains our findings, as women in early labour typically prefer to stay in a familiar and calm environment [55]. Early labour often lacks sufficient support, and a sense of confusion during this phase can negatively impact the overall childbirth experience [56]. A meta-analysis supports our findings, showing that continuous midwifery care empowers women with information on the optimal time to go to the hospital, leading to fewer labour interventions and greater satisfaction [57]. In our study setting, where childbirth overmedicalization is common, reducing the hospital stay until birth in the intervention group may have improved childbirth satisfaction by minimizing unnecessary interventions.

Additionally, as supported by a Cochrane review, continuous labour support can speed up the physiological progression of labour and shorten its duration [58]. This finding also aligns with the modern view of childbirth as a neuro-psycho-social event, where a woman’s experiences are closely connected to neurohormonal processes. Based on this perspective, relaxation, support, and gentle sensory stimulation (e.g., touch) can boost oxytocin release, while reducing fear, stress, and pain, thereby accelerating labour. The integrative neuro-psycho-social model of childbirth emphasizes the critical role of midwifery-centred care in optimizing hormonal function, improving labour outcomes, supporting breastfeeding, and aiding maternal adaptation [59].

In this study, breastfeeding self-efficacy was also significantly higher in the MSCOC group compared with the control group. A study conducted in Greece similarly found that long-term midwifery support is associated with higher rates of exclusive breastfeeding and longer breastfeeding duration [60].

The lack of a statistically significant difference in delivery type between the MSCOC group and the control group, despite a higher rate of normal births in the MSCOC group (73% vs. 62%), is consistent with previous research [47,54,61]. However, a Cochrane review suggests that continuous support may increase the likelihood of spontaneous vaginal births [58]. In our study, the mode of delivery was a secondary outcome, and the sample size did not have sufficient power to detect a clinically significant effect. The caesarean section rate in Iran, at 52%, greatly exceeds WHO standards, likely due to the prevailing biomedical model of maternity care [61]. A high proportion of cesarean sections in Iran occurs for non-medical reasons, driven by socioeconomic factors, cultural norms, limited maternal awareness, misconceptions, and weak policy enforcement [62]. The Iranian healthcare system lacks an effective referral system [63]. A cross-sectional study found that over 82% of low-risk pregnant women receive prenatal care from obstetricians, a practice associated with a 2.3-fold increase in the odds of cesarean [64]. Financial incentives encourage obstetricians to perform non-medical cesareans. Most women prefer cesarean delivery due to reasons such as fear of labor pain and natural delivery, along with financial and insurance support for private care [61]. A qualitative study suggests that expanding midwifery continuity of care, strengthening midwifery autonomy, improving the quality of childbirth preparation classes, establishing an integrated care system for low-risk pregnancies, and providing insurance support for midwifery services could promote natural births and reduce cesarean rates [65].

### Strengths and limitations of the study

In this trial, strict adherence to random sequence generation and central allocation concealment effectively minimized selection bias. While blinding was not feasible, we mitigated performance and detection biases by withholding study outcomes from both participants and care providers. The intervention’s effects on some objective outcomes, such as reducing the duration of hospital admission to birth and increasing the rate of normal birth, though not statistically significant, lend further support to the validity of the study results. The few lost to follow-up and the absence of missing data in the completed questionnaires minimized the risk of attrition bias. We have reported the results of all outcomes to minimize the risk of reporting bias. Adherence to the study protocol, especially during antenatal and postpartum periods, was high, likely enhanced by the active engagement through a Telegram channel. Including participants from all eligible urban health centres in Tabriz, a city with average reproductive indicators of the urban areas in the country, strengthens the generalizability of the study, potentially making the findings applicable to urban settings across Iran.

In this study, the intervention was initiated in late pregnancy. Earlier implementation may have increased women’s exposure to continuity of care and potentially strengthened the observed intervention effects. We focused on low-risk women with at least six years of education who received prenatal care at public health centres, and the intervention occurred in an over-medicalised and non-woman-centred environment. These criteria may limit the applicability of our study to other populations; i.e., high-risk patients, illiterate women, those from higher socioeconomic backgrounds attending private facilities, or contexts that prioritise woman-centred care. The relatively low rate of student attendance at the bedside during childbirth may have underestimated the effects. Additionally, the voluntary participation of students, supported only by the research team without strong institutional backing, may have influenced their motivation and adherence. Logistical challenges related to student travel and attendance should also be considered.

### Implications for practice

Despite these limitations, the study suggests that midwifery students can effectively demonstrate the benefits of MCOC for women’s childbirth and maternity care experiences. Therefore, opportunities to integrate the continuity of care models in midwifery students should be maximized, potentially incorporating these activities into midwifery curricula. Future research should investigate the optimal level of continuity required to achieve the desired outcomes, compare the effects of various mixed virtual and in-person sessions, and explore strategies to enhance student attendance during childbirth. Additionally, including women from the first trimester and studying high-risk populations could provide more comprehensive insight. The mentorship of all students by a single highly motivated person may have affected the results; thus, pragmatic trials involving mentorship by midwifery instructors could provide more applicable outcomes.

## Conclusions

The findings of this study demonstrated that MSCOC positively enhances women’s experiences of maternity care with a positive childbirth experiences. Therefore, introducing MSCOC into pre-registration midwifery curricula in LMICs appears valuable for improving maternal health and may also enhance students’ motivation to understand and implement the MCOC model after graduation. However, further research—especially pragmatic trials in various settings—is needed before implementation and scaling up of the model.

## Supporting information

S1 Appendix Dataset containing all data underlying the findings described in this manuscript. (XLSX)

S2 Appendix Protocol for an Implementation and evaluation of a continuous care model by midwifery students during pregnancy, childbirth and postpartum: a mixed-methods design with an Embedded Experimental Model. English version.
(PDF)

S3 Appendix Protocol for an Implementation and evaluation of a continuous care model by midwifery students during pregnancy, childbirth and postpartum: a mixed-methods design with an Embedded Experimental Model. Persian version.
(PDF)

S4 Appendix Socio-demographic and obstetric, childbirth, and neonatal characteristics questionnaire. (DOCX)

S5 Appendix CONSORT 2010 checklist of information. (DOC)

## Abbreviations

AMD: Adjusted mean difference
CEQ-2: Childbirth Experiences Questionnaire version 2.0
EMC-LB: Women’s Experiences of Maternity Care during Labour and Birth
EMC-PN: EMC during Early Postnatal
EMC-PR: EMC during Pregnancy
ICC: Intraclass correlation coefficient
LMICs: Low- and middle-income countries
MCOC: Midwifery continuity of care
MSCOC: Midwifery student continuity of care
PI: Principal investigator
RCT: Randomised controlled trial
SMMS: Scale for Measuring Maternal Satisfaction.
