# Supplementary material for: Effect of midwifery students’ continuity of care program on women’s experiences of maternity care: A randomised controlled trial
Source: PLoS One. 2026 Jul 24;21(7):e0353118. doi: 10.1371/journal.pone.0353118 (PMC13399285; doi:10.1371/journal.pone.0353118)
Supplement: S2 Appendix — English version. (PDF) [file pone.0353118.s002.pdf]

Implementation and evaluation of continuous care model by midwifery students during pregnancy, childbirth and postpartum: a mixed-methods design with Embedded Experimental Model

Elham Jafari [1], Sakineh Mohammad-Alizadeh-Charandabi [2*], Mojgan Mirghafourvand [2], Leila Doshmangir [3], Shamsi Abbasalizadeh [4], Shahla Meedya [5]

1. PhD Student in Midwifery, Department of Midwifery, Faculty of Nursing and Midwifery, Tabriz University of Medical Sciences, Tabriz, Iran
2. Social Determinants of Health Research Center, Tabriz University of Medical Sciences, Tabriz, Iran.
3. Department of Health Policy & Management, Tabriz Health Services Management Research Center, School of Management and Medical Informatics, Tabriz University of Medical Sciences, Tabriz, Iran.
4. Women's Reproductive Health Research Center, Department of Obstetrics & Gynecology, School of Medicine, Tabriz University of Medical Sciences, Tabriz, Iran
5. School of Nursing and Midwifery, Faculty of Health Sciences, Australian Catholic University, Australia.

ABSTRACT

Introduction: Continuity of care is an optimal goal in maternity care systems. Qualitative and observational studies have indicated the positive effects of continuity of care by midwifery students. In the Iranian educational system, midwifery students do not have any practical training in this type of care. In this study, we will introduce the continuity of care in midwifery training at one university and assess the effect of the model on women's childbirth experience, fear of childbirth, and postpartum depression. The qualitative study will be used to explore women's and midwifery students' experiences of the care.

Methods and analysis: This is a mixed study with an embedded experimental model. The quantitative phase will be a randomized superiority trial where 92 low-risk women at 26-29 weeks of gestation will be allocated into intervention or control groups using blocked randomization (stratified by parity) with central allocation concealment. The intervention group will receive continuity of care provided by the last-year undergraduate midwifery students. The outcomes will be assessed 40-50 days postpartum. ANCOVA will be used to compare the means between the groups. Data for the phenomenological qualitative phase will be collected via semi-structured individual in-depth interviews and analyzed by thematic analysis.

Ethics and dissemination: We obtained ethical approval from the ethics committee of Tabriz University of Medical Sciences. All research results will be published in international journals. A summary of the results will also be available to stakeholders in the form of approve knowledge translation.

Trial registration: Iranian Registry of Clinical trials, IRCT20100414003706N41, registered prospectively 30 April 2022, https://trialsearch.who.int/Trial2.aspx?TrialID=IRCT20100414003706N41; https://irct.behdasht.gov.ir/trial/62761.
.

Keywords: Midwifery, Students, Continuity of patient care, Pregnancy, Birth, Women.

1. Introduction

Designing and implementing effective maternal care models is a major goal of global health policies to promote maternal and neonatal health (1). Continuity of midwifery care is relationship-based care with a known midwife/midwives in which care is provided in a pre-arranged schedule as well as on-call throughout pregnancy and childbirth up to six weeks postpartum (2). According to a Cochrane systematic review, this model of care reduces the probability of preterm birth, fetal loss, neonatal death, instrumental vaginal birth, regional anesthesia, and episiotomy, and increases spontaneous vaginal birth (3). There is also evidence for its cost-effectiveness for low-risk women (4).

Despite the global attention to the key role of midwives and the emphasis on investing in this profession, using the capacities of this profession in Iran has been overlooked to some extent (5). Midwifery in Iran is defined as an independent profession, yet no professional mechanism has been provided for its independence. Prenatal care at public health centers is not woman-centered. Midwives who provide prenatal care follow a medical approach that includes several mandatory visits by a physician of the center, and there is no phone consultation (3). Also, the midwives working in the health centers do not involve in the birth process (6). In most urban areas, there is no positive attitude toward publicly available maternity services, and pregnant women prefer to receive services from the private sector (7).

Considering that the quality of midwifery training is vital for future woman-centered maternity care (8), the inclusion of continuity of care (COC) models into midwifery training seems pivotal (9). However, Iranian midwifery education lacks woman-centered and COC models. In some countries including Australia, the Netherlands, the United Kingdom, Canada, and New Zealand, COC experience is mandatory for midwifery students. It aims to enable the students to gain experience in providing COC, to understand women's experience of childbirth, and to get students ready for work in midwifery-led COC models (10).

Studies show a positive effect of the COC models on students' learning by positive influence on their confidence, preparation, and motivation for beginning practice (11, 12). Also, observational and qualitative studies indicated potential positive effects of COC by midwifery students on maternal and fetal outcomes. In these studies, most of the women receiving this type of care expressed a higher level of satisfaction (13) and valued their deep, meaningful, and effective relationship with students (14, 15) (Dahlberg & Aune, 2013; Kelly et al., 2014). The COC by midwifery students also associated with a reduction of smoking after 20 weeks of gestation (16), and a reduction of some adverse outcomes including prolonged labor, asphyxia (17), cesarean delivery, third- and fourth-degree tears, and episiotomy (16).

To our knowledge, there is no interventional study in the world, and any type of study in Iran to determine the effectiveness of the COC model by midwifery students. Therefore, this mixed-method study was

designed for the implementation and evaluation of COC by midwifery students during pregnancy, childbirth, and postpartum. The primary objectives are as follows:

1. The quantitative phase: to determine the effect of implementing the COC model by midwifery students on the childbirth experience, fear of childbirth, and postpartum depression.
2. The qualitative phase: to explore women's viewpoints regarding the COC model by midwifery students.
3. The combined purpose: to provide a deeper understanding of different aspects of the COC model implementation.

2. Methods and analysis

2.1. Study design

This is a mixed study with the embedded experimental model. The quantitative stage of the study is a superiority randomized controlled trial with two parallel arms to assess the effectiveness of the COC by midwifery students. The qualitative stage with a phenomenological approach will be carried out to understand the viewpoints of the participants (women receiving and students providing care) regarding the care. Combining quantitative and qualitative data will be done in the stages of data collection, analysis, and interpretation (Fig. 1).

2.2. Setting

Final (the fourth) year undergraduate midwifery students of Tabriz University will be the providers of the COC. All these students (28 people) have been invited to take part in the study. Eligible pregnant women will be selected from public health centers in Tabriz, Iran. Tabriz is the capital city of Iran the province with about 1.77 million population and 22 thousand birth a year (18). Cesarean rate in the country is about 51.6% (19).

Quantitative phase (QUAN)

2.2.1. Participants

The participants will be women in the 26th to 29th weeks of pregnancy, a singleton fetus, a history of two vaginal deliveries at most, no history of cesarean section, and a tendency to vaginal birth in one of the public maternity hospitals in Tabriz. Exclusion criteria are as follows: intention to elective cesarean section, presence of major fetal abnormalities, chronic illness, history of high-risk pregnancy or cesarean section, presence of some complications in the current pregnancy, stressful accident during the last three months, lower than secondary school education, lack of access to smartphones and the internet, and concurrent participation in other trials.

2.2.2. Participant recruitment, randomization, and blinding

Public health centers with the largest population of pregnant women will be selected from various socio-economic regions of Tabriz. Number of participants from each region will be determined proportionally. The women will be identified through the national integrated health system (IHS) entitled "SIB". The principal investigator (PI, first author) will call the women to invite potentially eligible ones to participate in the study, and to set up an appointment at their covered health centers. At the center, the PI will give full explanations about the study and assess more detailed eligibility criteria using a checklist. Then, she will obtain written informed consent from the eligible individuals and assess their baseline characteristics. After that, the women will be randomized into two (COC or control) groups.

The random sequence will be generated by the corresponding author (SMAC), who is not involved in the participant recruitment, allocation, and data collection, using blocked randomization stratified by parity (nulliparous/parous), with a randomly varying block size of four and six and 1:1 ratio referring to a computerized program. A central allocation method will be used to conceal the allocation. After participant recruitment and baseline assessment, the stratification factor along with the woman's name and mobile number will be sent to the person who generated the allocation sequence via SMS to determine the allocation group for the woman.

Assigning women to students will be determined via a simple random sampling method. Each student will be assigned as the main COC provider of two pregnant women, and supporter of two pregnant women of the other student. In case of unavailability of a student, the recruited woman will be assigned to the next student in the sequence list. Blinding of the participants and the care providers is not possible due to the nature of the intervention. Also, since the data on the main outcomes will be collected via self-reporting questionnaires, it will be impossible to blind the outcome assessors.

2.2.3. Intervention

During four 4-hour workshops, the students will receive education on the philosophy of COC models, communication skills, assertiveness, critical thinking and problem solving, decision making, physiologic childbirth, evidence-based midwifery practice, and the Iranian integrated maternal health care. Moreover, the students communicate with each other and with their mentor (PI, PhD student in midwifery with 16 years of experience in midwifery teaching) in a group on WhatsApp. The PI will provide and give a handbook containing key points of prenatal, childbirth, and postpartum care to the students besides other important scientific resources such as the Iranian integrated maternal health care guideline.

The participants in the intervention group will get COC from the midwifery students from the time of allocation until six weeks after birth. The continuity of care model will include the following items: 1) receiving at least two in-person antenatal appointments (weeks 26-29 and 35-36), 2) virtual counseling during pregnancy (at least four consultations at intervals of 7-10 days between the two in-person sessions, and then weekly until birth), 3) attending the labor up to 2 hours after birth, 4) in-person appointment at the hospital 12-24 hours after birth, 5) giving at least three virtual consultations after birth (days 3-5, days 7-10 and days 20-30). The supporter student will be present at least in half of the in-person and virtual care sessions. The women to answer could call the students for their non-emergency questions from 8 am to 11 pm, and emergencies and informing about the start of birth 24 hours a day, 7 days a week. The birth of each woman will be done preferably by the student providing her care (supervised by a mentor or a midwife, a

midwifery instructor, or an obstetrician-gynecologist resident). The students will provide additional in-person or virtual care during pregnancy or after birth as required.

The PI will be the students' on-call mentor. To ensure the students' adherence to the study protocol, some special forms have been designed, which the students will complete and provide to the mentor after each care. The mentor will be present in at least one prenatal care session for each student to assess and provide feedback on their midwifery and communication skills. Moreover, with the women' consent, some of the students' consultations at the first sessions will be recorded and provided to the mentor to give the necessary guidance to the students. To ensure women's adherence to the study protocol, the PI will be in contact with the women. One day before virtual or in-person appointments, the PI will coordinate with women and students.

The women in both groups will receive their routine care from either public or private health centers and maternity hospitals. All prenatal, childbirth, and postpartum care in Iran is based on the Iranian integrated maternal health care (20).

Participants will be emphasized that they can withdraw from the study at any stage without giving any reason. For ethical reasons, women in the control group will receive free virtual maternal and child health counseling from the PI from the sixth week (end of study) to the sixth month postpartum.

2.2.4. Outcomes and data collection

The data will be collected in four stages: just before allocation into groups (baseline), 35-36 weeks of pregnancy, 12-24 h after birth, and 40-50 d postpartum. Figure 2 shows the flow diagram of the trial. The primary outcomes including childbirth experience, postpartum depression, and fear of childbirth will be examined 40-50 d postpartum. Secondary outcomes include fear of childbirth and depression in pregnancy, childbirth satisfaction, experience of support and control, maternity care experiences, breastfeeding self-efficacy, type of birth, length of hospital stay until birth, fifth minute Apgar score, and change in hematocrit after birth (Table).

The data collection tools

- The Childbirth Experiences Questionnaire version 2.0 (CEQ2.0): this questionnaire has 23 items in four domains (own capacity, professional support, perceived safety, and participation). The 20 items of the questionnaire are completed on a 4-point Likert scale, and three items are completed as a visual analogue scale (VAS), and then converted to quantitative values of one to four: scores 0-40 (score 1), scores 41-60 (score 2), 61-80 (score 3) and 81-100 (score 4). Items with negative meanings are scored in reverse. The average high score in this questionnaire means a more positive experience of childbirth (21). Psychometrics of this tool has been performed and approved for use among the Iranian population, Cronbach's alpha is 0.93 and intraclass correlation coefficient (ICC) of 0.97 (22).
- The Edinburgh Postnatal Depression Scale (EPDS): this questionnaire for measuring depression during pregnancy and postpartum has ten four-point questions (0 to 3), and a total score between 0 and 30 (23). Psychometrics of this scale has been carried out for the Iranian population with Cronbach's alpha of 0.77 and ICC 0.80 in the 6-8 weeks postpartum (24).
- The Wijma Delivery Expectancy/Experience Questionnaire (W-DEQ-B): This will be used to determine women’s experience with the delivery. It measures the mother's thoughts and feelings during delivery via 33 items on a 6-point Likert scale with a total score between 0 to 165; higher scores show more fear (25). The Iranian version contains 33 items, and its Cronbach's alpha is 0.83 and ICC 0.99 (26).

Other questionnaires used to evaluate baseline characteristics and secondary outcomes are as follows:

- The form of demographic and obstetric characteristics: this form questions the education and occupation of the women and their husbands, adequacy of household income and housing status, number of pregnancies, deliveries and, abortions, planning for pregnancy, history of infertility, and the primary caregiver (midwife/obstetricians) during pregnancy.
- The Wijma Delivery Expectancy/Experience Questionnaire A (W-DEQ-A): will be used to determine the fear of childbirth in pregnancy (25). Its psychometrics has been performed in the Iranian population with Cronbach's alpha of 0.91 (27).

- The Women's experiences of maternity care (EMC): the questionnaire examines the mothers' experience of the care received in three phases of pregnancy, childbirth, and postpartum in 12 items in each phase on a 5-point Likert scale (28). The psychometrics of this questionnaire in Iranian women is on doing as an approved research project (Grant no: 69938); after confirming its validity, it will be used in this study.
- The Scale for Measuring Maternal Satisfaction in Normal and Caesarean Birth (SMMS-normal birth and SMMS-caesarean birth): will be used to determine women's satisfaction in vaginal delivery (43 items/ranged 43-215) or cesarean section (42 items/ranged 42-210). Higher scores show more satisfaction among mothers (29). Psychometrics of this scale has been performed and approved for use in the Iranian population with Cronbach's alpha for vaginal 0.89 and for cesarean 0.84 (30).
- The Perceived Support and Control in Birth scale (SCIB): the original version has 33 items in three subscales. The items are answered on a 5-point Likert scale, where higher scores show greater control and support (31). Iranian version of this scale has 31 items. The paper on its psychometrics is under publication.
- The Breastfeeding Self-Efficacy Scale (BSES): this has 33 items in the 5-point Likert score in 33-165 range, where higher scores show greater self-efficacy (32). Psychometrics of this scale has been confirmed in Iran with Cronbach's alpha of 0.82 (33).
- The type of birth, the length of hospital stays until birth, the fifth minute Apgar score, and the difference between hematocrit after and before birth will be extracted through birth files.
- The content and face validity of the questionnaire on socio-demographic and obstetric, childbirth, and neonatal characteristics (Supplementary material 1) will be determined by a survey of at least seven experts. Persian version of all scales used in this study has been validated in Iran. Cronbach's alpha will be used to determine the internal consistency of the scales. The women will be given the PI's phone number to report any important adverse events (such as amniotic fluid leakage or bleeding) just after the event.

2.2.5. Sample size

G-Power software was used to calculate the sample size. Based on the study by Ghanbari-Homayi et al. (2019) (34) considering the mean score of childbirth experience 2.71 and standard deviation 0.73, M2 = 3.25, (assuming a 20% increase resulted from the intervention), $\alpha = 0.05$, SD1 = SD2, power = 90%, and 15% probability of dropout, the calculated sample size was 46 individuals for each group. This sample size covers the other primary outcomes as well with higher than 80% power.

2.2.6. Data management and analysis

The data analysis will be done after the end of data collection, using SPSS-ver 25. A range check will be used to examine the accuracy of the data entered. Also, another person will check the accuracy of data entry in 10% of randomly selected cases.

We will do our best to prevent loss to follow-up by taking multiple phone numbers from the participants and their families, and by frequent follow-up. Furthermore, we will check the questionnaires just after filling them and ask the participants to fill in any missing items. Missing data will be imputed using multiple imputations.

Data will be analyzed primarily using the modified intention-to-treat (ITT) principle with excluding those with no possibility to assess the outcome. As an additional analysis, a per-protocol analysis will be done by excluding the samples not receiving at least 50% of the full package of the intervention. We will not have an interim analysis.

We will use the Kolmogorov–Smirnov test to assess the normal distribution of the quantitative data. The analysis of covariance (ANCOVA) will be used to compare two groups regarding scores of normally distributed quantitative outcomes; and logistic regression for the qualitative outcomes, adjusted for the baseline values and stratification factor if available. For abnormally distributed quantitative outcomes, Mann-Whitney U-test will be used to compare the groups regarding change score from the baseline value (if available) or the post-intervention score.

2.3. Qualitative phase (qual)

2.3.1. Data collection

Semi-structured individual interviews with the women will be conducted in three stages; 35 to 36 weeks of gestation, the first week after birth, and 40 to 50 days after birth; and with students after the COC for at least one woman is over.

Interviews with the women and the students will be carried out by a trained person (PI) at a convenient place. The initial interview guides include a few broad questions focusing on women's experiences of obtaining COC from midwifery students, and students' experiences of providing COC. As a pilot, at least two pregnant women and two students will be interviewed and their feedback will be used to improve the interview guide. With the interviewee's consent, the interviews will be recorded by an electronic tape recorder and non-verbal cues will be written down.

2.3.2. Sample selection and sample size

Participants will be selected through a purposive sampling approach. Selection of the women and students will be done with maximum diversity; diversity of women in terms of age, parity, education, the person in charge of birth, type of birth, location, and time of birth, and diversity of students in terms of factors such as residence (dormitory/non-dormitory), grade point average, performing or not performing the birth by the student or her supporter. Sample recruitment will be continued until no new code is received (saturation).

2.3.3. Data analysis

The recorded interviews will be transcribed verbatim immediately after each interview. Observational field notes will be incorporated into the data. Data analysis will be done immediately after the transcription of each interview. Thematic analysis will be used following the six-phase approach described by Clarke and Braun Clarke (2013) (35). The qualitative data analysis software MAXQDA will be used to facilitate the coding process. To ensure credibility, we will use some strategies including prolonged engagement with data, persistent observation, and member check. Furthermore, additional in-person or virtual interviews

with the interviewees and/or the focus group discussions will be done to clarify or enrich the data, if necessary. Three supervisors will closely check the process from coding to theme generation, and any disagreement will be settled in the monthly meetings. For transferability, participants with maximum diversity will be selected, and the characteristics of participants will be precisely described. Also, the results will be given to three women with recent childbirth experience who did not participate in the research to judge the similarity of the results with their own experiences. To ensure dependability and confirmability, all stages of the research process will be precisely recorded and transparently reported. We will also use different data collection methods such as interview and note-taking in the field.

2.4. Mixed methods

The study will be conducted as mixed with the embedded experimental model. The priority of this model is the quantitative methodology, and qualitative data is included within the quantitative methodology (36). In this study, the combination will be carried out in the stages of data collection, analysis, and interpretation (Fig 1).

3. Discussion

This study is the first that will be conducted in the field of COC by midwifery students in Iran and, to our knowledge, is the first interventional study in the world to determine the effectiveness of the COC model. A "mixed-methods" approach will be used to determine and explain the different dimensions of the implementation of this care. The mixed approach will provide the key information on how to implement the care to get the high advantages.

In a systematic review, it is stated that 5% of Iranian nulliparous and 53% of parous women tend to have a cesarean section, and the reason for this difference is mainly due to the negative childbirth experiences; women stated that they do not prefer vaginal birth due to the following factors: not being able to trust the caregiver, lack of a skilled and experienced obstetrician/midwife, lack of respectful and supportive communication with them, and the mistreatment of women (37).

Currently, the rate of cesarean section in our country is much higher than international standards. Unpleasant experiences of childbirth and distrust of care providers have a key role in the reluctance to have a vaginal birth. Despite valid scientific evidence of the effectiveness of women-centered and COC, care is provided with a biomedical approach. The country's midwifery educational system uses traditional training methods and is incapable to train skilled and experienced midwives in the field of woman-centered care. We hope that the study findings can provide useful information for the improvement of our education system and our maternity care.

Study Status

The trial was registered prospectively on 30 Apr 2022. Recruitment of care provider students started on 25 June 2022. Twenty-five out of 28 potentially eligible students consented to participate in this study. Recruitment of the women was started on 2 Nov 2022 and is expected to end on Apr 2023 and the follow-up end on Sep 2023.

Ethics and dissemination

To ensure confidentiality, the questionnaires will be identified with a code. Only the PI and the main supervisor will have access to the identifiable details of the participants. This information will be provided to the ethics committee or other research team members in case of a convincing reason. Any important protocol modification will be notified to the scientific and ethical committees and written permission will be obtained.

All research results will be published in international journals. A summary of the results (as scientific news) will also be available to the participants, the public, health professionals, and policy makers in the form of knowledge translation approved by the Irian Ministry of Health and Medical Education.

Contributors

SMA and EJ contributed substantially to the conception and design of the study. SMA and LD provided the qualitative design. EJ wrote the first draft of this study protocol. All authors substantially contributed

to the design of the study, and reviewed and commented on the first draft of the protocol and the manuscript. All authors read and approved the final manuscript.

Funding

The Vice-Chancellor for Research of the Tabriz University, Tabriz, Iran funded this study. The funding agency had no role in the study design, writing this report, and deciding where to submit the manuscript. It will also have no role in participant recruitment; data collection, analysis, and interpretation; writing final report/s, and deciding where to submit the report/s.

Conmpeting interests

None to declare.

Patient consent

Obtained.

Ethics approval

We obtained ethical approval from the ethics committee of Tabriz University of Medical Sciences IR.TBZMED.REC.1401.049).

Data sharing statement

All deidentified participant data sets will be available for researchers just after publishing the results.

Acknowledgements

We thank the Tabriz University for the scientific and financial support.

Supplementary materials

Suppl material 1: Socio-demographic and obstetric, childbirth, and neonatal characteristics questionnaire.

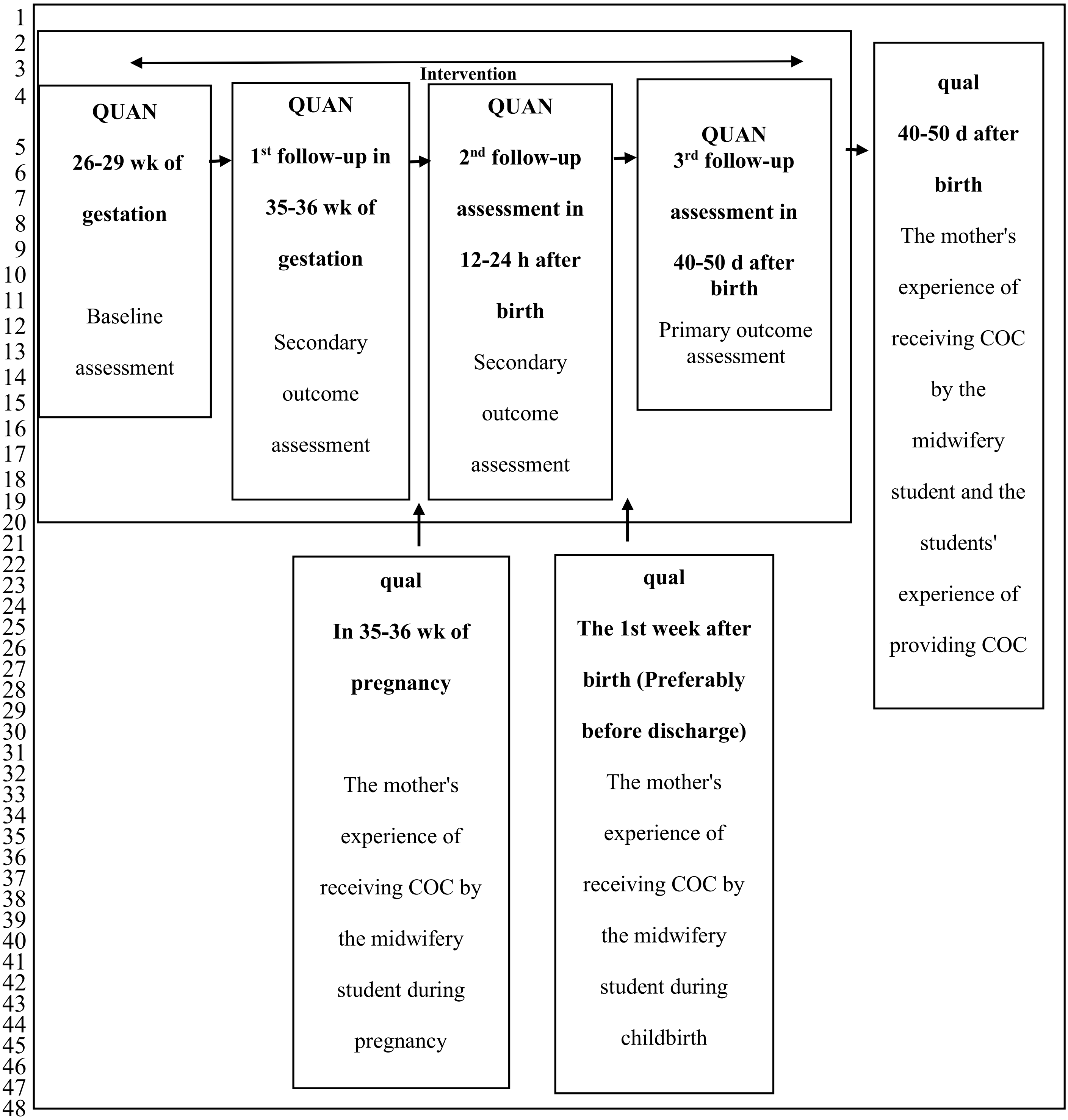

wk: week, COC: continuity of care, QUAN: quatitative, qual: qualitative

Fig 1 Quantitative and qualitative data collection framework

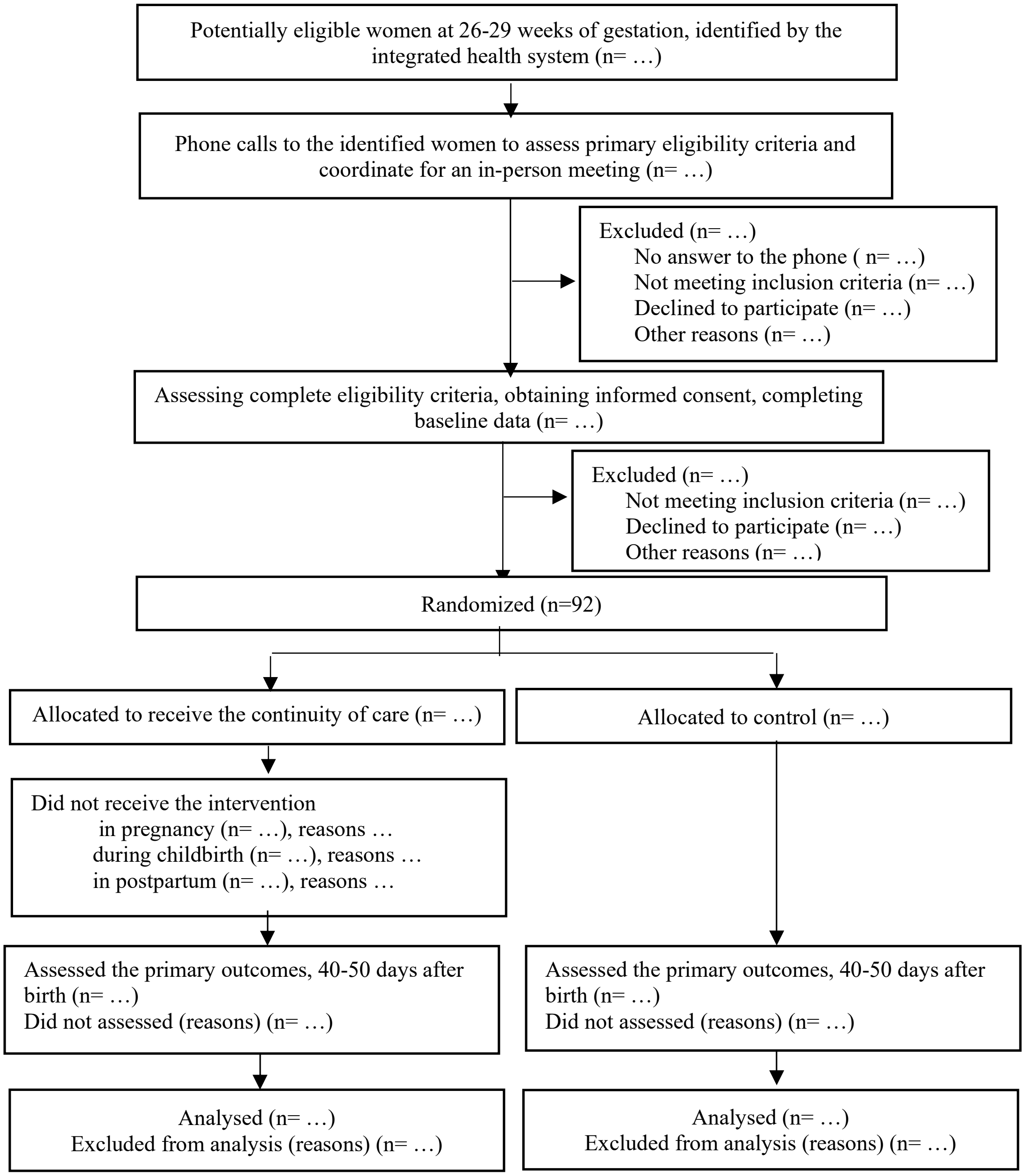

Fig 2. Flow diagram of the trial

Table. Schedule of enrolment, interventions and assessments of the trial

	Primary screening	Enrolment & allocation	Post-allocation		
Time-point	-t1	0<br>(26 to 29 wk of gestation)	T1<br>(35- 36 wk of gestation)	T2<br>(12 to 24 h after birth)	T3<br>(40-50 d after birth
**ENROLMENT:**					
Eligibility screen	x	x			
Informed consent		x			
Allocation		x			
**INTERVENTIONS:**					
Intervention: continuity of care by midwifery students		←	—	—	→
Control: no intervention					
**ASSESSMENTS**					
Demographic and obstetrics characteristics		x			
Wijma Delivery Expectancy/Experience Questionnaire A (W-DEQ-A)		x	x		
W-DEQ-B					x
Edinburgh Postnatal Depression Scale (EPDS)		x	x		x
Scale for Measuring Maternal Satisfaction (SMMS) in normal and Caesarean birth				x	
Perceived Support and Control in Birth scale (SCIB)				x	
Type of birth, length of hospital stays until birth, 5th min Apgar, change in hematocrit after and before birth				x	
Childbirth Experiences Questionnaire version 2.0 (CEQ2.0)					x

Women's experiences of Maternity care (EMC)				
During pregnancy	x	x		x
During labor and birth			x	x
Postnatal care				x
Breastfeeding Self-Efficacy Scale (BSES)				x

References

1. Dowswell T, Carroli G, Duley L, Gates S, Gülmezoglu AM, Khan-Neelofur D, et al. Alternative versus standard packages of antenatal care for low-risk pregnancy. The Cochrane database of systematic reviews. 2015;2015(7):Cd000934.
2. Cummins A, Coddington R, Fox D, Symon A. Exploring the qualities of midwifery-led continuity of care in Australia (MiLCCA) using the quality maternal and newborn care framework. Women and birth : journal of the Australian College of Midwives. 2020;33(2):125-34.
3. Sandall J, Soltani H, Gates S, Shennan A, Devane D. Midwife-led continuity models versus other models of care for childbearing women. The Cochrane database of systematic reviews. 2016;4(4):Cd004667.
4. Sandall J, Soltani H, Gates S, Shennan A, Devane D. Midwife-led continuity models versus other models of care for childbearing women. The Cochrane database of systematic reviews. 2015(9):Cd004667.
5. Moghasemi S, Vedadhir A, Simbar M. Models for providing midwifery care and its challenges in the context of Iran. Journal of Holistic Nursing And Midwifery. 2018;28(1):64-74.
6. Shahinfar S, Abedi P, Najafian M, Abbaspoor Z, Mohammadi E, Alianmoghaddam N. Women's perception of continuity of team midwifery care in Iran: a qualitative content analysis. BMC pregnancy and childbirth. 2021;21(1):173.
7. Firouznia R, Dargahi H, Jafari Koshki T, Khaledian Z. Challenges of Iranian Maternal Health Program from Midwives' Perspectives: A Qualitative Study. Jundishapur Journal of Health Sciences. 2019;11(3).
8. Luyben A, Barger M, Avery M, Bharj KK, O'Connell R, Fleming V, et al. Exploring global recognition of quality midwifery education: Vision or fiction? Women and birth : journal of the Australian College of Midwives. 2017;30(3):184-92.
9. Tickle N, Sidebotham M, Fenwick J, Gamble J. Women's experiences of having a Bachelor of Midwifery student provide continuity of care. Women and birth : journal of the Australian College of Midwives. 2016;29(3):245-51.
10. Hainsworth N, Dowse E, Ebert L, Foureur M. 'Continuity of Care Experiences' within pre-registration midwifery education programs: A scoping review. Women and birth : journal of the Australian College of Midwives. 2021;34(6):514-30.
11. Carter J, Sidebotham M, Dietsch E. Prepared and motivated to work in midwifery continuity of care? A descriptive analysis of midwifery students' perspectives. Women and birth : journal of the Australian College of Midwives. 2021.

12. Gamble J, Sidebotham M, Gilkison A, Davis D, Sweet L. Acknowledging the primacy of continuity of care experiences in midwifery education. Women and birth : journal of the Australian College of Midwives. 2020;33(2):111-8.
13. Tickle N, Gamble J, Creedy DK. Women's reports of satisfaction and respect with continuity of care experiences by students: Findings from a routine, online survey. Women and birth : journal of the Australian College of Midwives. 2021;34(6):e592-e8.
14. Dahlberg U, Aune I. The woman's birth experience---the effect of interpersonal relationships and continuity of care. Midwifery. 2013;29(4):407-15.
15. Kelly J, West R, Gamble J, Sidebotham M, Carson V, Duffy E. 'She knows how we feel': Australian Aboriginal and Torres Strait Islander childbearing women's experience of Continuity of Care with an Australian Aboriginal and Torres Strait Islander midwifery student. Women and birth : journal of the Australian College of Midwives. 2014;27(3):157-62.
16. Tickle N, Gamble J, Creedy DK. Clinical outcomes for women who had continuity of care experiences with midwifery students. Women and birth : journal of the Australian College of Midwives. 2021.
17. Rildayani R, Nurjannah N, Saputra I, Yeni CM, Usman S. The Effect of the Comprehensive Midwifery Care Model with the One Student One Client (OSOC) Approach to Birth Outcomes in North Aceh Regency. Budapest International Research and Critics Institute (BIRCI-Journal): Humanities and Social Sciences. 2020;3(3):1676-82.
18. Mohammadi S. Demographic statistics year 2022. 2022.
19. Pourshirazi M, Heidarzadeh M, Taheri M, Esmaily H, Babaey F, Talkhi N, et al. Cesarean delivery in Iran: a population-based analysis using the Robson classification system. BMC pregnancy and childbirth. 2022;22(1):185.
20. Kharaghani R, Shariati M, Yunesian M, Keramat A, Moghisi A. The Iranian integrated maternal health care guideline based on evidence-based medicine and American guidelines: A comparative study. Mod Care J. 2016;13(2):e9455.
21. Walker KF, Dencker A, Thornton JG. Childbirth experience questionnaire 2: Validating its use in the United Kingdom. European journal of obstetrics & gynecology and reproductive biology: X. 2020;5:100097.
22. Ghanbari-Homayi S, Dencker A, Fardiazar Z, Jafarabadi MA, Mohammad-Alizadeh-Charandabi S, Meedya S, et al. Validation of the Iranian version of the childbirth experience questionnaire 2.0. BMC pregnancy and childbirth. 2019;19(1):465.
23. Cox JL, Holden JM, Sagovsky R. Detection of postnatal depression. Development of the 10-item Edinburgh Postnatal Depression Scale. Br J Psychiatry. 1987;150:782-6.
24. Montazeri A, Torkan B, Omidvari S. The Edinburgh Postnatal Depression Scale (EPDS): translation and validation study of the Iranian version. BMC psychiatry. 2007;7:11.
25. Wijma K, Alehagen S, Wijma B. Development of the Delivery Fear Scale. Journal of psychosomatic obstetrics and gynaecology. 2002;23(2):97-107.
26. Abbaspoor Z, Haghighizadeh MH, Abedi P. Psychometric properties of the Iranian version of Wijma delivery expectancy/experience questionnaire in women who experience fear of childbirth: version B. Journal of OBGYN. 2021;8(1):39-45.
27. Mortazavi F. Validity and reliability of the Farsi version of Wijma delivery expectancy questionnaire: an exploratory and confirmatory factor analysis. Electronic physician. 2017;9(6):4606-15.

28. Redshaw M, Martin CR, Savage-McGlynn E, Harrison S. Women's experiences of maternity care in England: preliminary development of a standard measure. BMC pregnancy and childbirth. 2019;19(1):167.
29. Gungor I, Beji NK. Development and psychometric testing of the scales for measuring maternal satisfaction in normal and caesarean birth. Midwifery. 2012;28(3):348-57.
30. Pakari N, Zahrani ST, Nasiri M, Mahmoodi Z. Persian translation and psychometric testing of the scales for measuring maternal satisfaction in normal and caesarean birth. Biosciences Biotechnology Research Asia. 2016;13(1):339-46.
31. Ford E, Ayers S, Wright DB. Measurement of maternal perceptions of support and control in birth (SCIB). Journal of women's health (2002). 2009;18(2):245-52.
32. Dennis CL, Faux S. Development and psychometric testing of the Breastfeeding Self-Efficacy Scale. Res Nurs Health. 1999;22(5):399-409.
33. Varaei S, Mehrdad N, Bahrani N. The Relationship between Self-efficacy and Breastfeeding, Tehran, Iran. Hayat. 2009;15(3).
34. Ghanbari-Homayi S, Fardiazar Z, Meedya S, Mohammad-Alizadeh-Charandabi S, Asghari-Jafarabadi M, Mohammadi E, et al. Predictors of traumatic birth experience among a group of Iranian primipara women: a cross sectional study. BMC pregnancy and childbirth. 2019;19(1):182.
35. Clarke V, Braun V. Teaching thematic analysis: Overcoming challenges and developing strategies for effective learning. The psychologist. 2013;26(2).
36. Schoonenboom J, Johnson RB. How to Construct a Mixed Methods Research Design. Kolner Z Soz Sozpsychol. 2017;69(Suppl 2):107-31.
37. Shirzad M, Shakibazadeh E, Hajimiri K, Betran AP, Jahanfar S, Bohren MA, et al. Prevalence of and reasons for women's, family members', and health professionals' preferences for cesarean section in Iran: a mixed-methods systematic review. Reproductive health. 2021;18(1):3.
