# Supplementary material for: Effect of midwifery students’ continuity of care program on women’s experiences of maternity care: A randomised controlled trial
Source: PLoS One. 2026 Jul 24;21(7):e0353118. doi: 10.1371/journal.pone.0353118 (PMC13399285; doi:10.1371/journal.pone.0353118)
Supplement: S3 Appendix — Persian version. (PDF) [file pone.0353118.s003.pdf]

بسمه تعالی

"طرح پیشنهادی تحقیق"

عنوان فارسی طرح: اجرا و ارزیابی مدل مراقبت مستمر توسط دانشجویان مامایی در دوران بارداری، حین و بعد از زایمان: یک مطالعه ی ترکیبی با مدل تجربی تو در تو

عنوان انگلیسی طرح: Implementation and evaluation of continuous care model by midwifery students during pregnancy, childbirth and postpartum: a mixed-methods design with Embedded Experimental Model

کد رهگیری پژوهان: ۶۹۶۶۵

نام و نام خانوادگی مجری/ استاد راهنمای اول: سکینه محمد علیزاده چرندابی

نوع طرح: کاربردی

۱. غیر پایاننامه
۲. پایاننامه

محل اجرای طرح: دانشکده: دانشکده پرستاری و مامایی تبریز **مرکز تحقیقاتی**:** گروه مامایی

---

* نشانی: تبریز – دانشگاه علوم پزشکی تبریز– سازمان مرکزی شماره ۲ – معاونت تحقیقات و فناوری – تلفن : ۳۳۳۵۷۳۱۴

** در صورتی که طرح تحقیقاتی پایاننامه ای، در یکی از مراکز تحقیقاتی دانشگاه انجام خواهد شد قبل از ارسال طرح به دانشکده مربوطه، تایید اولیه مرکز ذیربط ضروری است. بدیهی است ذکر آدرس دقیق مرکز تحقیقات محل اجرای طرح برای نفر اول یا نویسنده مسئول مقاله / مقالات حاصل الزامی است.

قسمت اول

۱- اطلاعات مربوط به مجری / استاد راهنما :

نام و نام خانوادگی	سکینه محمد علیزاده چرندابی
نام پدر	علی اکبر
کد ملی	۱۳۷۸۹۸۹۰۷۴

نشانی و تلفن محل کار	تبریز دانشکده پرستاری و مامایی -
مرتبه علمی	استاد
رشته تحصیلی و تخصصی	دکتری تخصصی PhD - دکترای بهداشت باروری
تاریخ دریافت تاییدیه کار باحیوانات آزمایشگاهی(مدت اعتبار ۵ سال)	
شماره حساب بانک رفاه	۲۴۱۸۹۱۹۴۲
تلفن همراه	۰۹۱۴۳۱۳۶۲۷۶
پست الکترونیک	alizades@tbzmed.ac.ir

۲- جدول مشخصات دانشجو (در صورتی که طرح پایاننامه است تکمیل جدول زیر الزامی است)

نام و نام خانوادگی دانشجو	الهام جعفری
مقطع و رشته تحصیلی	دکتری تخصصی PhD - مامایی
دانشکده	دانشکده پرستاری و مامائی دانشکده پرستاری و مامایی تبریز
دانشگاه	دانشگاه علوم پزشکی تبریز
تلفن همراه	۰۹۱۲۲۴۱۳۸۹۷
پست الکترونیک	elhamdjafari@gmail.com

۳- راهنمای تکمیل طرح پیشنهادی تحقیق را مطالعه کردم و آنها را رعایت میکنم.

Link راهنمای تکمیل طرح پیشنهادی تحقیق

٤- لیست پژوهش های مرتبط با موضوع طرح پیشنهادی که مجری / استاد راهنما در آن مشارکت داشته است:

عنوان طرح	نام مجری	نوع مشارکت	زمان شروع	درصد پیشرفت کار	ملاحظات

قسمت دوم

۱- خلاصه مشخصات طرح

نام و نام خانوادگی مجری / استاد راهنما:	سکینه محمد علیزاده چرندابی
نوع مطالعه	کارآزمایی بالینی (Clinical Trial)
اولویت طرح	روشهای توسعه و ارتقاء آموزش در علوم پزشکی، مدیریت آموزشی و برنامه‌ریزی درسی
نوع طرح	کاربردی
عنوان طرح: اجرا و ارزیابی مدل مراقبت مستمر توسط دانشجویان مامایی در دوران بارداری، حین و بعد از زایمان: یک مطالعه ی ترکیبی با مدل تجربی تو در تو	
Title: Implementation and evaluation of continuous care model by midwifery students during pregnancy, childbirth and postpartum: a mixed-methods design with Embedded Experimental Model	

محل یا محل های اجرای طرح (دانشکده/مرکز تحقیقاتی):	دانشکده پرستاری و مامائی
مدت اجرای طرح (بر حسب ماه):	۱۸ ماه
هزینه کلی طرح (به ریال):	۲۶۷,۱۲۰,۰۰۰ ریال

خلاصه روش اجرا:

خلاصه روش اجرا: هدف این یک مطالعه ی ترکیبی با مدل تجربی تو در تو، اجرا و ارزیابی مدل مراقبت مستمر توسط دانشجویان مامایی با استفاده از تلفیق داده های بخش کمی و کیفی جهت فراهم¬سازی یافته های غنی تر می¬باشد. در این مطالعه، طرح کیفی در درون طرح کمی قرار دارد و یافته های این دو طرح به صورت یکی در درون دیگری به عنوان نقش مکمی مورد تجزیه و تحلیل قرار می گیرند. قسمت کمی پژوهش از نوع کارآزمایی بالینی تصادفی سازی شده می باشد که با اهداف اولیه ی تعیین تاثیر اجرای مدل مراقبت مستمر توسط دانشجویان مامایی در دوران بارداری، حین و پس از زایمان بر تجربه ی زایمان، ترس از زایمان و افسردگی پس از زایمان در زنان تحت پوشش مراکز سلامت شهر تبریز انجام خواهد شد. هدف بخش کیفی تبیین دیدگاه زنان از اجرای مدل مراقبت مستمر توسط دانشجویان مامایی و نیز تجربیات دانشجویان ارائه دهنده مراقبت مستمر خواهد بود. نمونه ی بخش کمی شامل ۹۲ زن با سن بارداری ۲۶ تا ۲۹ هفته، بدون سابقه ی زایمان یا با سابقه ی حداکثر دو زایمان واژینال، بدون سابقه ی سزارین، فاقد بیماری های زمینه ای شناخته شده، فاقد سابقه ی بارداری پرخطر و عوارض شناخته شده بارداری بوده که تمایل به زایمان واژینال در یکی از زایشگاههای وابسته به دانشگاه علوم پزشکی یا تامین اجتماعی شهر تبریز دارند. پژوهشگر پس از اخذ کد اخلاق و ثبت کارآزمایی در مرکز ثبت کارآزمایی بالینی ایران، اقدام به نمونه گیری خواهد کرد. مراکز سلامتی که بیشترین جمعیت زنان باردار را تحت پوشش دارند از مناطق مختلف شهر انتخاب خواهند شد و سپس از هر منطقه به نسبت جمعیت زنان باردار تحت پوشش، تعداد نمونه به صورت نسبتی مشخص خواهد شد. شایان ذکر است که چون در جامعه ی ما افراد مرفه عموما از خدمات مراکز سلامت برای مراقبتهای بارداری و زایمان استفاده نمی کنند و تمایلی به زایمان در بیمارستان های دولتی و تامین اجتماعی ندارند، لذا این مساله جزو محدودیت های طرح ما می باشد. زنان باردار تحت پوشش مراکز سلامت منتخب شهر تبریز (از مناطق مختلف شهر) که شرایط اولیه ورود به مطالعه را دارند، با استفاده از سامانه سیب شناسایی کرده، طی تماس تلفنی، پس از بررسی برخی دیگر از شرایط ورود به مطالعه و توضیح مختصر اهداف و روش مطالعه، افراد بالقوه حائز شرایط را جهت شرکت در مطالعه به مرکز تحت پوشش دعوت خواهد کرد. در مرکز، بعد از ارائه ی توضیحات کامل در خصوص مطالعه، اهداف و نحوه ی اجرای مطالعه و بررسی دقیقتر شرایط ورود و خروج از مطالعه، از افراد حائز شرایط برای شرکت در مطالعه فرم رضایت نامه آگاهانه ی کتبی اخذ شده و افراد پس از تکمیل پرسشنامه های مربوط به مشخصات پایه ای از جمله فرم مشخصات دموگرافیک و مامایی، پرسشنامه های ترس از زایمان (دوران بارداری)،

افسردگی و تجارب مراقبت های مادری (بخش دوران بارداری) با روش تخصیص تصادفی بلوکه بندی طبقه بندی شده (بر اساس نولی پار یا مولتی پار بودن) با اندازه بلوک های ۴ و ۶ تایی با نسبت تخصیص ۱:۱ در دو گروه دریافت کننده مراقبت مستمر توسط دانشجو یا کنترل تخصیص خواهند یافت. توالی تخصیص زنان به دانشجویان هم بصورت تصادفی ساده تعیین خواهد شد و به هر دانشجوی مامایی (به عنوان مراقب اصلی) دو مادر باردار اختصاص داده خواهد شد و در ضمن هر دانشجو، پشتیبان دو مادر باردار دانشجوی دیگر خواهد بود. توالی تخصیص زنان باردار و دانشجویان توسط فرد غیر درگیر در نمونه گیری تعیین خواهد شد. تمام مراحل انتخاب نمونه ها، اخذ رضایت آگاهانه، جمع آوری داده های پایه ای و پیامدهای اولیه توسط دانشجوی پژوهشگر اصلی (صاحب پایان نامه) انجام خواهد شد. جهت پنهان سازی تخصیص از روش مرکزی (central) استفاده خواهد شد. به این صورت که پس از اخذ رضایت نامه آگاهانه و تکمیل مشخصات پایه¬ای، دانشجوی پژوهشگر اصلی تعداد، پاریته، نام و شماره موبایل زنان باردار را به فرد غیردرگیر در نمونه گیری و جمع آوری داده¬ها از طریق پیامک ارسال خواهد نمود و وی با توجه به توالی تخصیص از قبل تعیین شده، گروه قرارگیری زن و دانشجوی ارائه دهنده مراقبت از وی را مشخص خواهد نمود. ارائه دهندگان اصلی مراقبت مستمر مادران (از هفته ۲۹-۲۶ بارداری تا شش هفته پس از زایمان)، دانشجویان ترم شش/هفت مامایی دانشگاه علوم پزشکی تبریز (و در صورت نیاز دانشگاه آزاد تبریز) می¬باشند که فرم رضایت آگاهانه ی کتبی جهت شرکت در مطالعه را امضا نموده و و طی چهارجلسه ی کارگاهی چهار ساعته، آموزشهای ویژه¬ از جمله فلسفه ی مراقبتهای مامایی مستمر زن محور، مهارت های ارتباطی و جرات ورزی، تفکر انتقادی و حل مساله، مهارت تصمیم گیری، زایمان فیزیولوژیک، مامایی مدرن مبتنی بر شواهد، مروری بر مراقبتهای ادغام یافته سلامت مادران در بارداری، زایمان و پس از زایمان را قبل از ورود به مطالعه دریافت نموده باشند. در تمام طول مدت پژوهش با تشکیل گروه واتس اپ، دانشجویان با یکدیگر و با دانشجوی پژوهشگر اصلی در ارتباط خواهند بود و تحت منتورشیپی دانشجوی پژوهشگر اصلی (با ۱۵ سال سابقه در آموزش مامایی و عضو هسته آموزشی زایمان فیزیولوژیک) قرار خواهند گرفت. تمام مطالب آموزشی مطرح شده در دوره کارگاهی، محتوای مربوط به جلسات مراقبت و همچنین سوالات مطرح شده توسط دانشجویان در سیر ارائه ی مراقبت به مادران در گروه تمرین و تکرار خواهد شد. پژوهشگر اصلی بعنوان منتورشیپ دانشجویان به صورت آنکالی پاسخ گوی دانشجویان خواهد بود. همچنین فرم هایی طراحی شده است که دانشجویان پس از ارائه ی هر مراقبت به مادر، آنها را تکمیل کرده و در اختیار منتور قرار خواهد داد تا نقاط قوت و ضعف دانشجو در ارائه ی مراقبت به وی بازخورد داده شود و در صورت لزوم اقدامات ضروری برای مادر اصلاح شود. همچنین سعی خواهد شد در مراقبت های اولیه که هر دانشجو ارائه می دهد، منتور به صورت حضوری یا مجازی حضور یابد و نحوه ی ارائه ی مراقبت دانشجو را چه در زمینه ی مهارت مامایی و چه در زمینه ی مهارت ارتباطی ارزیابی کند و به وی فیدبک دهد، چنانچه این مساله مقدور نباشد با رضایت مادران ویس جلسه ی چند مراقبت اولیه هر دانشجو ضبط و در اختیار منتور قرار داده خواهد شد تا راهنمایی لازم برای ادامه ی مراقبت ها به دانشجویان داده شود. زنان در هر دو گروه مورد مطالعه مراقبتهای استاندارد ارائه شده در مراکز سلامت و یک دفترچه ی راهنمای دوران بارداری، زایمان و پس از زایمان را دریافت خواهند نمود. علاوه بر آن، زنان گروه مداخله از زمان ورود به مطالعه تا شش هفته پس از زایمان تحت مراقبت مستمر توسط یک دانشجوی ترم شش/هفت (با پشتیبانی یک دانشجوی دیگر) قرار خواهند گرفت. مراقبت مستمر به صورت دریافت حداقل دو بار مراقبت حضوری (بین هفته ی ۲۹-۲۶ و بین هفته ی ۳۶-۳۵ بارداری) و مشاوره های تلفنی یا تصویری (حداقل چهار مشاوره تلفنی یا تصویری با فواصل ۱۰-۷ روز مابین دو مراقبت حضوری و سپس به صورت هفتگی تا زمان زایمان)، حضور در بالین زن و ارائه ی مراقبت طی لیبر و زایمان تا ۲ ساعت پس از زایمان، ویزیت حضوری ۲۴-۱۲ ساعت پس از زایمان در بیمارستان، دادن حداقل سه مشاوره ی تلفنی یا تصویری (روز ۵-۳ ، روز ۱۰-۷ و روز ۳۰-۲۰) پس از زایمان خواهد بود. دانشجوی اصلی و تا حد امکان پشتیبان وی در جلسات مراقبتی و مشاوره¬ای (حضوری و آنلاین) حضور خواهند داشت، شایان ذکر است که دانشجوی پشتیبان حداقل در نیمی از مراقبت های دوران بارداری حضور خواهد داشت. شماره موبایل دانشجویان جهت پاسخگویی به سوالات احتمالی غیراورژانسی زنان (از ساعت ۸ صبح تا ۱۱ شب) و برای موارد اورژانسی و اطلاع شروع زایمان (به صورت آنکالی ۲۴ ساعته در ۷ روز هفته) در اختیار زنان قرار داده خواهد شد. زنان باردار برای سوالات خود با دانشجوی اصلی و در صورت عدم دسترسی به وی با دانشجوی پشتیبان تماس خواهند گرفت. دانشجوی اصلی ارائه دهنده ی مراقبت به هر مادر، بطور مرتب و بعد از هر مراقبت گزارشی از هر گونه اقدام انجام گرفته برای مادر را به دانشجوی پژوهشگر اصلی ارائه داده و بازخورد خواهد گرفت. سعی خواهد شد که یکی از دانشجویان اصلی مراقبت مستمر یا پشتیبان وی بومی باشد. دانشجوی اصلی ارائه دهنده ی مراقبت به هر مادر (در صورت عدم امکان حضور، پشتیبان وی) از زمان بستری جهت زایمان در بیمارستان در بالین زن تا دو ساعت پس از زایمان حضور خواهد داشت. تا حد امکان زایمان این گروه از مادران توسط

دانشجویان ارائه دهنده مراقبت مستمر (تحت نظارت دانشجوی پژوهشگر اصلی یا هر فرد دیگری اعم از مربی مامایی، ماما یا رزیدنت زنان که مسئولیت زایمان را برعهده بگیرد) انجام خواهد گرفت. مراقبت حضوری یا آنلاین اضافی در طی دوران بارداری یا پس از زایمان نیز در صورت نیاز توسط دانشجو/دانشجویان ارائه خواهد شد. پیامدهای اولیه ی مطالعه شامل نمره ی ترس از زایمان، نمره ی تجربه ی زایمان و نمره ی افسردگی پس از زایمان ۵۰-۴۰ روز پس از زایمان به ترتیب با استفاده از پرسشنامه ی انتظار/تجربه ی زایمان ورژن (W-DEQ-B)(B The Wijma delivery (expectancy/experience questionnaire ، پرسشنامه ی تجربه ی زایمان نسخه ی دو (Childbirth Experiences Questionnaire vsersion 2.0 (CEQ2.0)) و پرسشنامه ی افسردگی ادینبورگ(Edinburgh Postnatal Depression Scale (EPDS)) به روش خودگزارش دهی توسط مادران مورد سنجش قرار خواهند گرفت. برای مقایسه ی دو گروه از نظر این پیامدها از ANCOVA با کنترل عامل طبقه بندی (و نمره پایه در صورت وجود) استفاده خواهد شد. در بخش کیفی مطالعه برای تبیین دیدگاه زنان از اجرای مدل مراقبت مستمر توسط دانشجویان مامایی از مصاحبه¬های باز یا نیمه ساختار یافته ی انفرادی در سه مقطع هفته ی ۳۶-۳۵ بارداری، هفته ی اول پس از زایمان (در صورت امکان قبل از ترخیص) و ۵۰-۴۰روز پس از زایمان استفاده خواهد شد. انتخاب زنان با در نظر گرفتن حداکثر تنوع از نظر عواملی همچون سن، پاریتی، سطح تحصیلات زن، عامل اصلی زایمان (دانشجوی ارائه دهنده مراقبت مستمر/ پشتیبان وی/فرد دیگر)، نوع زایمان، محل و زمان زایمان (شیفت صبح/عصر/شب قبل از ساعت ۱۲/ شب بعد از ساعت ۱۲) انجام خواهد شد. جهت تبیین تجربیات دانشجویان ارائه دهنده مراقبت مستمر از مصاحبه¬های بارز یا نیمه ساختاریافته ی انفرادی پس از اتمام ارائه ی مراقبت مستمر دوران بارداری، زایمان و پس از زایمان حداقل برای یک مادر (با رعایت حداکثر تنوع از نظر عواملی همچون سن، خوابگاهی/غیرخوابگاهی بودن، میانگین معدل نیمسالهای تحصیلی قبلی، امکان/عدم امکان انجام زایمان توسط دانشجو و فرد حمایت کننده دانشجو در انجام زایمان) استفاده خواهد شد و در صورت لزوم از بحثهای گروهی متمرکز نیز استفاده خواهد شد. مصاحبه¬ها بصورت حضوری در محلی مناسب برای شرکت¬کنندگان با استفاده از سوالات باز یا نیمه ساختار یافته انجام و ضبط خواهند شد. بلافاصله پس از هر مصاحبه به صورت کلمه به کلمه پیاده خواهد شد. طی مصاحبه¬ها از زبان بدنی شرکت کنندگان نیز یادداشت برداری خواهد شد. در صورت لزوم، جهت شفاف¬سازی یا غنی¬سازی داده¬ها از مصاحبه ی مجدد حضوری یا تلفنی/تصویری هم استفاده خواهد شد. قبل از اجرای مرحله کیفی، سوالات اولیه ی راهنمای مصاحبه با همکاری تیم تحقیق طراحی می¬شوند و دقت خواهد شد سوالات در راستای تجربه ی مادران از مراقبت های مستمر دریافتی و نه تجربه ی صرف باداری و زایمان باشد . تحلیل داده¬ها با استفاده از روش تحلیل محتوای کیفی پس از اولین مصاحبه شروع خواهد شد. برای این منظور، متن به واحدهایی از معانی یا ایده ها تقسیم بندی می شوند، این واحدها که واحد تحلیل نیز نامیده می شوند ممکن است کلمات ویژه و یا ترکیبی از آنها باشند. در مرحله بعد بر اساس قواعد ویژه ای، با حذف متون همانند و کنارگذاشتن موارد تکراری این واحدها محدود و کمی می شوند. سپس در گام بعدی مقولات و طبقات ایجاد می شوند. طبقات می توانند شامل مجموعه ای از زیر طبقات با سطوح متفاوت و به صورت انتزاعی باشند و در نهایت در مرحله ی اصلاح نظام طبقات بر اساس داده ها، پژوهشگر نظام طبقات را اصلاح و در صورت نیاز، برخی طبقات را حذف و تعداد دیگری را اضافه خواهد نمود. نمونه¬گیری تا اشباع اطلاعات، یعنی عدم دریافت اطلاعات یا کد جدید ادامه خواهد یافت. از چهار معیار قابلیت اعتبار، قابلیت اعتماد، قابلیت تأیید و قابلیت انتقال¬پذیری برای افزایش صحت و استحکام یافته¬های کیفی استفاده خواهدشد. ترکیب داده های کمی و کیفی در مراحل جمع آوری، تحلیل و تفسیر انجام خواهد گرفت.

چکیده انگلیسی طرح:

The present study has a mixed-methods design with an Embedded Experimental Model with the aim of Implementation and evaluating of continuous care model by midwifery students during pregnancy, childbirth and postpartum. The quantitative part of the research is a randomized clinical trial to determine the effect of the intervention on the childbirth experience, fear of childbirth, and postpartum depression. The aim of the qualitative part is to explore women's views on the implementation of continuous care by midwifery students, as well as to explore the experiences of students providing continuous care. In the experimental section, 92 women with a gestational age of 26 to 29 weeks will be assigned into two groups of intervention and control by stratified

randomized Block Design, after getting written informed consent and baseline assessment. The sequence of assigning women to the students who are going to provide the care will be by simple random sampling. The central method will be used to conceal the allocation. The main providers of care will be the sixth/seventh-semester midwifery students of Tabriz University of Medical Sciences, who have signed a written informed consent form and received the necessary training. The women in the intervention group will receive continuous care from a sixth/seventh-semester student (with the support of another student) from the time of enrollment until six weeks after delivery. Continuous care includes receiving at least two face-to-face care sessions, at least three telephone or video consultations during pregnancy, attending labor and delivery, face-to-face visits 12-24 hours after delivery, giving at least three telephone or video post-delivery consultations. Students will be on call to answer mothers' questions. In the WhatsApp group, students will be in touch with each other and with the mentoring Ph.D. student. In the qualitative part of the study, unstructured or semi-structured in-depth interviews were used in three stages of 35-36 weeks of pregnancy, the first week after delivery, and 40-50 days after delivery to explore the women's view of the implementation of the continuous care model by midwifery students. Also, unstructured or semi-structured in-depth interviews with the students will be done after providing continuous care at least for one woman to explore students' experiences. Data analysis will be done using the qualitative content analysis method. The combination of quantitative and qualitative data will be done in the stages of collection, analysis, and interpretation.

نوآوری و ضرورت اجرای طرح:

تامین سلامت مادران از ضروریات انکارناپذیر هر سیستم سلامتی است. طراحی و اجرای مدل های مراقبتی موثر در دوران بارداری و زایمان در راستای کاهش مرگ و میر مادران و نوزادان و ارتقای سلامت آنها می باشد. مراقبت زنان در مدل های مراقبت زن محور به صورت مستمر و البته منحصر به فرد برای هر زن، با رویکرد ارتقای سیر طبیعی بارداری و زایمان، شناخت و احترام به نیازهای مادر صورت می گیرد. مراقبت مستمر مامایی به مراقبتی مبتنی بر ارتباط با یک ماما (با پشتیبانی یک تا سه ماما) اطلاق می شود که در طی آن مراقبت ها در قالب یک برنامه ی از پیش تعیین شده و همچنین به صورت آنکالی در سراسر دوران بارداری، لیبر و زایمان و تا شش هفته پس از زایمان ارائه می شود. مطالعات نشان داده اند که مدل مراقبت مستمر مامایی با سطح بالاتر رضایت مادران و میزان پایین تر مداخلات زایمانی همراه بوده است. علی رغم وجود مستندات علمی با کیفیت بالا در حمایت از فواید مراقبت مستمر، دسترسی به این مدل مراقبتی حتی در بسیاری از کشورهای پیشرو نیز هنوز محدود بوده و در کشور ما ایران هنوز اجرا نمی شود. تحقیقات نشان داده اند که استاندارهای خدمات مامایی در ایران، نیازمند بازنگری جدی جهت ارائه ی خدمات با کیفیت مطلوب و با مرکزیت زن هستند. از جمله موانع اثربخشی خدمات مامایی ارائه شده در مراکز سلامت می توان به فقدان ارائه ی مراقبتهای مستمر طی بارداری، حین و بعد از زایمان و نقش دانشگاه ها در آموزش ماماهای توانمند و ماهر اشاره کرد. مشکلات موجود در آموزش مامایی می تواند سبب کاهش مهارتهای تئوری و بالینی فارغ التحصیلان مامایی شود که نمود آن حضور کم رنگ و غیر موثر ماماها در عرصه ی سلامت جامعه خواهد بود. اهمیت آموزش نیروی انسانی مجرب در مامایی یک ضرورت جدی و حیاتی است و باید زمینه های پیوند اطلاعات نظری به واقعیت های بالینی به خوبی فراهم شود. حال با توجه به اهمیت مراقبتهای مستمر زن محور، نقش کلیدی ماما در این نوع از مراقبت و لزوم توانمندسازی ماماها در طی دوره آموزشی، به نظر می رسد راهکاری چون شناخت فلسفه ی مراقبتهای مستمر زن محور توسط دانشجویان، طراحی یک مدل تلفیقی یادگیری و یاددهی، ارائه ی مراقبت مستمر توسط دانشجویان با پشتیبانی افراد حرفه ای برای موفقیت و اثربخشی مراقبتهای مستمر به شدت الزامی است. مطابق بررسی تیم تحقیق، مطالعاتی در این زمینه در کشورهای پیشرفته¬ای همچون استرالیا انجام گرفته که همه ی آنها از نوع کیفی یا کمی غیرمداخله¬ای بوده¬اند و ما مطالعه¬ای مداخله¬ای در این زمینه (احتمالا به دلیل روتین بودن آموزش دانشجویان آن کشورها با این مدل مراقبتی) پیدا نکردیم. با توجه

به اهمیت و ضرورت ارتقای سلامت مادران و نوزادان و همچنین توسعه ی حرفه ی مامایی در ایران بر اساس نیازهای جامعه، طراحی مدل های مراقبت مامایی همسو با شواهد علمی و متناسب با فرهنگ، عقاید، قوم شناسی و دانش محلی ایرانی برای ارتقای سلامت مادران، نوزادان، خانواده ها و در نهایت مردم ایران مورد نیاز است. ما در ایران تنها یک مطالعه ی کیفی، آنهم در زمینه ی ارائه ی مراقبتهای مستمر توسط ماماها، پیدا کریم. بنابراین، با توجه به خلاء اطلاعاتی موجود، این مطالعه ی ترکیبی با مدل تجربی تو در تو با هدف اجرا و ارزیابی مدل مراقبت مستمر توسط دانشجویان مامایی در دوران بارداری، حین و بعد از زایمان طراحی شد. امید است نتایج آن در ارتقای جایگاه مامایی در کشورمان در راستای اعتلای سلامت مادران و نوزادان قابل استفاده باشد.

۲- مشخصات افراد

نام و نام خانوادگی	شغل	درجه علمی و رشته تحصیلی	نقش*	نحوه همکاری**	کل ساعات کار برای طرح	حق الزحمه در ساعت (ریال)	جمع (ریال)	گواهی شرکت در کارگاه های اخلاق	تاریخ دریافت تاییدیه کار باحیوانات آزمایشگاهی(مدت اعتبار ۵ سال)	امضا
**مجری و همکاران طرح**										
سکینه محمد علیزاده چرندابی		دکتری تخصصی PhD - دکترای بهداشت باروری	استاد راهنمای اول (آموزشی)	مجری طرح و استاد راهنمای اول، نظارت و همکاری در انتخاب عنوان، تدوین مساله، طراحی متدولوژی، اجرای طرح و تجزیه و تحلیل داده ها و نوشتن گزارش نهایی و نگارش مقاله	۱۵۰	۲۰۰,۰۰۰	۳۰,۰۰۰,۰۰۰	مشاهده		

مژگان میرغفورvند		دکتری تخصصی PhD - آموزش مامایی	مشاور	۵۰	نظارت و همکاری در تدوین بیان مساله، طراحی متدولووژی، اجرای طرح، نوشتن گزارش نهایی و نگارش مقاله	۲۰۰,۰۰۰	۱۰,۰۰۰,۰۰۰	مشاهده		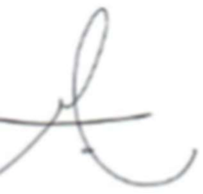 دکتر مژگان میرغفورو [illegible] معاون تحقیقات و فناوری دانشکده پرس [illegible]
لیلا دشمنگیر		دکتری تخصصی PhD - سیاست گذاری سلامت	مشاور	۵۰	نظارت و همکاری در تدوین بیان مساله، طراحی متدولووژی، اجرای طرح، نوشتن گزارش نهایی و نگارش مقاله (به ویژه بخشهای کیفی مطالعه)	۱۷۰,۰۰۰	۸,۵۰۰,۰۰۰	مشاهده		
شمسی عباسعلیزاده		دکتری تخصصی بالینی - زنان و زایمان	استاد راهنما دوم (آموزشی)	۵۰	نظارت و همکاری در انتخاب عنوان، تدوین بیان مساله، طراحی	۲۰۰,۰۰۰	۱۰,۰۰۰,۰۰۰	مشاهده		

				متدولوژی، اجرای طرح و تجزیه و تحلیل داده ها و نوشتن گزارش نهایی و نگارش مقاله					
الهام جعفری		دکتری تخصصی PhD - مامایی	دانشجوی مالک پایان نامه	انتخاب عنوان، تدوین بیان مساله، طراحی متدولوژی، اجرای طرح و تجزیه و تحلیل داده ها و نوشتن گزارش نهایی و نگارش مقاله	۱۰۰	۶۵,۰۰۰	۶,۵۰۰,۰۰۰	مشاهده		
شهلا میدیا		دکتری تخصصی PhD - مامایی	همکار اصلی	کمک در نگارش مقاله	۵۰	۱۷۰,۰۰۰	۸,۵۰۰,۰۰۰	مشاهده		

سایر افراد

* **نقش:** در طرح پایاننامه ای: استاد راهنما / یا مشاور / دانشجو و در طرح غیر پایاننامه ای: مجری / همکار اصلی / همکار مشخص شود.<br>****نحوه همکاری:** بطور دقیق و به تفکیک برای هر یک از افراد در ارتباط با اجرای طرح / پایاننامه ذکر گردد.	جمع هزینه (ریال): ۷۳,۵۰۰,۰۰۰

۳- مقدمه، بیان مسئله و ضرورت اجرای طرح:

ارزش و اهمیت سلامت مادران به دلیل تاثیر مستقیم آن بر نسل های سالم و مولد بعدی در هر جامعه ای مشهود است. بنابراین ارتقای مراقبت از مادران از ضروریات انکارناپذیر هر سیستم سلامتی است (۱). تجربه ی بارداری و زایمان از جمله تجارب ماندگار و تاثیرگذار در زندگی زنان است، چه بسا کسب تجارب مثبت در روند مادرشدن می تواند سبب توانمندسازی زنان و احساس رضایت آنها در طول زندگی شود (۲).

طراحی و اجرای مدل های مراقبتی موثر در دوران بارداری و زایمان یکی از اهداف اصلی سیاست های سلامت در جهان است که نه تنها در راستای کاهش مرگ و میر مادری، بلکه در جهت ارتقای سلامت مادران و نوزادان می باشد (۳). ماماها با ارائه ی مراقبتهای قبل از بارداری، حین بارداری، زایمان و پس از زایمان، فراهم نمودن مراقبت ها برای نوزادان و کودکان، مشارکت در آموزش و مشاوره، تشخیص و ارجاع به موقع انواع ناهنجاری ها در مادر، جنین و نوزاد آنها نقش چشمگیری در سلامت مادران و فرزندان آنها دارند. همچنین ماماها می توانند نقش پررنگی در زایمان ایمن و کاهش نرخ سزارین داشته باشند (۴).

امروزه مدل های مراقبت زن محور، با پروسه ی بارداری و زایمان به عنوان یک فرایند فیزیولوژیک و طبیعی همراه با درگیری عاطفی عمیق مادر برخورد می کنند و مشاهدات گویای آن است که این مدل های مراقبتی می توانند در توانمندسازی مادران و افزایش رضایت آنها و همچنین کاهش عوارض نامطلوب برای مادران و نوزادان نقش داشته باشند (۵). ماماها به عنوان رکن اصلی و لاینفک سیستم برای ارائه ی این مراقبت ها محسوب می‌شوند. در همین راستا مدل های مختلفی برای ارائه ی مراقبتهای ماما محور طراحی و اجرا شده اند. از جمله ی آنها مراقبت مستمر مامایی می‌باشد که مراقبتی مبتنی بر ارتباط با یک ماما (با پشتیبانی یک تا سه ماما) می باشد که در طی آن مراقبت ها در قالب یک برنامه ی از پیش تعیین شده و همچنین به صورت آنکالی در سراسر دوران بارداری، لیبر و زایمان و تا شش هفته پس از زایمان ارائه می شود (۶).

شواهد متقنی وجود دارد که نشان می دهد که همه ی زنان نیاز دارند که به یک مامای آشنا (Known-midwife) دسترسی داشته باشند (۷). اساس و پایه ی مراقبت زن محور ارتباط است. ماهیت و کیفیت ارتباط، قلب پیامدهای مثبت حاصل از مراقبت برای زنان و نوزادان آنها می باشد (۸). اغلب زنانی که تجربه ی دریافت مراقبت مستمر را دارند، گزارش می کنند که دریافت حمایت عاطفی و اجتماعی و تجربه ی اعتماد به ماما در این روند، منجر به افزایش اعتماد به نفس آنها و توانایی زایمان طبیعی، حتی بدون نیاز به روشهای دارویی کاهش درد زایمان شده است (۹). نتایج یک مطالعه کیفی در ایران هم نشان داده است که مراقبت مستمر دوران بارداری، حین و پس از زایمان توسط تیمی از ماماها سبب افزایش خودکارآمدی، افزایش توانایی پذیرش عاطفی زایمان طبیعی، افزایش توانایی مواجهه با موقعیت های پیش بینی نشده، مدیریت بهتر تغییرات روانی و فیزیکی بارداری و رابطه ی موثر بین مادر و ماما سبب اعتماد مادران به ماماها شده است. مادران از ایجاد این شرایط دوستانه و قابل اعتماد بسیار سپاسگزار بودند و بسیاری از آنها اظهار داشتند که از بارداری در آینده نه تنها ترسی ندارند بلکه تجربه ی این مدل مراقبت را برای بارداری بعدی خود و همچنین سایرین توصیه می کنند (۹). مطالعات نشان داده اند که مدل مراقبت مستمر مامایی با سطح بالاتر رضایت مادران و میزان پایین تر مداخلات زایمانی، از دست دادن جنین و مرگ نوزادی همراه بوده است (۷).

اگرچه تعریف رضایت معمولاً امری دشوار است ولی معمولا در قالب برآورده شدن یک نیاز یا انتظار می توان آن را گنجاند (۱۰). درک نیازها و انتظارات زن بدون برقراری رابطه ی عمیق زن و ماما بسیار دشوار است و دشوارتر از آن این است که بتوان بدون برقراری ارتباط صحیح با مادر مراقبت ها را بر اساس تفاوت های فردی تطبیق داد (۱۱). علی رغم وجود مستندات علمی با کیفیت بالا در حمایت از فواید مراقبت مستمر، دسترسی به این مدل مراقبتی حتی در بسیاری از کشورهای پیشرو در ارائه ی این مدل ها نیز هنوز محدود است (۱۲).

تعریف حرفه ی مامایی و شرح وظایف آن در ایران مطابق با تعریف ارائه شده توسط کنفدراسیون بین المللی مامایی است. با این حال، به نظر می رسد که رویکرد عملی ارائه ی خدمات و مدل ارائه ی مراقبتهای مامایی در ایران با کشورهای پیشرو در امر بارداری و زایمان طبیعی تفاوت چشمگیری دارد. در واقع علی رغم توجه جهانی به نقش کلیدی ماما و تاکید بر سرمایه گذاری بر روی این حرفه، استفاده از ظرفیت های این حرفه در ایران تا حد زیادی مغفول مانده است. این در حالی است که ایران، رتبه ی دوم زایمان سزارین را در دنیا دارد (۵). اگر چه مامایی در ایران به عنوان یک حرفه ی مستقل تعریف شده است، با این وجود هیچ مکانیسم حرفه ای برای استقلال آن فراهم نشده است، چنانچه در سیستم مراقبتهای بهداشتی ایران، مراقبتهای دوران بارداری با رویکرد زیست پزشکی مدیریت می شود، اگرچه در این سیستم در اغلب موارد ارائه دهنده ی مراقبت به مادر باردار ماما است، با این وجود مادر باردار باید در طی بارداری چندین مرتبه توسط پزشک مرکز نیز ویزیت شود و متخصصین زنان مسوول اصلی در سراسر طول بارداری، زایمان و پس از زایمان هستند. همچنین، ماماهای ارائه دهنده ی مراقبتهای دوران بارداری، در پروسه زایمان و اولین ویزیت پس از زایمان دخالتی ندارند (۷). از سوی دیگر ماماهای ارائه دهنده ی مراقبتهای دوران بارداری در مراکز سلامت خدمات آنکالی ارائه نمی دهند، لذا در صورتی که برای مادر باردار مساله ای خارج از ساعات اداری (۸ تا ۲ بعد از ظهر) یا در روزهای تعطیلی رخ دهد باید به مراکز دیگر از جمله بیمارستان مراجعه کنند. زایمان فقط در بیمارستان ها و در مناطق روستایی در تسهیلات زایمانی قانونی می باشد و بیشتر مراحل زایمان در بیمارستان ها با مسولیت و سوپروایزری متخصصین زنان انجام می شود (۱۳). تحقیقات نشان داده اند که استانداردهای خدمات مامایی در ایران، نیازمند بازنگری جدی جهت ارائه ی خدمات با کیفیت مطلوب و با مرکزیت زن هستند. در جامعه ی شهرنشین، نگرش مثبتی نسبت به ارائه ی خدمات مامایی در سیستم بهداشتی دولتی وجود ندارد و زنان باردار ترجیح می دهند خدمات مورد نیاز خود را از مراکز خصوصی دریافت کنند (۱).

از جمله موانع اثربخشی خدمات مامایی ارائه شده در مراکز سلامت دولتی می توان به فقدان ارائه ی مراقبتهای مستمر طی بارداری، حین و بعد از زایمان و مشکلات موجود در نقش دانشگاه ها در آموزش ماماهای توانمند و ماهر اشاره کرد (۱). در مطالعه ای در ایران در خصوص 'نقش آموزش مامایی به عنوان یک چالش مهم در برنامه ی سلامت مادران'، شرکت کنندگان در پژوهش، ضعف برنامه آموزشی را به عنوان چالش اصلی عنوان کردند و ذیل آن به محیط آموزشی نامناسب، عدم کسب مهارتهای عملی، عدم وجود منابع آموزشی مناسب و روشهای نامناسب آموزشی اشاره کردند. مشکلات موجود در آموزش مامایی می تواند سبب کاهش مهارتهای تئوری و بالینی فارغ التحصیلان مامایی شود که نمود آن حضور کم رنگ و غیرموثر ماماها در عرصه ی سلامت جامعه خواهد بود. بنابراین به نظر می رسد که استفاده از روشهای سنتی آموزش نمی تواند نیازهای آموزشی دانشجویان مامایی را تامین کند. اهمیت آموزش نیروی انسانی مجرب در مامایی یک ضرورت جدی و حیاتی است و باید زمینه های پیوند اطلاعات نظری به واقعیت های بالینی به خوبی فراهم شود (۴). کیفیت آموزش مامایی نقطه‌ی ثقل در دیدگاه مراقبتهای زن محور است، لذا توانمند سازی دانشجویان مامایی برای ورود به این عرصه از ضروریات امر می باشد (۱۴).

حال با توجه به اهمیت مراقبتهای مستمر زن محور، نقش کلیدی ماما در این نوع از مراقبت و لزوم توانمندسازی ماماها در طی دوره ی آموزشی، به نظر می رسد راهکاری همچون شناخت فلسفه ی مراقبتهای مستمر زن محور توسط دانشجویان، طراحی یک طرح تلفیقی یادگیری و یاددهی، ارائه مراقبت مستمر توسط دانشجویان با پشتیبانی افراد حرفه ای برای موفقیت و اثربخشی مراقبتهای مستمر به شدت الزامی است (۱۵). در واقع دانشجویان مامایی در موقعیت منحصر به فردی برای تسهیل ارائه ی خدمات مامایی با محوریت زنان قرار گرفته اند. تمرکز بر روی آموزش دانشجویان مامایی به شیوه ی مراقبت زن محور و مبتنی بر شواهد، سبب افزایش پتانسیل دانشجوی مامایی برای عمل به عنوان یک حامی به خصوص در حین زایمان خواهد شد، چرا که در چنین شرایطی دانشجویان از یک طرف با شرایط و محیط زایمان آشنایی داشته و از سوی دیگر از نیازهای فردی زنان، خواسته ها و ترجیحات آنها آگاه بوده و می توانند نقش موثری به عنوان یک حامی ایفا کنند (۸). از طرفی، کاربرد مدل های آموزشی که دانشجویان مامایی را در بطن

مراقبتهای بالینی درگیر کند می تواند در درک فلسفه و شکل گیری فرهنگ حرفه ای آنها نقش بسزایی داشته باشد (۱۶). دانشجویانی که تجربه ی ارائه ی مراقبت مستمر را در دوران دانشجویی خود دارند، بیشتر به مشارکت در این کار بعد از فارغ التحصیلی تمایل دارند (۱۷). قرار گرفتن دانشجویان در کنار مربی ای که خود فلسفه ی مراقبت مستمر را درک نموده و در عین حال منبع موثقی برای حمایت از دانشجویان برای حضور موثرتر در این مدل است می تواند منافع بسیاری برای مادران و آموزش مامایی داشته باشد (۱۸, ۱۹). در این شیوه ترکیبی یاددهی و یادگیری، این فرصت برای دانشجو فراهم می شود که بتواند همزمان آموخته ها و تجربیات بالینی خود را سازمان دهی کنند (۱۵). حمایت دانشجویان برای ادغام دانش تئوری با یادگیری بالینی در بستر درک باورهای فلسفی در خصوص چرایی ارائه خدمات زن محور می تواند انگیزه دانشجویان را افزایش دهد (۱۶, ۲۰, ۲۱).

استرالیا یکی از کشورهای پیشرو در حوزه مامایی و مراقبت از مادران است، شورای پرستاری و مامایی استرالیا (Australian Nursing & Midwifery Accreditation Council (ANMAC)) مقرر کرده است که دانشجویان مامایی قبل از فارغ التحصیلی باید حداقل ۱۰ تجربه ی مراقبت مستمر ((Continuity of Midwifery Care (CMC)) را تحت سرپرستی مستقیم یک مامای حرفه ای در طول دوره ی آموزشی خود داشته باشند (۸). در پژوهش های انجام شده در خصوص نتایج مراقبت مستمر مامایی ارائه شده توسط دانشجویان مامایی در استرالیا گزارش شده است که بیشتر زنان رضایت زیادی از این ارتباط داشتند و اذعان داشتند که میزان رضایت و همچنین احترامی که دریافت کردند بیش از مقدار مورد انتظارشان بوده است. مبرهن است که ارائه ی مراقبت در بطن یک ارتباط صحیح و عمیق بین ارائه دهنده خدمت و مادر باردار، اساس و شالوده ی مراقبت مستمر مامایی است و مزایای این مراقبت عمدتا تحت تاثیر کیفیت رابطه‌ی زن باردار با دانشجو می باشد (۲۲). تحقیقات نشان داده اند که زنان برای این ارتباط عمیق، معنی دار و موثر خود ارزش زیادی قائل هستند و در چنین ارتباطی، دانشجویان نیز از مزایای این تداوم ارتباط بهره مند شده و چه بسا تمایل آنها برای ارائه ی خدمات فراتر از حداقل های تعیین شده افزایش می یابد (۸). در مطالعاتی که در خصوص تجربه ی زنان از ارائه ی مراقبتهای مستمر توسط دانشجویان مامایی انجام شده است دو تم اصلی تحت عناوین ارتباط و حضور گزارش شده است. در واقع زنان خواستار آن هستند که در مرکز مراقبت ها قرار داشته باشند، به گونه ای که ارائه دهنده ی خدمات که با داستان آنها آشنایی داشته و از نیازهای آنها آگاه است از نظر عاطفی و فیزیکی برای آنها حاضر و در دسترس باشد. این گروه از زنان، تجربه ی رابطه خود را با دانشجوی مامایی کاملا واقعی و شخصی گزارش کردند (۲۳-۲۶).

مشخص شده است که برقراری ارتباط بین مادر باردار و دانشجو و همچنین حضور دانشجوی مامایی ارائه دهنده مراقبت مستمر در سیر لیبر و زایمان، دو عامل اصلی و تعیین کننده از نظر ارزشمندی این مدل مراقبتی برای مادران باردار است. البته میزان زمانی که دانشجو صرف می کند و تعداد دفعاتی که طی بارداری و پس از زایمان با مادر ارتباط برقرار می کند نیز از نکات کلیدی در ساختن یک ارتباط معنی دار است (۸). در مطالعه ای نشان داده شده است که در زنان دریافت کننده ی مراقبتهای مستمر توسط دانشجویان مامایی، مصرف تنباکو بعد از هفته ۲۰ بارداری کمتر بوده، زایمان واژینال خود به خودی بیشتر، زایمان سزارین، پارگی درجه سه و چهار و همچنین اپی زیاتومی کمتر بوده است (۲۷).

با توجه به تمام بدنه ی دانشی که تاکنون وجود دارد به نظر می رسد در مدل مراقبت مامایی مستمر، بازگشت تامل برانگیزی به سوی فلسفه ی بنیادین مامایی که همانا 'همراه زن بودن' است صورت می گیرد. برای چنین رویکردی باید ماماها با کسب دانش، نگرش و مهارتهای ویژه، توانایی مشارکت حرفه ای موثر در ارائه ی مراقبتهای مستمر با کیفیت بالا قبل از زایمان تا شش هفته پس از زایمان را داشته باشند. مهارت همراهی زن برای زایمان طبیعی با کمترین مداخلات پزشکی نیازمند تجهیز ماماها به دانش به روز و مبتنی بر شواهد و دارا بودن مهارتهایی چون مهارت استدلال و قضاوت بالینی، قدرت تصمیم گیری و مسئولیت پذیری، توانایی همکاری و اعتماد است (۲۸).

با توجه به اهمیت و ضرورت سلامت مادران و نوزادان و همچنین توسعه ی حرفه ی مامایی در ایران بر اساس نیازهای جامعه، طراحی مدل های مراقبت مامایی همسو با شواهد علمی و متناسب با فرهنگ، عقاید، قوم شناسی و دانش محلی ایرانی برای ارتقای سلامت مادران، نوزادان، خانواده ها و در نهایت مردم ایران مورد نیاز است. لذا با توجه به خلا اطلاعاتی موجود در دنیا در زمینه انجام مطالعات مداخله‌ای برای تعیین اثربخشی ارائه ی مراقبت های مستمر توسط دانشجویان مامایی (ما مطالعه‌ای پیدا نکردیم)، فقدان ارائه ی مراقبت های مستمر در روند مادر شدن زنان کشورمان، کمبود قابل توجه مطالعه در این خصوص در کشورمان (ما تنها یک مطالعه ی کیفی در زمینه ارائه ی مراقبت های مستمر توسط ماماهای خصوصی به مادران یافتیم)، سیاست های اخیر جمعیتی دال بر فرزند آوری و لزوم مدیریت بهینه ی منابع انسانی در وضعیت اقتصادی کنونی کشورمان، پژوهش حاضر با هدف اجرا و ارزیابی ارائه ی مدل مراقبت مستمر توسط دانشجویان مامایی در دوران بارداری، حین و بعد از زایمان: یک مطالعه ی ترکیبی با مدل تجربی تو در تو طراحی شده است. امید است این پژوهش بتواند گامی هرچند کوچک در راستای اعتلای سلامت مادران و جایگاه مامایی در کشورمان بردارد.

مروری بر متون

۱. Tickle و همکاران (۲۰۲۱) طی مطالعه‌ای به بررسی پیامدهای بالینی در زنان باردار دریافت کننده ی مراقبتهای مستمر توسط دانشجویان مامایی در کشور استرالیا پرداختند. دانشجویان مامایی در استرالیا قبل از پایان دوره آموزشی خود موظف به ارائه ی حداقل ۱۰ مراقبت مستمر به زنان باردار هستند، تمام اطلاعات مربوط به این مراقبت ها و پیامدهای بالینی آنها در لاگ بوک دانشجویان مامایی ثبت می شود، لذا این مطالعه با رویکرد کوهورت گذشته نگر با استفاده از داده های ثبت شده در لاگ بوک دانشجویان و همچنین داده های ثبت شده در سیستم National Core Maternity و Queensland Perinatal Data انجام شد. نتایج این مطالعه نشان داد که در گروه دریافت کننده ی مراقبت مستمر توسط دانشجویان مامایی کاهش معنی داری در میزان مصرف تنباکو بعد از هفته ۲۰ بارداری، القای لیبر، میزان اپی زیاتومی و پارگی های درجه سه و چهار و استفاده از روشهای بی دردی دارویی و افزایش معنی داری در فراوانی زایمان واژینال خود به خودی وجود داشت. از نظر فراوانی زایمان با ابزار، زایمان واژینال بعد از سزارین قبلی، آپگار دقیقه پنجم و وزن هنگام تولد تفاوت معنی داری مشاهده نشد. پژوهشگران به این نتیجه رسیدند که پیامدهای بالینی در زنان دریافت کننده ی مراقبتهای مستمر توسط دانشجویان مامایی برابر یا بهتر از مراقبتهای روتین است و لذا باید این مدل مراقبت از اوایل بارداری به مادران باردار پیشنهاد شود. این محققین معتقدند نتایج پژوهش های مشابه که بتواند پتانسیل دانشجویان مامایی را برای اثرگذاری مثبت در نتایج بالینی زنان تصدیق کند، می تواند در ترغیب سیستم های بهداشتی برای ادغام این مراقبت ها در مراقبتهای زنان باردار نقش داشته باشند (۲۷).

این مطالعه به صورت کوهورت گذشته نگر انجام شده است، در پژوهش حاضر مداخله در قالب کارآزمایی بالینی تصادفی سازی شده انجام خواهد شد. از نحوه ارائه ی مداخله (مراقبت مستمر توسط دانشجویان مامایی) و همچنین پیامدهای بالینی مورد سنجش در این مقاله در طراحی پژوهش حاضر استفاده شده است.

۲. Tickle و همکاران (۲۰۲۰) در مطالعه خود به گزارش تجربه ی زنان از میزان رضایت و دریافت مراقبت احترام آمیز طی مراقبتهای مستمر دوران بارداری، زایمان و پس از زایمان توسط دانشجویان مامایی در استرالیا پرداختند. در این مطالعه توصیفی کوهورت گذشته نگر، تعداد ۸۸۶ زن که در طی ۱۲ ماه اخیر تحت مراقبت مستمر دانشجویان مامایی قرار گرفته بودند برای شرکت در یک پژوهش آنلاین دعوت شدند. در این پژوهش از دو پرسشنامه کوتاه جهت ارزیابی میزان

رضایت و برخورداری از مراقبت احترام آمیز استفاده شد. میزان پاسخ گویی افراد دعوت شده به پژوهش ۵۷٪ (۵۰۱ نفر) بود. به طور متوسط دانشجویان شش ویزیت دوران بارداری و شش تماس پس از زایمان با مادران داشتند. اغلب (۹۳٪) دانشجویان در سیر لیبر و زایمان حضور داشتند. اکثر زنان رضایت کلی خود از مراقبت دریافت شده از جانب دانشجو را 'بهتر از آنچه انتظار داشتند' ارزیابی کردند. بین تعداد ملاقاتهای قبل از زایمان و تماسهای پس از زایمان با دو متغیر رضایتمندی و مراقبت احترام آمیز، رابطه مثبت معنی‌داری وجود داشت. هنگامی که دانشجویان مامایی در سیر لیبر و زایمان حضور داشتند، زنان به طور معنی داری احساس رضایت بیشتری داشتند. در نهایت، محققین در این پژوهش اذعان داشتند که زنان برای رابطه ی مستمر خود با دانشجویان مامایی در طی بارداری، لیبر و زایمان ارزش قائل هستند و لذا برنامه های آموزشی قبل از فارغ التحصیلی همچنان باید به مراقبتهای ارتباط محور با زنان توجه کنند و استانداردهای ملی نیز باید از ادغام این مراقبتهای مستمر و موثر در برنامه های کشوری حمایت کند (۲۲).

از این مطالعه در نگارش بیان مساله، اهمیت پژوهش، اهداف پژوهش و همچنین طراحی فرم های ثبت گزارش مراقبتها توسط دانشجویان استفاده شده است.

۳. Baird و همکاران (۲۰۲۱) مطالعه ای کیفی با هدف 'تبیین تجربیات، دیدگاه ها و برنامه های دانشجویان مامایی مشارکت کننده در یک برنامه ی بالینی گروهی (Midwifery group practice (MGP)' انجام دادند. در هر یک از گروههای ارائه دهنده ی مراقبت مستمر، چهار دانشجو به مدت شش ماه، مراقبت مستمر را برای مادران (چهار تا پنج مادر در هر ماه) ارائه دادند. جمع آوری داده ها با روش بحث متمرکز گروهی در چهار گروه دو تا هفت نفره با مشارکت ۱۵ دانشجوی سال سوم کارشناسی مامایی انجام شد. پژوهشگران از روش مصاحبه ی نیمه ساختاریافته برای جمع آوری داده ها استفاده کردند. تجزیه و تحلیل داده ها حاکی از آن بود که دانشجویان این دوره را برجسته ترین تجربه ی دوران تحصیل خود می دانستند و اذعان داشتند که در این دوره آنها یاد گرفتند که 'چگونه ماما باشند'. در واقع گنجاندن مراقبت مامایی مستمر در دوره آموزشی آنها، به عنوان یک تجربه ی یادگیری ارزشمند توسط دانشجویان درک شده بود. در این پژوهش دانشجویان برای پرسپتورهای (Perceptor) فکور، مهربان و حامی خود که آنها را در ارائه ی مراقبت ها به مادران حمایت کرده بودند، ارزش زیادی قائل بودند. با این وجود دانشجویان معتقد بودند که مهارتهای توسعه یافته ی آنها در حمایت همه جانبه از زنان و نقش آنها به عنوان تسهیل گر زایمان طبیعی، هنگام بازگشت آنها به محیط های زایمانی صرفا وظیفه گرا به طور کامل مورد استفاده قرار نمی گیرد (۲۹).

۴. Carter و همکاران (۲۰۲۱) مطالعه ای با هدف بررسی دیدگاه دانشجویان مامایی در رابطه با آمادگی و انگیزه ایشان برای کار در سیستم مراقبت مامایی مستمر انجام دادند. در این مطالعه از رویکرد پراگماتیسم استفاده شد. شرکت کنندگان در پژوهش را دانشجویان مامایی سال آخر یکی از دانشگاه های استرالیا تشکیل دادند. داده های کمی با استفاده از پرسشنامه محقق ساخته (Midwifery Student Evaluation of Practice (MidSTEP و داده های کیفی با استفاده از سه سوال باز تشریحی در خصوص این که چگونه تجارب بالینی دانشجویان بر یادگیری و توسعه مهارتهای آنها تاثیر داشته جمع آوری شد. برای تجزیه و تحلیل داده های کمی از آمار توصیفی و برای داده های کیفی از تحلیل محتوا استفاده شد. نتایج این پژوهش نشان داد که قرار گرفتن در سیستم ارائه ی مراقبتهای مستمر مامایی تأثیر زیادی بر یادگیری دانشجویان داشت و آنها را قادر می ساخت تا ضمن افزایش اعتماد به نفس و آمادگی برای ارائه ی خدمات بالینی، مراقبتهای مامایی زن محور را نیز ارائه دهند. اکثر شرکت کنندگان این انگیزه را داشتند که بعد از فارغ التحصیلی در سیستم مراقبتهای مستمر مامایی مشغول به کار شوند. اقلیت کوچکی از شرکت کنندگان احساس آمادگی برای کار در سیستم مراقبتهای مستمر مامایی را نداشتند و این امر را به تعهدات خانوادگی خود، احساس نیاز به تجربه بیشتر یا فرهنگ

غیرحمایتی موجود در محیط کار نسبت دادند. در نهایت این محققین نتیجه گرفتند که تجربه ی ارائه مراقبتهای مستمر مامایی از نظر دانشجویان مامایی بسیار ارزشمند است و بر اعتماد به نفس، آمادگی و انگیزه آنها برای ارائه ی خدمات بالینی تأثیر مثبتی دارد و لذا لازم است استانداردهای آموزشی برای اطمینان از کیفیت، تداوم و کفایت این تجربیات در طی دوره آموزشی مورد بازنگری قرار گیرد (۳۰).

این مقاله در طراحی سوالات بخش کیفی ارزیابی تجربیات دانشجویان مامایی مورد استفاده قرار گرفت.

۵. Rildayani و همکاران (۲۰۲۰) مطالعه ای کمی با طرح کوهورت گذشته نگر با هدف بررسی تاثیر مدل مراقبت جامع مامایی با رویکرد یک دانشجو/یک زن بر پیامدهای زایمان انجام دادند. در مجموع ۳۰۰ زن باردار در این مطالعه شرکت کردند که ۱۵۰ زن باردار با دانشجویانی از سه ماهه سوم بارداری تا شش هفته پس از زایمان همراه بودند و ۱۵۰ زن باردار بدون دانشجو در کلینیک مامایی مراقبت شده بودند. داده‌های پژوهش از برگه‌های پارتوگراف و مستندات پرونده‌های پزشکی ثبت شده توسط دانشجویان به‌دست آمد. پیامدهای تولد شامل سن حاملگی (ترم/نارس)، زایمان طولانی مدت و وضعیت آسفیکسی بود. نتایج این پژوهش نشان داد که موارد آسفیکسی و زایمان طولانی در مادرانی که همراهی دانشجو را نداشتند بیشتر بود. محققین نتیجه گرفتند که همراهی دانشجویان مامایی از سه ماهه سوم بارداری تا شش هفته پس از زایمان می تواند پیامدهای زایمانی را بهبود بخشد (۳۹). از مطالعه حاضر در طراحی پیامدهای بالینی مطالعه استفاده شد.

۶. Shahinfar و همکاران (۲۰۲۱) مطالعه کیفی با هدف بررسی ادراک زنان از تجربه ی تداوم مراقبت تیم مامایی در ایران انجام دادند. کلینیک خصوصی مامایی برای ارائه ی مدل مراقبت تیمی مامایی انتخاب شد. تیم مامایی متشکل از سه مامای دارای مجوز بود که دو نفر از ماماها مطب خصوصی داشتند و مامای سوم دانشجوی دکترای مامایی بود. تداوم مراقبت برای شرکت کنندگان در تحقیق در طول بارداری، زایمان و دوره پس از زایمان ارائه گردید. اولین ملاقات زنان با تیم مامایی قبل از هفته ۲۴ بارداری بود. مراقبتهای دوران بارداری در کلینیک خصوصی مامایی انجام شد. ماماها یک روز در میان به کلینیک خصوصی مراجعه می کردند، اما ماماهای تیم مراقبت ۲۴ ساعت در ۷ روز هفته آنکال بودند. همه ی زنان با کمک یکی از ماماهای تیم ارائه دهنده ی مراقبت زایمان کردند. دو ویزیت معمول پس از زایمان برای مادر و نوزاد در شش هفته اول پس از تولد انجام شد. جهت جمع آوری داده ها، با روش نمونه‌گیری هدفمند، ۱۵ مصاحبه ی نیمه‌ساختار یافته با زنان به صورت انفرادی در کلینیک خصوصی مامایی انجام شد. مصاحبه ها به صورت دیجیتالی ضبط و کلمه به کلمه به زبان فارسی بازنویسی شد و با استفاده از تحلیل محتوای مرسوم (Conventional content analysis) مورد تجزیه و تحلیل قرار گرفت. تم های اصلی شامل توانمندسازی مادر و رضایت مادر در دوران گذار از بارداری به مادری بود. تم اول شامل دو مقوله ی بهبود خودکارآمدی در کلاس های آموزش دوران بارداری و تعامل موثر ماما و مادر بود. تم دوم از دو مقوله ی رضایت از روند بارداری، زایمان و پس از زایمان و همچنین رضایت از مادر شدن تشکیل شده است. یافته‌های این مطالعه کیفی، اثربخشی تداوم مراقبت‌های تیمی مامایی را در ارتقای توانمندی و رضایت زنان در دوران بارداری، تولد و پس از زایمان نشان داد (۲). از این مطالعه در طراحی نحوه ارائه ی مراقبت مستمر توسط دانشجویان مامایی و طراحی بخش کیفی مطالعه استفاده شد.

٤- ذینفعان نتایج طرح (بیمار/ صنعت/ جامعه/ سیاستگزاران/ .....) بصورت کامل توضیح داده شود.

سیستم سلامت، سیستم آموزش مامایی، زنان باردار، در حال زایمان و زایمان کرده، جنین و نوزادان و در کل خانواده آنها و درنهایت کل جامعه

۵- تعریف واژه های اختصاصی:

مراقبت مستمر تعریف نظری: مراقبت مستمر مامایی به مراقبت مبتنی بر ارتباط با یک ماما (با پشتیبانی یک تا سه ماما) اطلاق می شود که در طی آن مراقبت ها در قالب یک برنامه ی از پیش تعیین شده و به صورت آنکالی در سراسر دوران بارداری، لیبر و زایمان و تا شش هفته پس از زایمان ارائه می شود (۶). تعریف عملی: در این پژوهش مراقبت مستمر (اضافه بر مراقبت روتین) توسط یک دانشجوی مامایی (به پشتیبانی یک دانشجوی دیگر، دانشجوی پشتیبان در حداقل نیمی از مراقبتهای دوران بارداری مشارکت خواهد داشت) ترم شش/هفت به صورت دریافت حداقل دو بار مراقبت حضوری (هفته ی ۲۹-۲۶ و هفته ی ۳۶-۳۵ بارداری) و مشاوره های تلفنی و تصویری (حداقل چهار مشاوره تلفنی یا تصویری با فواصل ۱۰-۷ روز مابین دو مراقبت حضوری و سپس به صورت هفتگی تا زمان زایمان)، حضور در بالین زن و ارائه مراقبت طی لیبر و زایمان تا ۲ ساعت پس از زایمان، ویزیت حضوری ۲۴-۱۲ ساعت پس از زایمان در بیمارستان، دادن حداقل سه مشاوره تلفنی یا تصویری (۵-۳ روز پس از زایمان، ۱۰-۷ روز پس از زایمان و۳۰-۲۰ روز پس از زایمان) خواهد بود. تا حد امکان زایمان مادران شرکت کننده توسط دانشجوی اصلی ارائه دهنده مراقبت مستمر و یا در صورت عدم امکان حضور وی توسط دانشجوی جایگزین (تحت نظارت دانشجوی پژوهشگر اصلی یا هر فرد دیگری اعم از مربی مامایی، ماما یا رزیدنت زنان که مسئولیت زایمان را برعهده بگیرد) انجام خواهد گرفت. شماره موبایل دانشجویان جهت پاسخگویی به سوالات احتمالی غیر اورژانسی زنان (از ساعت ۸ صبح تا ۱۱ شب) و برای موارد اورژانسی و اطلاع شروع زایمان (به صورت آنکالی ۲۴ ساعته در ۷ روز هفته) در اختیار زنان قرار داده خواهد شد. مراقبت حضوری یا تلفنی/تصویری اضافی در طی دوران بارداری یا پس از زایمان نیز در صورت نیاز توسط دانشجو/دانشجویان ارائه خواهد شد. دانشجویان مامایی تعریف عملی: در این پژوهش دانشجویان ترم شش/هفت دانشگاه علوم پزشکی تبریز و در صورت نیاز دانشگاه آزاد اسلامی واحد تبریز می باشند که تمایل به شرکت در مطالعه داشته و در دوره های کارگاهی تحت آموزش اولیه با مضامین فلسفه ی مراقبتهای مامایی مستمر زن محور، مهارت های ارتباطی و جرات ورزی، تفکر انتقادی و حل مساله، مهارت تصمیم گیری، زایمان فیزیولوژیک، مامایی مدرن مبتنی بر شواهد، مروری بر مراقبتهای ادغام یافته سلامت مادران در بارداری، زایمان و پس از زایمان شرکت کرده باشند. تجربه ی زایمان تعریف عملی: تجربه ی زایمان یک تجربه ی شخصی در زندگی است که از فرایندهای فیزیولوژیک، روانی و ذهنی تشکیل شده و تحت¬تاثیر عوامل اجتماعی، محیطی، سازمانی و سیاست ها¬ قرار می¬گیرد. تجربه ی زایمان یک فرایند پیچیده و شخصی در زندگی زنان است (۴۲). تعریف عملی: برای سنجش تجربه ی زایمان از پرسشنامه ی تجربه زایمان نسخه ۲ (CEQ2.0=Childbirth Experiences Questionnaire vsersion 2.0) استفاده خواهد شد. این ابزار حاوی ۲۳ سوال است که ۵۰-۴۰ روز پس از زایمان به روش خودگزارش دهی توسط مادران تکمیل خواهد ¬شد (۴۳). افسردگی تعریف نظری: اختلال عاطفی یا خلق ناخوشایند یا از دست دادن علاقه به فعالیت های معمولی که به صورت واضح و نسبتاً مداوم رخ می دهد (۴۴). تعریف عملی: جهت بررسی افسردگی افراد در سه مقطع زمانی قبل از شروع مداخله (هفته ی ۲۹-۲۶ بارداری)، هفته ی ۳۶-۳۵ بارداری و ۵۰-۴۰ روز پس از زایمان از پرسشنامه ی افسردگی ادینبورگ (Edinburgh Postnatal Depression Scale (EPDS)) به روش خودگزارش دهی استفاده خواهد شد. این پرسشنامه برای اندازه گیری افسردگی دوران بارداری و پس از زایمان از ده سوال چهار گزینه¬ای (امتیاز هر سوال بین صفر تا ۳) و مجموع امتیاز بین صفر تا ۳۰ تشکیل شده است. حد آستانه ای برای تشخیص افسردگی در هر دو مقطع بارداری و پس از زایمان ۱۳ در نظر گرفته خواهد شد (۴۵). تجارب مراقبت های مادری تعریف نظری: تجربه ی مراقبت های مادری یک مفهوم چندبعدی است که عناصر متعددی من جمله: داشتن حق انتخاب از بین مراقبتهای موجود، تفاوت در نیازهای زنان، نحوه ی برقراری ارتباط با زنان، ارائه ی اطلاعات توسط متخصصان بهداشت و درک مراقبتهای دریافتی می توانند در شکل گیری آن نقش داشته باشد (۴۶). تعریف عملی: از پرسشنامه ی تجارب مراقبتهای مادری (Women's experiences of maternity care (EMC)) استفاده خواهد شد. این پرسشنامه در واقع از سه ابزار مستقل ولی مرتبط با یکدیگر تشکیل شده که تجربه مادران را از مراقبتهای دریافتی در سه فاز بارداری، لیبر و زایمان و پس از تولد را در قالب ۳۶ سوال (۱۲ سوال در هر فاز) در طیف لیکرت پنج امتیازی مورد بررسی قرار می دهد(۴۶). این پرسشنامه برای استفاده در جمعیت ایرانی روانسنجی نشده است، لذا از طراحان این پرسشنامه اجازه کتبی اخذ شده است و روانسنجی آن در قالب یک طرح تحقیقاتی انجام خواهد شد و پس از تایید اعتبار آن، ۵۰-۴۰ روز پس از زایمان به روش خودگزارش دهی تکمیل خواهد شد. ترس از زایمان تعریف نظری: ترس ناتوان کننده ای در ارتباط با زایمان که می تواند با عملکرد شغلی، خانوادگی، فعالیت ها و روابط اجتماعی فرد تداخل کند و در برخی موارد حتی در طبقه بندی فوبیا (Phobia) قرار گیرد (۴۷). تعریف عملی: در این مطالعه ترس از زایمان در سه مقطع زمانی (قبل از مداخله در هفته ی ۲۹-۲۶، هفته ی ۳۶-۳۵ بارداری و ۵۰-

۴۰ روز پس از زایمان) برای کلیه ی زنان به روش خودگزارش دهی بررسی خواهد شد. ترس از زایمان در بارداری با استفاده از پرسشنامه ی انتظار/تجربه ی زایمان ورژن A (W-DEQ-A) The Wijma delivery expectancy/experience questionnaire) و ترس پس از زایمان با استفاده از پرسشنامه ی انتظار/تجربه ی زایمان ورژن B ((W-DEQ-B) The Wijma delivery expectancy/experience questionnaire) ارزیابی خواهد شد. پرسشنامه ترس از زایمان ویجما توسط Wijma در سال ۱۹۹۸ با ۳۳ سوال در دو نسخه A و B طراحی شده است، پاسخ ها در طیف شش امتیازی لیکرت و در مقیاس کلی صفر تا ۱۶۵ نمره گذاری می شوند. نسخه ایرانی نیز دارای ۳۳ سوال می باشد و بر اساس منابع، حد آستانه ای مساوی یا بیشتر از ۶۶ به عنوان معیار ترس شدید و بیشتر یا مساوی ۸۵ به عنوان ترس بسیار شدید در نظر گرفته خواهد شد (۴۸, ۴۹). پرسشنامه ترس حین زایمان (DFS) یک پرسشنامه خودارزیابی متشکل از ۱۰ سوال است که بر اساس مقیاس۱۰ امتیازی لیکرت نمره گذاری می شوند، دامنه امتیاز ممکن از ۱۰ تا ۱۰۰ متغیر است که با جمع بندی کل نمرات پرسشنامه محاسبه می شود. نمرات بالاتر نشان دهنده ی ترس از زایمان بیشتر است . کنترل و حمایت تعریف نظری: مفهوم کنترل در حین زایمان اشاره به جنبه های مختلفی از جمله کنترل بر احساسات و رفتار خود، کنترل واکنش ها به درد و انقباضات، کنترل دریافت اطلاعات، کنترل بر اقدامات پرسنل و غیره دارد که توسط ابزارهای مختلفی از جوانب متفاوتی مورد بررسی قرار می گیرند. حمایت عموما به دریافت حمایت از جانب پرسنل اشارت دارد و می تواند مواردی چون دریافت اطلاعات و حمایت عاطفی را در برگیرد (۵۰). تعریف عملی: پرسشنامه کنترل و حمایت در زایمان ((Support and Control in Birth (SCIB)، ۱۲-۲۴ ساعت پس از زایمان به روش خودگزارش دهی تکمیل خواهد شد. این پرسشنامه حاوی ۳۳ گویه در طیف لیکرت پنج امتیازی در زیرمقیاس¬های کنترل داخلی، کنترل خارجی و حمایت می¬باشد. نمرات بالاتر نشان دهنده ی کنترل و حمایت بیشتر است (۵۰). نسخه فارسی این پرسشنامه دارای ۳۱ سوال است و مقاله ی مربوط به روانسنجی آن زیر چاپ می باشد. رضایت از زایمان تعریف نظری: رضایت از زایمان یک مفهوم چند بعدی است که عوامل مختلفی چون واقعیت رویدادها، مراقبتهای دریافتی، ترجیحات شخصی، ارزش ها و انتظارات در شکل گیری آن نقش دارند. در مقوله ی رضایت زنان از مراقبت‌های حین زایمان، در نظر گرفتن مسائلی چون درد و ناراحتی جسمی، تجربه ی هیجانهای منفی، رضایت، شادی، سازگاری هیجانی و مراقبتهای دریافتی از ارائه دهنگان خدمات الزامی است (۵۱). تعریف عملی: در این پژوهش، پرسشنامه ی رضایت مادری در زایمان طبیعی و سزارین (Maternal satisfaction in normal and caesarean birth (SMMS-normal birth and SMMS- caesarean birth)) (52) 12-24 ساعت پس از زایمان (قبل از ترخیص از بیمارستان) به روش خودگزارش دهی تکمیل خواهد شد. این پرسشنامه با ۴۳ سوال پس از زایمان طبیعی و ۴۲ سوال پس از سزارین در طیف لیکرت پنج امتیازی و در دامنه امتیازات ۲۱۵-۴۳ (زایمان طبیعی) و ۲۱۰-۴۲ (سزارین) رضایت مادران از زایمان را در ابعاد حمایت و مراقبت حرفه ای، دریافت اطلاعات و مشارکت در تصمیم گیری، مراقبت پس از زایمان، محیط، احترام به حریم خصوصی و برآورده شدن انتظارات مورد ارزیابی قرار می دهد. امتیاز حد آستانه ای برای رضایت از زایمان طبیعی ۱۵۰/۵ و در سزارین ۱۴۶/۵ می باشد، کسب نمرات بالاتر رضایت مندی بیشتر مادران را نشان می دهد (۵۳). خودکارآمدی شیردهی تعریف نظری: خودکارآمدی توانایی نشان دادن یک رفتار یا انجام کاری توصیف شده است (۵۴)، خودکارآمدی شیردهی با درک مادر از کفایت شیر برای نوزادش مرتبط است (۵۵). تعریف عملی: پرسشنامه ی خودکارآمدی شیردهی ((Breastfeeding self-efficacy scale (BSES) به روش خودگزارش دهی توسط مادران در روزهای ۵۰-۴۰ پس از زایمان تکمیل خواهد شد. این پرسشنامه دارای ۳۳ سوال در طیف لیکرت پنج امتیازی و در دامنه ی امتیاز ۳۳ تا ۱۶۵ است، امتیازات بالاتر خودکارامدی شیردهی بیشتری را نشان می دهد (۵۵, ۵۶). زایمان طبیعی تعریف عملی: در این تحقیق، زایمان طبیعی به صورت زایمان واژینال خود به خودی و یا با کمک ابزار (واکیوم یا فورسپس) تعریف خواهد شد. آپگار نوزادی تعریف نظری: یک روش قابل اعتماد و بسیار سریع در ارزیابی سلامت نوزاد در لحظه‌های ابتدایی تولد است که این مقیاس دارای پنج معیار است که به هر معیار بر حسب وضعیت نوزاد نمرات صفر، یک و یا دو اختصاص داده می¬شود و پس از جمع‌بندی پنج مقدار بدست آمده سلامت کلی نوزاد مشخص می شود. بنابراین نمره آپگار در یک محدوده صفر تا ۱۰ قرار می‌گیرد (۵۷) تعریف عملی: آپگار دقیقه ی اول و پنجم نوزادان از پرونده ی نوزادان استخراج خواهد شد. طول مدت بستری تا زایمان تعریف عملی: از زمان بستری مادر در بخش زایمان جهت ختم بارداری تا ساعت زایمان نوزاد بر اساس پرونده ی بستری مادران استخراج خواهد شد. خونریزی زایمانی تعریف نظری: خونریزی پس از زایمان به صورت میزان تجمعی خونریزی در حد بیش از ۱۰۰۰ میلی لیتر همراه با نشانه ها و علایم هیپوولمی تعریف می شود. هر گاه مقدار هماتوکریت بعد از زایمان کمتر از مقدار هماتوکریت در زمان پذیرش برای زایمان باشد مقدار خونریزی به این صورت محاسبه می شود: مجموع میزان محاسبه شده ی حجم افزوده شده در بارداری به اضافه ی ۵۰۰ میلی لیتر به ازای هر بار افت هماتوکریت در حد سه درصد حجمی (۵۸). تعریف عملی: تعریف عملی منطبق با تعریف نظری

است و برای برآورد خونریزی زایمانی از مقایسه ی هماتوکریت بعد از زایمان و هماتوکریت زمان پذیرش استفاده خواهد شد. هموگلوبین قبل از زایمان به محض پذیرش مادران در اتاق زایمان به صورت روتین اندازه گیری می شود و از پرونده ی مادران استخراج خواهد شد، هموگلوبین پس از زایمان طی ۲۴ ساعت پس از زایمان اندازه گیری خواهد شد. جوانی جمعیت نظام سلامت

٦- فرضیات طرح :

فرضیات طرح و سوالات طرح:

الف) فرضیات طرح مربوط به کارآزمایی بالینی

- **فرضیات مربوط به پیامدهای اولیه**

دریافت مراقبت مستمر دوران بارداری، زایمان و پس از زایمان از دانشجویان مامایی باعث:

۱. ارتقای تجربه ی زایمانی مادران می‌شود.
۲. کاهش نشانه های افسردگی پس از زایمان می‌شود.
۳. کاهش نشانه های ترس از زایمان پس از زایمان می شود.

- **فرضیات مربوط به پیامدهای ثانویه**

دریافت مراقبت مستمر دوران بارداری، زایمان و پس از زایمان توسط دانشجویان مامایی باعث

۱. کاهش ترس از زایمان در دوران بارداری می‌شود.
۲. کاهش نشانه های افسردگی در بارداری می‌شود.
۳. افزایش رضایت از زایمان می‌شود.
٤. افزایش کنترل و حمایت دریافتی حین زایمان می شود.
٥. افزایش خودکارآمدی شیردهی می‌شود.
٦. ارتقای تجارب مراقبتهای مادری می‌شود.
۷. افزایش فراوانی زایمان طبیعی می شود.
۸. کاهش طول مدت بستری تا زایمان می‌شود.
۹. افزایش آپگار دقیقه پنجم نوزاد می‌شود .

ب) سوالات طرح

الف) سوالات بخش کیفی:

- تجارب و دیدگاه زنان از دریافت مراقبتهای مستمر ارائه شده توسط دانشجویان مامایی در دوران بارداری، زایمان و پس از زایمان چگونه است؟
- دیدگاه دانشجویان از تجربه ی ارائه ی مراقبتهای مستمر مامایی به زنان باردار چگونه است؟

ج) سوال بخش ترکیبی:

- اجرای مدل مراقبت مستمر توسط دانشجویان مامایی در دوران بارداری، زایمان و پس از زایمان بر درک زنان از تجارب زایمانی و پیامدهای مادر و نوزادی چه تأثیری دارد؟

۷- اهداف طرح (با توجه به مقدمه و بصورتی که قابل ارزیابی و اندازه گیری باشند، مشخص شود.)

الف) هدف کلی طرح (اصولاً در برگیرنده کل عنوان طرح است):

اجرا و ارزیابی مدل مراقبت مستمر توسط دانشجویان مامایی در دوران بارداری، حین و بعد از زایمان

ب) اهداف اختصاصی طرح :

مقایسه ی میانگین نمره ی تجربه ی زایمان (۵۰-۴۰ روز پس از زایمان) در بین دو گروه با و بدون دریافت مراقبت مستمر توسط دانشجویان مامایی

مقایسه ی میانگین نمره ی ترس از زایمان (۵۰-۴۰ روز پس از زایمان) در بین دو گروه با و بدون دریافت مراقبت مستمر توسط دانشجویان

مقایسه میانگین نمره ی افسردگی پس از زایمان در بین دو گروه با و بدون دریافت مراقبت مستمر توسط دانشجویان مامایی

۸- روش اجرا (جمعیت هدف، معیارهای ورود و خروج، توصیف دقیق گروههای مورد مطالعه ،حجم نمونه و روش نمونه گیری / گردآوری، روش تحلیل داده ها) :

این پژوهش یک مطالعه ی ترکیبی با مدل تجربی تو در تو می باشد که در آن پژوهشگر فرایند مداخله را علاوه بر نتایج کمی به صورت کیفی هم بررسی می کند و با تلفیق داده‌های حاصل از بخش کیفی و کمی یافته‌های غنی‌تری بدست خواهد آمد. بخش کمی پژوهش حاضر از نوع کارآزمایی بالینی تصادفی می باشد که با اهداف اولیه تعیین تاثیر اجرای مدل مراقبت مستمر توسط دانشجویان مامایی در دوران بارداری، حین و پس از زایمان بر تجربه ی زایمان، ترس از زایمان و افسردگی پس از زایمان در زنان مراجعه کننده به مراکز سلامت شهر تبریز انجام خواهد شد. بخش کیفی نیز با هدف اولیه ی تبیین تجارب و دیدگاه زنان از اجرای مدل مراقبت مستمر توسط دانشجویان مامایی بر تجربه ی بارداری، زایمان و پس از زایمان آنها انجام خواهد شد. تبیین تجارب و دیدگاه دانشجویان مامایی از اجرای مدل مراقبتی مستمر در دوران بارداری، حین و پس از زایمان نیز به عنوان هدف ثانویه بخش کیفی انجام خواهد شد.

بخش کمی

بخش کمی مطالعه از نوع کارآزمایی بالینی تصادفی کنترل شده می باشد. پس از اخذ کد اخلاق و ثبت کارآزمایی در مرکز ثبت کارآزمایی های بالینی در ایران، نمونه گیری انجام خواهد شد. دانشجویان ارائه دهنده مراقبت مستمر، همه ی دانشجویان ترم شش/هفت دانشگاه علوم پزشکی تبریز (و در صورت نیاز دانشگاه آزاد اسلامی واحد تبریز) راضی به شرکت در پژوهش می باشند.

پس از برگزاری جلسه توجیهی و ارائه ی توضیحات مشروحی در خصوص طرح پژوهشی، اهداف و نحوه اجرای طرح، از دانشجویان داوطلب ورود به طرح رضایت نامه کتبی اخذ خواهد شد. با مدرسین دانشجویان شرکت کننده در پژوهش هم جهت همکاری در فراهم سازی ارائه ی مراقبت مستمر توسط دانشجویان، از جمله فراهم سازی امکان حضور در بخش لیبر و زایمان (حتی در صورت نیاز در زمان داشتن کارآموزی یا کارورزی در بخش دیگر) هماهنگی های لازم بعمل خواهد آمد.

جامعه ی مورد پژوهش، زنان باردار تحت پوشش مراکز سلامت منتخب شهر تبریز (از مناطق مختلف شهر) می باشند. مراکز سلامتی که بیشترین جمعیت زنان باردار را تحت پوشش دارند از مناطق مختلف شهر انتخاب خواهند شد و سپس از هر منطقه به نسبت جمعیت زنان باردار تحت پوشش تعداد نمونه به صورت نسبتی مشخص خواهد شد. شایان ذکر است که چون در جامعه ی ما افراد مرفه عموما از خدمات مراکز سلامت برای مراقبتهای بارداری و زایمان استفاده نمی کنند و تمایلی به زایمان در بیمارستان های دولتی و تامین اجتماعی ندارند، لذا این مساله جزو محدودیت های طرح ما می باشد. زنان مورد بررسی، زنان باردار تحت پوشش مراکز سلامت، با سن بارداری ۲۶ تا ۲۹ هفته بارداری، بدون سابقه زایمان یا با سابقه حداکثر دو زایمان واژینال بدون سابقه ی سزارین، فاقد بیماری های زمینه ای شناخته شده، فاقد سابقه ی بارداری پرخطر و عوارض شناخته شده در بارداری کنونی بوده که تمایل به زایمان واژینال در یکی از زایشگاههای وابسته به دانشگاه علوم پزشکی یا تامین اجتماعی شهر تبریز دارند. این افراد با استفاده از سامانه سیب شناسایی خواهند شد، سپس طی تماس تلفنی و بررسی برخی دیگر از شرایط ورود به مطالعه و توضیح مختصر اهداف و روش مطالعه، افراد بالقوه حائز شرایط جهت شرکت در مطالعه، در روز معین به مرکز تحت پوشش دعوت خواهند شد. در روز مراجعه به مرکز، بعد از ارائه ی توضیحات کامل در خصوص مطالعه، اهداف و نحوه اجرای مطالعه و شرایط ورود و خروج از مطالعه، از افراد داوطلب برای شرکت در مطالعه فرم رضایت نامه آگاهانه کتبی اخذ شده و در خصوص آزادی خروج از مطالعه در هر زمانی از پژوهش به آنها اطمینان مجدد داده خواهد شد. افراد پس از تکمیل پرسشنامه های مربوط به مشخصات پایه ای از جمله فرم مشخصات دموگرافیک و مامایی، پرسشنامه های ترس از زایمان، افسردگی و بخش اول تجارب مراقبتهای مادری (دوران بارداری) با روش تخصیص تصادفی بلوکه بندی طبقه بندی شده (بر اساس نولی پار یا مولتی پار بودن) با اندازه بلوک های ۴ و ۶ تایی با نسبت تخصیص ۱:۱ در گروه ها قرار خواهند گرفت. توالی تخصیص زنان به دانشجویان هم بصورت تصادفی ساده تعیین خواهد شد و به هر دانشجوی مامایی (به عنوان مراقب اصلی) دو مادر باردار اختصاص داده خواهد شد و در ضمن هر دانشجو، پشتیبان دو مادر باردار دانشجوی دیگر خواهد بود. تعیین توالی تخصیص زنان باردار و دانشجویان توسط فرد غیر درگیر در نمونه گیری تعیین خواهد شد. تمام مراحل انتخاب نمونه ها، اخذ رضایت آگاهانه، جمع آوری داده های پایه ای توسط دانشجوی پژوهشگر اصلی (صاحب پایان نامه) انجام خواهد شد. جهت پنهان سازی تخصیص از روش مرکزی (central) استفاده خواهد شد. به این صورت که پس از اخذ رضایت نامه آگاهانه و تکمیل مشخصات پایه‌ای، دانشجوی پژوهشگر اصلی تعداد پاریته، نام و شماره موبایل زنان باردار را به فرد غیردرگیر در نمونه گیری و جمع آوری داده‌ها از طریق پیامک ارسال خواهد نمود و وی با در نظر گرفتن گروه قرارگیری فرد و دانشجوی ارائه دهنده مراقبت از وی را با توجه به توالی تخصیص از قبل مشخص شده مشخص خواهد نمود.

ارائه دهندگان اصلی مراقبت مستمر مادران (از هفته ۲۹-۲۶ بارداری تا شش هفته پس از زایمان)، دانشجویان ترم شش/هفت مامایی دانشگاه علوم پزشکی تبریز (و در صورت نیاز دانشگاه آزاد تبریز) می‌باشند که فرم رضایت آگاهانه ی کتبی جهت شرکت در مطالعه را امضا نموده و و طی چهارجلسه ی کارگاهی چهار ساعته، آموزشهای ویژه از جمله فلسفه ی مراقبتهای مامایی مستمر زن محور، مهارت های ارتباطی و جرات ورزی، تفکر انتقادی و حل مساله، مهارت تصمیم گیری، زایمان فیزیولوژیک، مامایی مدرن مبتنی بر شواهد، مروری بر مراقبتهای ادغام یافته سلامت مادران در بارداری، زایمان و پس از زایمان را قبل از ورود به مطالعه دریافت نموده باشند. در تمام طول مدت پژوهش با تشکیل گروه واتس اپ، دانشجویان با یکدیگر و با دانشجوی پژوهشگر اصلی در ارتباط خواهند بود و تحت منتورشیپی دانشجوی پژوهشگر اصلی (با ۱۵ سال سابقه در آموزش مامایی و عضو هسته آموزشی زایمان فیزیولوژیک)

قرار خواهند گرفت. تمام مطالب آموزشی مطرح شده در دوره کارگاهی، محتوای مربوط به جلسات مراقبت و همچنین سوالات مطرح شده توسط دانشجویان در سیر ارائه ی مراقبت به مادران در گروه تمرین و تکرار خواهد شد. پژوهشگر اصلی بعنوان منتورشیپ دانشجویان به صورت آنکالی پاسخ گوی دانشجویان خواهد بود. همچنین فرم هایی طراحی شده است که دانشجویان پس از ارائه ی هر مراقبت به مادر، آنها را تکمیل کرده و در اختیار منتور قرار خواهد داد تا نقاط قوت و ضعف دانشجو در ارائه ی مراقبت به وی بازخورد داده شود و در صورت لزوم اقدامات ضروری برای مادر اصلاح شود. همچنین سعی خواهد شد در مراقبت های اولیه که هر دانشجو ارائه می دهد، منتور به صورت حضوری یا مجازی حضور یابد و نحوه ی ارائه ی مراقبت دانشجو را چه در زمینه ی مهارت مامایی و چه در زمینه ی مهارت ارتباطی ارزیابی کند و به وی فیدبک دهد، چنانچه این مساله مقدور نباشد با رضایت مادران ویس جلسه ی چند مراقبت اولیه هر دانشجو ضبط و در اختیار منتور قرار داده خواهد شد تا راهنمایی لازم برای ادامه ی مراقبت ها به دانشجویان داده شود.

زنان در هر دو گروه مورد مطالعه مراقبتهای استاندارد ارائه شده در مراکز سلامت را دریافت خواهند کرد. زنان گروه مداخله از زمان ورود به مطالعه تا شش هفته پس از زایمان علاوه بر دریافت مراقبت روتین، یک دفترچه ی راهنمای دوران بارداری، زایمان و پس از زایمان را که در این مطالعه طراحی خواهد شد دریافت کرده و تحت مراقبت مستمر توسط یک دانشجوی ترم شش/هفت (با پشتیبانی یک دانشجوی دیگر) قرار خواهند گرفت. مراقبت مستمر به صورت دریافت حداقل دو بار مراقبت حضوری (بین هفته ی ۲۹-۲۶ و بین هفته ی ۳۶-۳۵ بارداری) و مشاوره های تلفنی و تصویری (حداقل چهار مشاوره تلفنی یا تصویری با فواصل ۱۰-۷ روز مابین دو مراقبت حضوری و سپس به صورت هفتگی از هفته ی ۳۷ بارداری تا زمان زایمان)، حضور در بالین زن و ارائه ی مراقبت طی لیبر و زایمان تا ۲ ساعت پس از زایمان، ویزیت حضوری ۲۴-۱۲ ساعت پس از زایمان در بیمارستان، دادن حداقل سه مشاوره ی تلفنی یا تصویری (۵-۳ روز پس از زایمان، ۱۰-۷ روز پس از زایمان و۳۰-۲۰ روز پس از زایمان) خواهد بود. کتابچه راهنمایی با محوریت بوکلت مراقبت های ادغام یافته سلامت مادران و همچنین کتاب راهنمایی سازمان جهانی بهداشت برای مراقبت های بارداری و زایمان توسط پژوهشگر اصلی تهیه خواهد شد و در اختیار دانشجویان قرار داده خواهد شد تا به عنوان کتابچه ی راهنمای گام به گام مراقبت ها در هر جلسه مراقبت حضوری یا تلفنی، مراقبت ها را بر اساس آن ارائه دهند. ارائه ی مراقبت ها در هر جلسه دانشجوی اصلی و تا حد امکان پشتیبان وی در جلسات مراقبتی و مشاوره‌ای (حضوری و آنلاین) حضور خواهند داشت (دانشجوی پشتیبان در حداقل نیمی از مراقبت های دوران بارداری حضور خواهد داشت). شماره موبایل دانشجویان جهت پاسخگویی به سوالات احتمالی غیر اورژانسی زنان (از ساعت ۸ صبح تا ۱۱ شب) و برای موارد اورژانسی و اطلاع شروع زایمان (به صورت آنکالی ۲۴ ساعته در ۷ روز هفته) در اختیار زنان قرار داده خواهد شد. زنان باردار برای سوالات خود با دانشجوی اصلی و در صورت عدم دسترسی به وی با دانشجوی پشتیبان تماس خواهند گرفت. دانشجوی اصلی ارائه دهنده ی مراقبت به هر مادر بطور مرتب گزارشی از هر گونه اقدام انجام گرفته برای مادر را به دانشجوی پژوهشگر اصلی ارائه داده و بازخورد خواهد گرفت. سعی خواهد شد که یکی از دانشجویان اصلی مراقبت مستمر یا پشتیبان وی بومی باشد. تا حد امکان زایمان این گروه از مادران، تحت نظارت دانشجوی پژوهشگر اصلی یا هر فرد دیگری اعم از مربی مامایی، ماما یا رزیدنت زنان که مسئولیت زایمان را برعهده بگیرد، توسط دانشجویان ارائه دهنده مراقبت مستمر انجام خواهد گرفت. مراقبت حضوری یا آنلاین اضافی در طی دوران بارداری یا پس از زایمان نیز در صورت نیاز توسط دانشجو/دانشجویان ارائه خواهد شد.

به زنان گروه کنترل هم دفترچه ی راهنمای دوران بارداری، زایمان و پس از زایمان تدوین شده در این پژوهش در همان ابتدای ورود به مطالعه داده می شود و همچنین این گروه از مادران از شش هفته (پایان پیگیری تاثیر مداخله) تا شش ماه پس از زایمان مشاوره ی رایگان غیرحضوری توسط دانشجوی پژوهشگر اصلی را دریافت خواهند نمود. تمام داده های اصلی طی و پس از مداخله نیز توسط دانشجوی پژوهشگر اصلی جمع آوری خواهد شد (نمودار جریان فرایند پژوهش در ذیل آورده شده است).

۹- توضیحات تکمیلی اهداف اختصاصی:

-برای هر کدام از اهداف اختصاصی با روش اجرا، متغیرها و یا روش آنالیز آماری یکسان، متن و جدول مشترک آورده شود در غیر اینصورت جداگانه نوشته شود.

عنوان هدف اختصاصی شماره یک :

مقایسه ی میانگین نمره ی تجربه ی زایمان (۵۰-۴۰ روز پس از زایمان) در بین دو گروه با و بدون دریافت مراقبت مستمر توسط دانشجویان مامایی

الف) خلاصه روش اجرا هدف اختصاصی یک: (توصیف دقیق گروههای مورد مطالعه، تعداد نمونه هر گروه، نحوه انجام تحقیق یا آزمایش)):

مقایسه بین گروه مداخله (دریافت مراقبت مستمر توسط دانشجوی مامایی) و گروه کنترل (بدون مراخله)، ۵۰-۴۰ روز پس از زایمان با استفاده از پرسشنامه ی تجربه ی زایمان انجام خواهد شد. تعداد نمونه در هر گروه ۴۶ نفر خواهد بود.

جدول متغیرهای هدف اختصاصی یک (Variables): پیرو مصوبه شورای پژوهشی دانشگاه، در پروپوزال پایان نامه ها و طرح های تحقیقاتی وارد نمودن جدول متغییر ها مورد نیاز نمی باشد.

متغیر ها	نوع مقیاس	نوع متغیر	روش کنترل / ارزیابی	تعریف علمی متغیر(در صورت نیاز)

ب) توضیح کامل روش تجزیه و تحلیل آماری داده ها هدف اختصاصی یک:

برای مقایسه میانگین نمره ها درگروههای مورد مطالعه از تحلیل کواریانس با کنترل عامل طبقه بندی استفاده خواهدشد. سطح معنی داری در همه ی آزمون‌ها ($P<0.05$) در نظر گرفته شده‌است.

عنوان هدف اختصاصی شماره دو :

مقایسه ی میانگین نمره ی ترس از زایمان (۵۰-۴۰ روز پس از زایمان) در بین دو گروه با و بدون دریافت مراقبت مستمر توسط دانشجویان

الف) خلاصه روش اجرا هدف اختصاصی دو: (توصیف دقیق گروههای مورد مطالعه، تعداد نمونه هر گروه، نحوه انجام تحقیق یا آزمایش)):

مقایسه دو گروه مداخله (دریافت مراقبت مستمر توسط دانشجوی مامایی) و گروه کنترل (بدون مداخله)، ۵۰-۴۰ روز پس از زایمان با استفاده از پرسشنامه ی ترس از زایمان ویجما ورژن B انجام خواهد شد.

ب) توضیح کامل روش تجزیه و تحلیل آماری داده ها هدف اختصاصی دو:

برای مقایسه میانگین نمره ها درگروههای مورد مطالعه از تحلیل کواریانس با کنترل عوامل طبقه بندی و نمره ی پایه استفاده خواهدشد. سطح معنی داری در همه ی آزمون‌ها ($P<0.05$) در نظر گرفته شده است.

عنوان هدف اختصاصی شماره سه :

مقایسه میانگین نمره ی افسردگی پس از زایمان در بین دو گروه با و بدون دریافت مراقبت مستمر توسط دانشجویان مامایی

الف) خلاصه روش اجرا هدف اختصاصی سه: (توصیف دقیق گروههای مورد مطالعه، تعداد نمونه هر گروه، نحوه انجام تحقیق یا آزمایش)):

مقایسه ی گروه مداخله (دریافت مراقبت مستمر توسط دانشجوی مامایی) و گروه کنترل (بدون مداخله)، ۵۰-۴۰ روز پس از زایمان با استفاده از پرسشنامه افسردگی ادینبرگ انجام خواهد شد.

ب) توضیح کامل روش تجزیه و تحلیل آماری داده ها هدف اختصاصی سه:

برای مقایسه میانگین نمره ها درگروههای مورد مطالعه از تحلیل کواریانس با کنترل عوامل طبقه بندی و نمره ی پایه استفاده خواهدشد. سطح معنی داری در همه ی آزمون‌ها ($P<0.05$) در نظر گرفته شده است.

جدول متغیرهای مشترک بین اهداف (Variables): پیرو مصوبه شورای پژوهشی دانشگاه، در پروپوزال پایان نامه ها و طرح های تحقیقاتی وارد نمودن جدول متغییر ها مورد نیاز نمی باشد.

متغیر ها	نوع مقیاس	نوع متغیر	روش کنترل / ارزیابی	تعریف علمی متغیر(در صورت نیاز)

۱۰- ملاحظات اخلاقی (هر طرحی بسته به نوع مطالعه، ملاحظات خاص خود را دارد که بر اساس دستور العمل کمیته منطقه ای اخلاق بایستی مد نظر قرار گیرد- در صورت نیاز فرم رضایتنامه آگاهانه تکمیل گردد لازم به ذکر است شروع مراحل اجرایی پژوهش تنها پس از اخذ مجوز از کمیته اخلاق میسر خواهد بود)

توضیحات مجری:

ملاحظات اخلاقی بخش کمی ۱. اخذ مجوز از کمیته اخلاق دانشگاه ۲. ثبت کارآزمایی در IRCT ۳. کسب اجازه از ریاست و معاونت محترم پژوهشی دانشکده پرستاری و مامایی جهت انجام پژوهش ۴. گرفتن معرفی نامه از دانشکده پرستاری و مامایی و ارائه آن به مراکز به سلامت، بیمارستانهای دانشگاهی و تامین اجتماعی شهر تبریز ۵. معرفی پژوهشگر به مسئولین مراکز سلامت و بیمارستانها و کسب اجازه از آنها جهت انجام پژوهش ۶. معرفی پژوهشگر به کلیه واحدهای پژوهش و توضیح هدف انجام پژوهش برای آنها ۷. کلیه واحدهای پژوهش پس از اعلام رضایت خود به صورت کتبی و کسب اجازه وارد پژوهش می شوند. ۸. کلیه واحدهای پژوهش در صورت عدم تمایل به صورت آزادانه می توانند از مطالعه خارج شوند. ۹. به کلیه واحدهای پژوهش اطمینان داده می¬شود که تمام اطلاعات فردی ایشان محرمانه بوده و نیازی به نوشتن اسم نیست. ۱۰. رعایت ملاحضات فرهنگی و مذهبی مشارکت کنندگان ۱۱. به کلیه واحدهای پژوهش و مسئولین اطمینان داده می شود که در صورت تمایل نتایج پژوهش به آنان اعلام شود. ۱۲. کلیه نکات اخلاقی در استفاده از منابع علمی و مقالات مورد نظر قرار خواهد گرفت.
ملاحظات اخلاقی بخش کیفی ۱. اخذ رضایت نامه آگاهانه کتبی از مشارکت کنندگان ۲. اطمینان دادن به مشارکت‌کنندگان نسبت به اختیاری

بودن شرکت در پژوهش ۳. مختار بودن مشارکت‌کنندگان نسبت به خاتمه دادن همکاری خود در هر زمان ۴. تعیین زمان و مکان مصاحبه با موافقت مشارکت‌کنندگان ۵. اطمینان دادن به مشارکت‌کنندگان نسبت به عدم فاش شدن هویت و اطلاعاتشان ۶. انتشار اطلاعات مشارکت کنندگان با کد عددی و یا نام مستعار ۷. توضیح در مورد یادداشت برداری و استفاده از ضبط صوت و در صورت عدم تمایل ایشان، خاموش کردن ضبط صوت

راهنمای کدهای اخلاق در پژوهش را مطالعه کردم و آنها را رعایت میکنم.

Link به کدهای مربوطه اخلاق در پژوهش های علوم پزشکی

۱۱- جدول زمانی مراحل اجرا طرح (GANTT CHART)

ردیف	شرح هر یک از فعالیتهای اجرائی طرح به تفکیک	طول مدت (ماه)
۱	تعیین عنوان و نگارش پروپوزال	۳
۲	تهیه ابزار و تعیین روایی و پایایی، تهیه کتابچه آموزشی برای مادران، برگزاری کارگاه برای دانشجویان شرکت کننده در مطالعه	۳
۳	نمونه¬گیری مرحله کمی	۹
۴	نمونه¬گیری مرحله کیفی	۷
۵	وارد کردن داده¬ها به کامپیوتر و تجزیه و تحلیل آماری	۲
۶	تهیه گزارش نهایی و نگارش مقاله	۳
۷	مدت زمان کلی	۱۸

۱۲- هزینه آزمایشها و خدمات تخصصی که توسط موسسات دیگر صورت می گیرد:

موضوع آزمایشات و یا خدمات تخصصی	مرکز ارائه دهنده خدمات	تعداد کل دفعات	هزینه برای هر دفعه (ریال)	جمع (ریال)
CBC	آزمایشگاه بیمارستان های محل پژوهش	۹۶	۱۲۰,۰۰۰	۱۱,۵۲۰,۰۰۰
جمع هزینه های آزمایشات و خدمات تخصصی (ریال):				۱۱,۵۲۰,۰۰۰

۱۳- جدول هزینه های وسایل و مواد:

نام وسایل و یا موادمصرفی	نام محل تهیه	تعداد یا مقدار لازم	مبلغ واحد (ریال)	کل مبلغ (ریال)
Flash memory		۱.۰	۱,۰۰۰,۰۰۰	۱,۰۰۰,۰۰۰

کاغذ A4		۵.۰	۷۵۰,۰۰۰	۳,۷۵۰,۰۰۰
جمع کل (ریال)				۴,۷۵۰,۰۰۰

۱۴- سایر هزینه ها:

موضوع هزینه	توضیح در باره کمیت و یا کیفیت موضوع هزینه	کل مبلغ (ریال)
هزینه مسافرت		۴۵,۰۰۰,۰۰۰
هزینه تایپ، تکثیر، تقاضای patent ، ...		۱۸,۷۵۰,۰۰۰
هزینه های متفرقه (داوطلبین، ...)		۱۱۳,۶۰۰,۰۰۰
جمع هزینه (ریال):		۱۷۷,۳۵۰,۰۰۰

۱۵- جمع هزینه های طرح:

نوع	مبلغ (ریال)
پرسنلی	۷۳,۵۰۰,۰۰۰
آزمایشات و خدمات تخصصی	۱۱,۵۲۰,۰۰۰
وسایل و مواد	۴,۷۵۰,۰۰۰
سایر	۱۷۷,۳۵۰,۰۰۰
کل اعتبار طرح	۲۶۷,۱۲۰,۰۰۰
اعتبار تامین شده خارج از دانشگاه	۰
جمع کل هزینه های طرح (ریال):	۲۶۷,۱۲۰,۰۰۰

۱۶- منابع علمی (متن کامل حداقل سه منبع اصلی مرتبط با موضوع ضمیمه شود) :

منابع فارسی:

منابع انگلیسی:

Refrences 1. Firouznia R, Dargahi H, Jafari Koshki T, Khaledian Z. Challenges of Iranian Maternal Health Program from Midwives' Perspectives: A Qualitative Study. Jundishapur Journal of Health Sciences. 2019;11(3). 2. Shahinfar S, Abedi P, Najafian M, Abbaspoor Z, Mohammadi E, Alianmoghaddam N. Women's perception of continuity of team midwifery care in Iran: a qualitative content analysis. BMC pregnancy and childbirth. 2021;21(1):173. 3. Organisation WH. WHO Statement on Antenatal Care.

Geneva: WHO2011. 4. Ahmady S, Yazdi L. Exploring the midwifery training challenges in Iran from the viewpoint of faculty members and graduates of this field: Content analysis. 2016. 5. Moghasemi S, Vedadhir A, Simbar M. Models for providing midwifery care and its challenges in the context of Iran. Journal of Holistic Nursing And Midwifery. 2018;28(1):64-74. 6. Cummins A, Coddington R, Fox D, Symon A. Exploring the qualities of midwifery-led continuity of care in Australia (MiLCCA) using the quality maternal and newborn care framework. Women and birth : journal of the Australian College of Midwives. 2020;33(2):125-34. 7. Sandall J, Soltani H, Gates S, Shennan A, Devane D. Midwife-led continuity models versus other models of care for childbearing women. The Cochrane database of systematic reviews. 2016;4(4):Cd004667. 8. Tickle N, Sidebotham M, Fenwick J, Gamble J. Women's experiences of having a Bachelor of Midwifery student provide continuity of care. Women and birth : journal of the Australian College of Midwives. 2016;29(3):245-51. 9. Leap N, Sandall J, Buckland S, Huber U. Journey to confidence: women's experiences of pain in labour and relational continuity of care. Journal of midwifery & women's health. 2010;55(3):234-42. 10. van Teijlingen ER, Hundley V, Rennie AM, Graham W, Fitzmaurice A. Maternity satisfaction studies and their limitations: "What is, must still be best". Birth (Berkeley, Calif). 2003;30(2):75-82. 11. Hunter B, Berg M, Lundgren I, Olafsdóttir OA, Kirkham M. Relationships: The hidden threads in the tapestry of maternity care. Midwifery. 2008;24(2):132-7. 12. Styles C, Kearney L, George K. Implementation and upscaling of midwifery continuity of care: The experience of midwives and obstetricians. Women and birth : journal of the Australian College of Midwives. 2020;33(4):343-51. 13. Torkzahrani S. Commentary: childbirth education in iran. The Journal of perinatal education. 2008;17(3):51-4. 14. Luyben A, Barger M, Avery M, Bharj KK, O'Connell R, Fleming V, et al. Exploring global recognition of quality midwifery education: Vision or fiction? Women and birth : journal of the Australian College of Midwives. 2017;30(3):184-92. 15. Bass J, Sidebotham M, Gamble J, Fenwick J. Commencing undergraduate midwifery students' beliefs about birth and the role of the midwife. International Journal of Childbirth. 2015;5(2):83. 16. Ebert L, Tierney O, Jones D. Learning to be a midwife in the clinical environment; tasks, clinical practicum hours or midwifery relationships. Nurse education in practice. 2016;16(1):294-7. 17. Evans J, Taylor J, Browne J, Ferguson S, Atchan M, Maher P, et al. The future in their hands: Graduating student midwives' plans, job satisfaction and the desire to work in midwifery continuity of care. Women and birth : journal of the Australian College of Midwives. 2020;33(1):e59-e66. 18. Carter AG, Wilkes E, Gamble J, Sidebotham M, Creedy DK. Midwifery students′ experiences of an innovative clinical placement model embedded within midwifery continuity of care in Australia. Midwifery. 2015;31(8):765-71. 19. Cummins AM, Denney-Wilson E, Homer CSE. The mentoring experiences of new graduate midwives working in midwifery continuity of care models in Australia. Nurse education in practice. 2017;24:106-11. 20. Licqurish S, Seibold C. 'Chasing the numbers': Australian Bachelor of Midwifery students' experiences of achieving midwifery practice requirements for registration. Midwifery. 2013;29(6):661-7. 21. Carter J, Dietsch E, Sidebotham M. The impact of pre-registration education on the motivation and preparation of midwifery students to work in continuity of midwifery care: An integrative review. Nurse education in practice. 2020;48:102859. 22. Tickle N, Gamble J, Creedy DK. Women's reports of satisfaction and respect with continuity of care experiences by students: Findings from a routine, online survey. Women and birth :

journal of the Australian College of Midwives. 2021;34(6):e592-e8. 23. Aune I, Dahlberg U, Ingebrigtsen O. Relational continuity as a model of care in practical midwifery studies. British Journal of Midwifery. 2011;19(8):515-23. 24. Kelly J, West R, Gamble J, Sidebotham M, Carson V, Duffy E. 'She knows how we feel': Australian Aboriginal and Torres Strait Islander childbearing women's experience of Continuity of Care with an Australian Aboriginal and Torres Strait Islander midwifery student. Women and birth : journal of the Australian College of Midwives. 2014;27(3):157-62. 25. Browne J, Taylor J. 'It's a good thing…': Women's views on their continuity experiences with midwifery students from one Australian region. Midwifery. 2014;30(3):e108-14. 26. Dahlberg U, Aune I. The woman's birth experience---the effect of interpersonal relationships and continuity of care. Midwifery. 2013;29(4):407-15. 27. Tickle N, Gamble J, Creedy DK. Clinical outcomes for women who had continuity of care experiences with midwifery students. Women and birth : journal of the Australian College of Midwives. 2021. 28. Fahy K. What makes a midwifery model of care safe? Women and birth : journal of the Australian College of Midwives. 2012;25(1):1-3. 29. Baird K, Hastie CR, Stanton P, Gamble J. Learning to be a midwife: Midwifery students' experiences of an extended placement within a midwifery group practice. Women and birth : journal of the Australian College of Midwives. 2021. 30. Carter J, Sidebotham M, Dietsch E. Prepared and motivated to work in midwifery continuity of care? A descriptive analysis of midwifery students' perspectives. Women and birth : journal of the Australian College of Midwives. 2021. 31. Foster W, Sweet L, Graham K. Midwifery students experience of continuity of care: A mixed methods study. Midwifery. 2021;98:102966. 32. Gamble J, Sidebotham M, Gilkison A, Davis D, Sweet L. Acknowledging the primacy of continuity of care experiences in midwifery education. Women and birth : journal of the Australian College of Midwives. 2020;33(2):111-8. 33. Hainsworth N, Dowse E, Ebert L, Foureur M. 'Continuity of Care Experiences' within pre-registration midwifery education programs: A scoping review. Women and birth : journal of the Australian College of Midwives. 2021;34(6):514-30. 34. Zwedberg S, Barimani M. When student midwives are present during labour and childbirth in a peer-learning model: An interview study of parents in Sweden. Midwifery. 2022;104:103173. 35. Gamble J, Pallant J, Creedy DK. Evaluation of the Midwifery Student Evaluation of Practice (MidSTEP) tool using Rasch analysis. Nurse education today. 2022;108:105174. 36. Tickle N, Sidebotham M, Fenwick J, Gamble J. Women's experiences of having a Bachelor of Midwifery student provide continuity of care. Women and Birth. 2016;29(3):245-51. 37. Tickle N, Gamble J, Creedy DK. Feasibility of a novel framework to routinely survey women online about their continuity of care experiences with midwifery students. Nurse education in practice. 2021;55:103176. 38. Stulz V, Elmir DR, Reilly H. Evaluation of a student-led midwifery group practice: A woman's perspective. Midwifery. 2020;86:102691. 39. Rildayani R, Nurjannah N, Saputra I, Yeni CM, Usman S. The Effect of the Comprehensive Midwifery Care Model with the One Student One Client (OSOC) Approach to Birth Outcomes in North Aceh Regency. Budapest International Research and Critics Institute (BIRCI-Journal): Humanities and Social Sciences. 2020;3(3):1676-82. 40. Hildingsson I, Karlström A, Larsson B. Childbirth experience in women participating in a continuity of midwifery care project. Women and birth : journal of the Australian College of Midwives. 2021;34(3):e255-e61. 41. Newton M, Faulks F, Bailey C, Davis J, Vermeulen M, Tremayne A, et al. Continuity of care experiences: A national

cross-sectional survey exploring the views and experiences of Australian students and academics. Women and birth : journal of the Australian College of Midwives. 2021. 42. James S. Women's experiences of symptoms of posttraumatic stress disorder (PTSD) after traumatic childbirth: a review and critical appraisal. Arch Womens Ment Health. 2015;18(6):761-71. 43. Ghanbari-Homayi S, Dencker A, Fardiazar Z, Jafarabadi MA, Mohammad-Alizadeh-Charandabi S, Meedya S, et al. Validation of the Iranian version of the childbirth experience questionnaire 2.0. BMC pregnancy and childbirth. 2019;19(1):465. 44. Chorwe-Sungani G, Chipps J. A systematic review of screening instruments for depression for use in antenatal services in low resource settings. BMC psychiatry. 2017;17(1):112. 45. Levis B, Negeri Z, Sun Y, Benedetti A, Thombs BD. Accuracy of the Edinburgh Postnatal Depression Scale (EPDS) for screening to detect major depression among pregnant and postpartum women: systematic review and meta-analysis of individual participant data. BMJ (Clinical research ed). 2020;371:m4022. 46. Redshaw M, Martin CR, Savage-McGlynn E, Harrison S. Women's experiences of maternity care in England: preliminary development of a standard measure. BMC pregnancy and childbirth. 2019;19(1):167. 47. Wijma K. Why focus on 'fear of childbirth'? J Psychosom Obstet Gynaecol. 2003;24(3):141-3. 48. Mortazavi F. Validity and reliability of the Farsi version of Wijma delivery expectancy questionnaire: an exploratory and confirmatory factor analysis. Electronic physician. 2017;9(6):4606-15. 49. Nilsson C, Hessman E, Sjöblom H, Dencker A, Jangsten E, Mollberg M, et al. Definitions, measurements and prevalence of fear of childbirth: a systematic review. BMC pregnancy and childbirth. 2018;18(1):28. 50. Ford E, Ayers S, Wright DB. Measurement of maternal perceptions of support and control in birth (SCIB). Journal of women's health (2002). 2009;18(2):245-52. 51. Rudman A, El-Khouri B, Waldenström U. Women's satisfaction with intrapartum care - a pattern approach. J Adv Nurs. 2007;59(5):474-87. 52. Gungor I, Beji NK. Development and psychometric testing of the scales for measuring maternal satisfaction in normal and caesarean birth. Midwifery. 2012;28(3):348-57. 53. Pakari N, Zahrani ST, Nasiri M, Mahmoodi Z. Persian translation and psychometric testing of the scales for measuring maternal satisfaction in normal and caesarean birth. Biosciences Biotechnology Research Asia. 2016;13(1):339-46. 54. Bandura A. Social cognitive theory: an agentic perspective. Annu Rev Psychol. 2001;52:1-26. 55. Dennis CL. The breastfeeding self-efficacy scale: psychometric assessment of the short form. J Obstet Gynecol Neonatal Nurs. 2003;32(6):734-44. 56. Araban M, FALAHIYAN MF, Shahry P, Montazeri A. The Persian version of breastfeeding self-efficacy scale-short form (BSES-SF): translation and psychometric assessment. 2016. 57. Simon LV, Hashmi MF, Bragg BN. APGAR Score. StatPearls. Treasure Island (FL): StatPearls Publishing Copyright © 2022, StatPearls Publishing LLC.; 2022. 58. Cunningham FG, Leveno KJ, Bloom SL, Spong CY, Dashe JS. Williams obstetrics, 25e: Mcgraw-hill New York, NY, USA; 2018. 59. Tabatabaee A, Hasani P, Mortazavi H, Tabatabaeichehr M. Strategies to enhance rigor in qualitative research. Journal of North Khorasan University of Medical Sciences. 2013;5(3):663-70. 60. Taylor SJ, Bogdan R, DeVault M. Introduction to qualitative research methods: A guidebook and resource: John Wiley & Sons; 2015. 61. Ghaedi M, Golshani A. Content analysis method: From quantity-orientation to quality-orientation. Psychological methods and models. 2016;7(23):57-82. 62. Kiamanesh Alireza SJ. Mixed Methods Research. 2 ed2007. 63. Ghanbari-Homayi S, Fardiazar Z, Meedya S,

Mohammad-Alizadeh-Charandabi S, Asghari-Jafarabadi M, Mohammadi E, et al. Predictors of traumatic birth experience among a group of Iranian primipara women: a cross sectional study. BMC pregnancy and childbirth. 2019;19(1):182. 64. Afshari P, Tadayon M, Abedi P, Yazdizadeh S. Prevalence and related factors of postpartum depression among reproductive aged women in Ahvaz, Iran. Health Care Women Int. 2020;41(3):255-65. 65. Andaroon N, Kordi M, Kimiaei SA, Esmaeily H. The effect of individual counseling program by a midwife on fear of childbirth in primiparous women. J Educ Health Promot. 2017;6:97. 66. Walker KF, Dencker A, Thornton JG. Childbirth experience questionnaire 2: Validating its use in the United Kingdom. Eur J Obstet Gynecol Reprod Biol X. 2020;5:100097. 67. Montazeri A, Torkan B, Omidvari S. The Edinburgh Postnatal Depression Scale (EPDS): translation and validation study of the Iranian version. BMC psychiatry. 2007;7:11. 68. Wijma K, Alehagen S, Wijma B. Development of the Delivery Fear Scale. J Psychosom Obstet Gynaecol. 2002;23(2):97-107. 69. Dennis CL, Faux S. Development and psychometric testing of the Breastfeeding Self-Efficacy Scale. Res Nurs Health. 1999;22(5):399-409.

Embedded Experimental Model diagram.pdf

Tickle clinical outcome.pdf

Gamble Acknowledging.pdf

Flow Diagram.pdf

۱۷- آیا منبع دیگری (به غیر از معاونت تحقیقات و فناوری دانشگاه) در تامین هزینه طرح مشارکت خواهد داشت ؟

بله خیر

لطفاً میزان مشارکت و چگونگی آن توضیح داده شود:

محل تخصیص	نوع مرکز	زمان تخصیص	شرایط تخصیص	توضیحات مشروح در خصوص اعتبار	میزان اعتبار

۱۸- آیا این طرح در محل دیگر (دانشگاه و خارج از دانشگاه) نیز ارائه شده است؟

بله خیر

اگر بلی: نام محل : نتیجه ارائه : تصویب عدم تصویب

نوع تحقیق فوق از جهت آزمایشگاهی و نوع مطالعه بر روی حیوانات یا انسانی بودن استفاده را مشخص نمایید؟ انسانی

توضیحات اضافی مجری/استاد راهنما:

۱۹- در صورتی که طرح پایاننامه است بند زیر تکمیل و امضا شود:

این طرح پایاننامه ای در شورای گروه گروه مامایی در مورخه ۱۵/۰۸/۱۴۰۰ به تصویب رسید.

امضای مدیر گروه / معاون پژوهشی

۲۰-ناظران پیشنهادی

نام و نام خانوادگی ناظر پیشنهادی	رشته تخصصی/تحصیلی	مرتبه علمی یا میزان تحصیلات	پست الکترونیک	تلفن تماس
دکتر مهناز شهنازی	کارشناس ارشد مامایی و دکترای تخصصی فیزیولوژی	استادیار		۰۹۱۴۳۰۰۲۴۳۳
دکتر سولماز قنبری	دکترای تخصصی مامایی	استادیار		۰۹۱۴۱۰۹۶۸۰۰

۲۱- راهنمای تکمیل طرح پیشنهادی تحقیق را بدقت مطالعه، ضمن موافقت با آن، صحت مطالب مندرج در آن را تأئید می نمایم.

نام و نام خانوادگی و امضای
مجری طرح / استاد راهنما:

نام و نام خانوادگی و امضای مسئول محل یا
محلهای اجرای طرح/ پایاننامه:

عنوان تعهد	توضیحات
رضایت آگاهانه	
پرسشنامه	

Students Informed Cosent.pdf
Questionnaires.pdf
Womens Informed Cosent.pdf
