# Supplementary material for: Effect of midwifery students’ continuity of care program on women’s experiences of maternity care: A randomised controlled trial
Source: PLoS One. 2026 Jul 24;21(7):e0353118. doi: 10.1371/journal.pone.0353118 (PMC13399285; doi:10.1371/journal.pone.0353118)
Supplement: S4 Appendix — (DOCX) [file pone.0353118.s004.docx]

**Personal, social, and** **obstetrics characteristics**

| Participant code: ……………………………... |
| --- |
| Date of completion of the questionnaire: ……………………. |
| Covered by health center No: ………………………………... |
| Mobile number: .................................. |
| Mobile number of husband, mother, or sister: |
| Name and surname of the principal student: …………………. |
| Name and surname of the supporting student: ………………… |

| **Social characteristics:** | |  |  |  |
| --- | --- | --- | --- | --- |
| Year of birth:…........................... | |  |  |  |
| Marriage age: ……………….years | |  |  |  |
| Education level: | Secondary school□ | High school□ | Diploma□ | University□ |
|  |  |  |  |  |
| Job: | Housekeeper□ | Employment outside the home□ | Employment inside the home□ |  |
|  |  |  |  |  |
| Husband's education level: | Illiterate□ | Elementary school □ | Secondary school□ | High school□ |
|  | Diploma□ | University□ |  |  |
|  |  |  |  |  |
| Husband's job: | Unemployed□ | Worker□ | Self-employed□ | Governmental job□ |
|  |  |  |  |  |
| Housing status: | Private house□ | Government-sponsored-low-cost house□ | Rented house□ | Relative’s house□ |
|  | | | | |
| **Obstetrics characteristics**: | | | | |
| The date of the first ultrasound in pregnancy: ………………………………. | | | | |
| Gestational age based on the first ultrasound: ………………………………..  The first day of the last menstrual period: …………………………………… | | | | |
|  | | | | |
| Gravida: | Parity: | The number of abortions: | The number of living children: | The number of dead children: |
|  |  |  |  |  |
| Planning for pregnancy: | Planned pregnancy□ | Unplanned pregnancy□ | Unwanted pregnancy□ |  |
|  |  |  |  |  |
|  |  |  |  |  |
| History of infertility: | Yes/primary infertility□  ….years | Yes/secondary infertility□  …..years | No□ |  |
|  |  |  |  |  |
| Your main caregiver/caregivers during pregnancy: | Obstetrician□ | Midwife□ | Both of them□ |  |
|  |  |  |  |  |
| Intended Place for delivery: ………...  Weight before pregnancy….……kg | | Weight in the first trimester of pregnancy……kg | | Hight…..cm |

**Childbirth and newborn characteristics questionnaire**

| **Childbirth characteristics** | | | | |
| --- | --- | --- | --- | --- |
| Participant code: | | | | |
| Date and time of birth: ……………………….. | | | | |
| Hospital name: …………………………………….. | | | |  |
| The student with mother: | Main care provider□ | | Supporter□ | |
|  |  | |  | |
| How to inform the student about the start of labor: | Call□ | SMS□ | Contact through husband□ | Not notified□  The reason  ……………. |
|  |  |  |  |  |
| Was the student present in labor? | Yes□  From time....... date.......  By the time........date...... | | No□  The reason  ……………….. | |
|  |  | |  | |
| The main cause of the mother's hospitalization: | Labor pain□ | Bleeding□ | Rupture of membranes□ | Decreased fetal movement□ |
|  |  |  |  |  |
|  | Decreased amniotic fluid□ | Post term□ | Other reasons….. | |
|  |  |  |  |  |
| Method of termination of pregnancy: | Spontaneous initiation of labor□ | Induction with oxytocin□ | Foley catheter for induction of labor□ | Misoprostol□ |
|  | Other cases................. |  |  |  |
|  |  |  |  |  |
| Mother's condition on arrival: | Dilatation…cm | Effacement% | Station……. | Membrane…..  (Intact/Rupture) |
|  | Presentation……. |  |  |  |
|  |  |  |  |  |
| Type of birth: | Spontaneous vaginal birth□ | Elective cesarean section□ | Vacuum-assisted vaginal birth□ | Emergency cesarean section□  Reason………. |
|  |  |  |  |  |
| Birth risk factors: | Premature rupture of membranes for 18 hours or longer□ | placenta abruption□ | Meconium-stained amniotic fluid□ | Abnormal placental adhesion□ |
|  |  |  |  |  |
|  | Fetal arrhythmias□ | Other cases................ |  |  |
|  |  |  |  |  |
| Interventions during childbirth: | Episiotomy□ | Amniotomy□ | oxytocin-stimulated labor□ | Vacuum□ |
|  | Epidural anesthesia□ | Spinal anesthesia□ | Fentanyl infusion□ | Intramuscular injection of pethidine□ |
|  | Intramuscular injection of promethazine□ | Manual removal of the placenta□ | No intervention□ | Other interventions□ |
|  |  |  |  |  |
| The primary caregiver in labor | Midwife□ | obstetrician□ | Midwifery instructor□ | Obstetrician-gynecologist resident□ |
|  |  |  |  |  |
| Student participation in birth: | The main student providing continuity of care performed the birth□ | The supporter student did the birth□ | The main and supporter students did not perform the birth□  The reason:…….. | |
|  |  |  |  |  |
| Partograph: | Left side of the alert line□ | The alert line has been crossed□ | The action line has been crossed□ | |
|  |  |  |  |  |
| Complications during childbirth: | Receiving blood and blood products□ | perineal tear grade 1□ | perineal tear grade 2□ | perineal tears grades 3-4□ |
|  |  |  |  |  |
| Use of drugs to control post-partum bleeding: | Oxytocin as per routine□ | High dose oxytocin□  Dosage and method of use……. | Methylergonovine□ | Misoprostol□  Dosage and method of use…………….. |
|  |  |  |  |  |
| Mother's condition up to 2 hours after delivery: | Transfer to postpartum ward□ | Transfer to the operating room□  The reason…… | Transfer to the operating room□  The reason…… | Transfer to the intensive care unit□  The reason…… |
|  | death of mother□  The reason…… |  |  |  |
|  |  |  |  |  |
| Did the woman have another companion besides the student? | Yes□ | No□ |  |  |
|  |  |  |  |  |
| Hematocrit: | Hematocrit at the time of admission to the hospital:………….. | | Hematocrit 24 hours after delivery:……………… | |
|  |  |  |  |  |
| **Newborn characteristics** | | | | |
| Sex of the baby: | Female□ | Male□ | Ambiguous genitalia□ | |
|  |  |  |  |  |
| Weight:…….g | Hight………cm | Head circumference………..cm | Apgar score at 1 minute….. | Apgar scored at 5 minutes……. |
|  |  |  |  |  |
| Need for resuscitation: | No□ | Yes, initial resuscitation steps □ | Yes, advanced resuscitation steps□ |  |
|  |  |  |  |  |
| Breastfeeding in the first hour after birth: | Yes □ | No□ The reason:…… | |  |
|  |  |  |  |  |
| Newborn's condition: | Rooming-in with mother in postpartum ward□ | Admission to the neonatal ward□ | Admission to the neonatal intensive care unit□ | Intrauterine death□ |
|  | Neonatal death□ | The reason:………….. | |  |
|  |  |  |  |  |
| The condition of the newborn after the mother's discharge: | He/she was discharged with his mother□ | Hospitalization in the intensive care unit□ | Hospitalization in the neonatal ward□ | |
