# Supplementary material for: Effect of midwifery students’ continuity of care program on women’s experiences of maternity care: A randomised controlled trial
Source: PLoS One. 2026 Jul 24;21(7):e0353118. doi: 10.1371/journal.pone.0353118 (PMC13399285; doi:10.1371/journal.pone.0353118)
Supplement: Attachment — Submitted filename: Response to Reviewers.docx [file pone.0353118.s007.docx]

**Dear Scientific Editor, PLOS One**

We greatly appreciate your and the reviewers’ insightful critiques of our manuscript (Manuscript PONE-D-25-51531) titled "**Effect of midwifery students’ continuity of care program on women’s experiences of maternity care: a randomised controlled trial**" and your constructive comments and suggestions. We have diligently addressed all of your suggestions and believe that the manuscript has significantly improved as a result. We hope that you will now consider it suitable for publication in the Journal. Below, you will find our detailed point-by-point responses to the comments. We also made all necessary changes in the manuscript as you suggested.

Yours sincerely,

Sakineh Mohammad-Alizadeh-Charandabi

***Response to the Academic Editor***

**Comment 1: A rebuttal letter that responds to each point raised by the academic editor and reviewer(s). You should upload this letter as a separate file labeled 'Response to Reviewers'.**

**Response:** We thank the academic editor and reviewers for their time and valuable feedback on our manuscript. A detailed rebuttal letter titled “Response to Reviewers” has been prepared and uploaded as a separate file.

**Comment 2: A marked-up copy of your manuscript that highlights changes made to the original version. You should upload this as a separate file labeled 'Revised Manuscript with Track Changes'.**

**Response:** We have uploaded a marked-up copy of the manuscript with all changes highlighted. The file is labeled “Revised Manuscript with Track Changes” and clearly indicates all modifications made in response to the comments.

**Comment 3: An unmarked version of your revised paper without tracked changes. You should upload this as a separate file labeled 'Manuscript'.**

**Response:** An unmarked version of the revised manuscript, without tracked changes, has been uploaded as a separate file labeled “Manuscript”.

**Comment 4: If you would like to make changes to your financial disclosure, please include your updated statement in your cover letter.**

**Response:** We included our updated financial disclosure statement in the cover letter.

**Comment 5: Guidelines for resubmitting your figure files are available below the reviewer comments at the end of this letter.**

**Response:** We used the guidelines for resubmitting our figure file.

**Comment 6: If applicable, we recommend that you deposit your laboratory protocols in protocols.io to enhance the reproducibility of your results.**

**Response:** This manuscript has been extracted from an extensive research project entitled “Implementation and evaluation of continuous care model by midwifery students during pregnancy, childbirth and postpartum: a mixed-methods design with Embedded Experimental Model”. The Persian version of the study protocol (including the English abstract) was registered on the University website before acceptance and receipt of an ethical code for the research conduct. The Persian and English versions of the protocol have been provided in the supplementary materials. (Page 29, line 8)

***Response to Journal Requirements:***

**Comment 1: Please ensure that your manuscript meets PLOS ONE's style requirements, including those for file naming.**

**Response:** We have reviewed the PLOS ONE style requirements and ensured that the manuscript and accompanying files adhere to the specified guidelines, including proper file-naming conventions. All files have been renamed accordingly to the journal's requirements.

**Comment 2: We note that the original protocol that you have uploaded as a Supporting Information file contains an institutional logo. As this logo is likely copyrighted, we ask that you please remove it from this file and upload an updated version upon resubmission.**

**Response:** We removed the institutional logo from the original protocol in the Supporting Information file, as requested. An updated version of the protocol without the logo has been uploaded upon resubmission. (Page 29, line 8)

**Comment 3: Thank you for stating the following in the Acknowledgments Section of your manuscript: “The authors express their gratitude to the Vice-Chancellor for Research of Tabriz University of Medical Sciences for financial support. They would also like to sincerely thank the midwifery students who participated in this trial as care providers and the women who took part in the study. Furthermore, they acknowledge the valuable assistance offered by the Clinical Research Development Unit of Taleghani Hospital, Tabriz University of Medical Sciences, Tabriz, Iran.”**

**We note that you have provided funding information that is not currently declared in your Funding Statement. However, funding information should not appear in the Acknowledgments section or other areas of your manuscript. We will only publish funding information present in the Funding Statement section of the online submission form. Please remove any funding-related text from the manuscript and let us know how you would like to update your Funding Statement. Currently, your Funding Statement reads as follows: “This research was funded by the Vice-Chancellor for Research at Tabriz University of Medical Sciences in Tabriz, Iran (Scientific Grant No. 69665, E.J., M.M., S.A., L.D., S.M.A.C.). The funders were not involved in the study design, participant recruitment, data collection and analysis, data interpretation, manuscript preparation, or the decision to submit the article for publication.” Please include your amended statements within your cover letter; we will change the online submission form on your behalf.**

**Response:** In compliance with your suggestion, we have removed the funding-related text from the Acknowledgments section of the manuscript. The revised Acknowledgments now read: “The authors sincerely thank the midwifery students who served as care providers in this trial, as well as the women who participated in the study. Furthermore, they acknowledge the valuable assistance offered by the Clinical Research Development Unit of Taleghani Hospital, Tabriz University of Medical Sciences, Tabriz, Iran.” (page 29, line 19)

Regarding the Funding Statement, we would like to update it as follows: “This research was funded by the Vice-Chancellor for Research at Tabriz University of Medical Sciences in Tabriz, Iran (Scientific Grant No. 69665, S.M.A.C.). The funder has no role in the study design, participant recruitment, data collection and analysis, data interpretation, manuscript preparation, or the decision to submit the paper for publication.” We kindly ask that you update the online submission form with this amended statement.

**Comment 4: In the online submission form, you indicated that “The corresponding author will provide de-identified participant datasets for research purposes to qualified researchers affiliated with academic institutions and other entities upon reasonable request, immediately after the publication of the findings.”**

**All PLOS journals now require all data underlying the findings described in their manuscript to be freely available to other researchers, either 1. In a public repository, 2. Within the manuscript itself, or 3. Uploaded as supplementary information. This policy applies to all data except where public deposition would breach compliance with the protocol approved by your research ethics board. If your data cannot be made publicly available for ethical or legal reasons (e.g., public availability would compromise patient privacy), please explain your reasons on resubmission and your exemption request will be escalated for approval.**

**Response:** We prepared all data underlying the findings described in this manuscript (including variable and value definitions) as an Excel file and uploaded it as an S1 Appendix. (page 29, line 9)

**Comment 5: Your ethics statement should only appear in the Methods section of your manuscript. If your ethics statement is written in any section besides the Methods, please move it to the Methods section and delete it from any other section. Please ensure that your ethics statement is included in your manuscript, as the ethics statement entered into the online submission form will not be published alongside your manuscript.**

**Response:** We moved the ethics statement to the Methods section (page 7, lines 5-6; page 8, lines 10-11) and removed it from other sections as requested.

**Comment 6: Please include a caption for figure.**

**Response:** We have added the following caption to the Figure:

"Figure: Flow diagram of the trial

MSCOC: Midwifery Student Continuity of Care

* Individuals who experienced labour pain.

^**^Individuals who had a normal birth." (page 15, lines 18-21)

**Comment 7: Please ensure that you refer to Figure 1 in your text as, if accepted, production will need this reference to link the reader to the figure.**

**Response:** We have referred to Figure in the text on page 15, line 17. This ensures proper linking of the figure within the manuscript.

**Comment 8: Please include captions for your Supporting Information files at the end of your manuscript, and update any in-text citations to match accordingly. Please see our Supporting Information guidelines for more information:** [**http://journals.plos.org/plosone/s/supporting-information**](http://journals.plos.org/plosone/s/supporting-information)**.**

**Response:** We added captions for all Supporting Information files at the end of the manuscript (page 29, line 8).

**Comment 9: If the reviewer comments include a recommendation to cite specific previously published works, please review and evaluate these publications to determine whether they are relevant and should be cited. There is no requirement to cite these works unless the editor has indicated otherwise.**

**Response:** The reviewers have not recommended citing any specific previously published works.

**Comment 10: Please review your reference list to ensure that it is complete and correct. If you have cited papers that have been retracted, please include the rationale for doing so in the manuscript text, or remove these references and replace them with relevant current references. Any changes to the reference list should be mentioned in the rebuttal letter that accompanies your revised manuscript. If you need to cite a retracted article, indicate the article’s retracted status in the References list and also include a citation and full reference for the retraction notice.**

**Response:** We have carefully rechecked the reference list, and none of the cited papers have been retracted. In response to the reviewers’ comments, we have added several new references, all of which are marked with track changes and highlighted in the manuscript.

***Response to Additional Editor Comments:***

**Comment 1: Information about the care received by the control group is essential.**

**Response:** Both groups received routine care throughout pregnancy, labor, and the postpartum period. We have added details about this care and the childbirth preparation classes at the end of the Intervention section (pages 11, lines 9-22; page 12, lines 1-10).

**Comment 2: Were mothers in the control group free to attend childbirth preparation classes?**

**Response:** Yes, both groups were free to participate in childbirth preparation classes. We have added this information to the manuscript (page 11, lines 9-10). However, most pregnant women in Iran do not attend the classes.

Among the women who experienced labor pain, 2 women (5.4%) in the MSCOC group attended the classes, compared to 5 women (14.3%) in the control group. The frequency of participation was included in Table 1.

**Comment 3: Did mothers in the intervention group have the option of being accompanied during labor by a midwife or the delivering physician?**

**Response:** In Iran, physicians typically do not accompany women during labor. The accompanying midwife, usually unfamiliar with the woman beforehand, attends the labor at the mother’s request as a private midwife. She provides ongoing emotional and psychological support but does not participate in clinical decision-making or medical care.

Both groups had the option to be accompanied during labor by a private midwife or a female family member, depending on hospital policies. This information has been added to the manuscript (page 11, lines 10-12).

Among women who experienced labor pain, 2 (5.4%) in the MSCOC group were accompanied by a midwife, and 9 (24.3%) by a family member. In the control group, 4 (11.4%) were accompanied by a midwife, and 8 (23%) by a family member. The frequencies were also included in Table 1.

**Comment 4: The reasons for elective cesarean section should be compared between the two groups.**

**Response:** Among women who chose elective cesarean sections, two in the MSCOC group did so with their obstetrician's encouragement, while one chose it at her own request. In the control group, four women underwent the procedure with the obstetrician's encouragement, one was motivated by her mother-in-law, and six at their own request. Also, one woman in the intervention group and two women in the control group had cesarean sections due to breech presentation. This information has been added to the manuscript. (page 22, lines 14-17).

**Comment 5: Why weren’t women who requested an elective cesarean section excluded from the study during recruitment?**

**Response:** As stated in the Participants section of the manuscript, one of the eligibility criteria was "willingness to have a vaginal birth at one of nine city-level main hospitals" (page 7, line 15). Therefore, women who requested an elective cesarean section were excluded during recruitment. However, since our study followed an intention-to-treat design, women who later changed their preference and requested an elective cesarean section during the study were not excluded and followed-up until 40-50 days after delivery.

***Response to Reviewer #1:***

**Comment 1: Given that assessing outcomes 40-50 days after delivery can affect the accuracy of individuals' responses and the effectiveness of interventions, the reason for choosing this time and its justification are important.**

**Response:** Midwife-led continuity of care begins during pregnancy and extends through labor, often into the early postpartum period [1, 2]. Accordingly, our intervention started at 26–29 weeks of gestation and continued until 20–30 days postpartum. Therefore, outcomes which may also be influenced by postnatal care, such as the experience of maternity care [3] and breastfeeding self-efficacy [4], were assessed 20 to 30 days after the intervention ended.

Childbirth experience was our primary outcome. The Childbirth Experience Questionnaire (CEQ-2) is typically administered between 4 and 8 weeks postpartum [5-7]. Research suggests that measuring the childbirth experience too soon may yield overly positive responses, influenced by the immediate happiness of having a healthy child. Allowing a period of reflection leads to a more stable and accurate representation of a woman's birth experience [7, 8].

An explanatory note regarding this timing has been added to the revised manuscript. (page 12, lines 13-14 and 18-20)

**Comment 2: Didn't the difference in the students who served as instructors and interventionists affect the results?**

**Response:** We have described the continuity of care during labor and delivery in the intervention section of the manuscript (page 10, lines 18-22). Except for one case, the same student midwife who conducted the educational sessions during pregnancy and the postpartum period also attended the woman during labor and childbirth as a supporter. In none of the cases, the student had no role in decision-making for medical interventions or in childbirth (page 17, lines 7-9)

**Comment 3: In cases where the trainer was not present at the time of delivery or was notified later, the sample had to be excluded from the study.**

**Response:** We followed a pre-planned modified intention-to-treat approach, following all recruited participants until the end of the trial and assessing all outcomes that could be assessed, without considering their protocol adherence. All participants were included in the analysis, in the groups to which they were originally assigned, excluding only those whose outcomes could not be assessed (page 15, lines 3-4). However, detail of protocol adherence was described in the Results section of the manuscript (page 17, lines 4-9).

***Response to Reviewer #2***

**Comment 1: Clarify the sample size was determined based on a t-test.**

**Response:** We clarified that “The sample size was calculated using t-test for difference between two independent means in G*Power software.” (page 8, lines 13-14)

**Comment 2: Table 1: Add parity. Present Table 1 for the randomized set (Intervention n=45; Control n=48), as comparisons of maternity-care experience scores were conducted in the randomized set.**

**Response:** The comparison of parity between the two groups was presented in Table 1 (row 10, Primiparous).

Our primary outcome reported in this paper (childbirth experience (CEQ-2)), as well as some secondary outcomes, including the experience of maternity care during labor and birth (EMC-LB) and support and control in birth (SCIB), were assessed exclusively in women who experienced labor pain. As stated in the Statistical Analysis section (page 15, lines 3-4), "Our analyses followed a modified intention-to-treat approach, excluding participants who did not experience labor pain from CEQ-2, SCIB, and EMC-LB."

The other secondary outcomes were assessed and analyzed in all randomized participants. The baseline characteristics of all randomized participants have been reported in the other paper currently under review in another journal, as pointed out in the cover letter and the method section of the current manuscript (page 7, lines 2-4). Furthermore, in the Results section, we noted that "when considering all women, the two groups exhibited similar baseline characteristics as detailed in the paper under review." (page 16, lines 7-9). We also provided the raw data (as an Excel file) for all randomized participants and uploaded it as an S1 Appendix (page 29, line 9).

**Comment 3: Given so many outcomes, adjust p-values for multiplicity.**

**Response:** As we reported on page 12, lines 12-13, only the childbirth experience (total CEQ-2 score) was the primary outcome, and the others were secondary outcomes. According to the CONSORT (2025, page 17), “Having several **primary** outcomes can incur potential problems of interpretation associated with multiplicity of analyses.” [9]

**Comment 4: Clearly justify use of last observation carried forward, stating its assumption of no change after the last measurement.**

**Response:** We actually imputed only four missed value, just for one secondary outcome and justification was clarified both in the analysis sub-section as “For the outcome related to EMC-PR, as there was no significant interaction effect of post-intervention time and group on this outcome, we used data collected 40–50 days postpartum to impute values for four individuals (two in each group) who were lost to follow-up at 35–36 weeks of pregnancy.” (page 14, lines 20-21; page 15, lines 1-2), as well as in the footnote of the related table (Table 3). (page 19, lines 7-8)

***Response to Reviewer #3***

**Comment 1: p.14, Lines 13–16; Breech presentation is typically considered an indication for elective cesarean section. Shouldn’t cases involving breech presentation be categorized accordingly under elective cesarean sections?**

**Response:** In our context, breech presentation is regarded as a medical indication for cesarean section due to the overmedicalization of childbirth. A population-based study in Iran found that more than 95% of primiparous women and over 88% of multiparous women with breech presentation underwent cesarean sections [10]. However, as breech presentation is typically considered an indication for elective cesarean section, we corrected the sentence as “The reasons for no experience of labour pain were elective caesarean sections (4 vs. 10, including breech presentations (1 vs. 1)), and emergency caesarean sections performed before the active phase of labour (4 vs 3) (page 16, lines 1-2).

Accordingly, we re-ran the analysis of the mode of delivery, including breech cases under elective caesarean sections, and revised the text as follows: “In the intervention group, 33 women (73.3%) had a normal vaginal birth, 8 (17.8%) underwent emergency caesarean section, and 4 (8.9%) had elective caesarean section, one due to breech presentation. In the control group, the corresponding numbers were 30 (62.5%), 5 (10.4%), and 13 (27.1%), with two elective cases due to breech presentation. Overall, there was no statistically significant difference between the two groups in the mode of delivery (P = 0.06).” (page 22, lines 9-13)

**Comment 2: p.14, Lines 20–21; The sentence states: “Between-group comparisons of women who experienced labor pain showed that the intervention and control groups were similar in the baseline characteristics (Table 1).” However, on p.14, line 13, it is noted that 8 women in the intervention group and 13 women in the control group did not experience labor pain and underwent cesarean sections. This raises the question: does Table 1 include women who did not experience labor pain?**

**Response:** In Table 1, the comparison was made between 37 women (45 randomized women -8 women with no experience of labour pain) in the intervention group and 35 women (48-13) in the control group who experienced labor pain (page 16, Table 1), because primary and some secondary outcomes can be assessed among those participants who had experienced labor pain. We also clarify in the Results section (page 15, lines 14-16) that "93 eligible women were randomly assigned to the MSCOC group (n = 45) or the control group (n = 48).”

**Comment 3: Is it correct to interpret that ultimately, 8 out of 37 women in the intervention group and 13 out of 35 women in the control group underwent cesarean sections?**

**Response:** Actually, 12 out of 45 women in the intervention group and 18 out of 48 women in the control group underwent cesarean sections. We clearly reported this as “In the intervention group, 33 women (73.3%) had a normal vaginal birth, 8 (17.8%) underwent emergency caesarean section, and 4 (8.9%) had elective caesarean section, one due to breech presentation. In the control group, the corresponding numbers were 30 (62.5%), 5 (10.4%), and 13 (27.1%), with two elective cases due to breech presentation. Overall, there was no statistically significant difference between the two groups in the mode of delivery (P = 0.06).” (page 22, line 9-13) However, 9 out of the 30 women who underwent caesarean sections had experienced labour pain.

**Comment 4: p.15, Line 13; The manuscript states: “Midwifery students in Iran have limited opportunities to gain practical experience in midwifery centered care during their four-year bachelor course.” Is it common in Iran for individuals to enter a four-year midwifery program directly after graduating from high school? In many countries, midwifery education requires prior clinical experience as a registered nurse. Were the midwifery students in this study individuals with no prior hospital experience? It would be helpful to include a brief description of the students’ educational and clinical backgrounds.**

**Response:** We have added the following information: "In Iran, midwifery bachelor's students are admitted through a national exam after high school and complete 130 theoretical and clinical courses over four years. Advanced studies are available at the master’s and doctoral levels." (page 5, lines 13-15).

**Comment 5: p.20, Line 13; In addition to reporting the average time from admission to delivery for both groups, including the shortest and longest durations would enrich the analysis and provide a more comprehensive understanding of the variability in labor experiences.**

**Response:** We added the following information: “The shortest and longest durations in the MCOC group were 30 minutes and 15 hours, respectively, whereas in the control group they ranged from 1 to 23 hours” (page 22, lines 20-22).

**Comment 6: p.23, Line 10; The font size of the word “essential” appears smaller than the surrounding text. Please check for formatting inconsistencies.**

**Response:** We corrected the formatting issue, and the font size of the word "essential" now matches the surrounding text. (page 25, line 7)

**Comment 7: p.25, Line 2; Iran’s cesarean section rate of 52% is notably high. It seems likely that factors beyond medical necessity—such as maternal preference—may contribute to this rate. Would the authors consider discussing potential sociocultural or systemic influences on cesarean section rates in Iran?**

**Response:** We appreciate the reviewer’s suggestion to discuss sociocultural and systemic influences on the high cesarean section rate in Iran. In response, we have expanded our discussion to address the factors contributing to the high cesarean rate beyond medical necessity. Specifically, we have included the influence of socioeconomic factors, cultural norms, and systemic issues. We also highlight the role of the private healthcare sector, financial incentives, and maternal preferences as key drivers. Additionally, we discuss potential interventions such as improving childbirth preparation, enhancing midwifery autonomy, and integrating care to promote vaginal delivery and reduce cesarean rates (page 26, lines 22-23; page 27, lines 1-11).

References

1.	Bradford BF, Wilson AN, Portela A, McConville F, Fernandez Turienzo C, Homer CSE: **Midwifery continuity of care: A scoping review of where, how, by whom and for whom?** *PLOS global public health* 2022, **2**(10):e0000935.

2.	Sandall J, Fernandez Turienzo C, Devane D, Soltani H, Gillespie P, Gates S, Jones LV, Shennan AH, Rayment-Jones H: **Midwife continuity of care models versus other models of care for childbearing women**. *The Cochrane database of systematic reviews* 2024, **4**(4):Cd004667.

3.	Redshaw M, Martin CR, Savage-McGlynn E, Harrison S: **Women's experiences of maternity care in England: preliminary development of a standard measure**. *BMC pregnancy and childbirth* 2019, **19**(1):167.

4.	Dennis CL, Faux S: **Development and psychometric testing of the Breastfeeding Self-Efficacy Scale**. *Research in nursing & health* 1999, **22**(5):399-409.

5.	Walker KF, Dencker A, Thornton JG: **Childbirth experience questionnaire 2: Validating its use in the United Kingdom**. *European journal of obstetrics & gynecology and reproductive biology: X* 2020, **5**:100097.

6.	Boie S, Lauridsen HH, Glavind J, Smed MK, Uldbjerg N, Bor P: **The Childbirth Experience Questionnaire (CEQ)-Validation of its use in a Danish-speaking population of new mothers stimulated with oxytocin during labour**. *PloS one* 2020, **15**(5):e0233122.

7.	Lyngbye K, Melgaard D, Lindblad V, Kragholm KH, Eidhammer A, Westmark S, Maimburg RD: **Do women's perceptions of their childbirth experiences change over time? A six-week follow-up study in a Danish population**. *Midwifery* 2022, **113**:103429.

8.	Maimburg RD, Væth M, Dahlen H: **Women's experience of childbirth - A five year follow-up of the randomised controlled trial "Ready for Child Trial"**. *Women and birth : journal of the Australian College of Midwives* 2016, **29**(5):450-454.

9.	Hopewell S, Chan A-W, Collins GS, Hróbjartsson A, Moher D, Schulz KF, Tunn R, Aggarwal R, Berkwits M, Berlin JA *et al*: **CONSORT 2025 explanation and elaboration: updated guideline for reporting randomised trials**. *BMJ* 2025, **389**:e081124.

10.	Pourshirazi M, Heidarzadeh M, Taheri M, Esmaily H, Babaey F, Talkhi N, Gholizadeh L: **Cesarean delivery in Iran: a population-based analysis using the Robson classification system**. *BMC pregnancy and childbirth* 2022, **22**(1):185.
