# Supplementary material for: Effect of midwifery students’ continuity of care program on women’s experiences of maternity care: A randomised controlled trial
Source: PLoS One. 2026 Jul 24;21(7):e0353118. doi: 10.1371/journal.pone.0353118 (PMC13399285; doi:10.1371/journal.pone.0353118)
Supplement: Attachment — Submitted filename: Response to the queries.docx [file pone.0353118.s006.docx]

**Subject:** Response to Editorial Queries

**Manuscript ID:** PONE-D-25-51531

**Manuscript Title:** Effect of midwifery students’ continuity of care program on women’s experiences of maternity care: a randomised controlled trial

**Dear Dr Reynante Peña Balsamo,**

We sincerely appreciate your careful evaluation of our submission and the opportunity to revise and resubmit our manuscript. In line with the journal’s requirements, we have addressed all editorial queries in detail. Our point-by-point responses are provided below. We also edited the manuscript based on the comments.

Yours sincerely

Sakineh Mohammad-Alizadeh-Charandabi

Department of Midwifery, Faculty of Nursing and Midwifery, Tabriz University of Medical Sciences

1. *Please complete all items on the Human Participants Research Checklist that are relevant for your submission, by following this link: http://journals.plos.org/plosone/s/file?id=dc11/PLOSOne_Human_Subjects_Research_Checklist.docx (Contact us at plosone@plos.org if you cannot access the document.) There may be overlap between the checklist items and other queries listed below; please address any duplicated queries both in your response email and on the checklist itself. Upload the completed Human Participants Research Checklist as file type “Other” when you re-submit your manuscript. This document is for internal journal use only and will not be published if your article is accepted. The requested information will help us to assess whether your submission complies with PLOS ONE’s policies and adheres to applicable reporting standards. Note that your manuscript may be rejected if you provide incomplete or inadequate responses to the checklist questions and that changing the ‘Section/Category’ of your article does not affect this requirement.*

***Response:*** We have completed the *PLOS Human Participants Research Checklist* and uploaded it as file of type “Other.” In accordance with the journal’s instructions, any overlapping items have been addressed both in the checklist and in this response letter.

2. *Please provide additional details regarding participant consent. In the ethics statement in the Methods and online submission information, please ensure that you have specified what type you obtained (for instance, written or verbal, and if verbal, how it was documented and witnessed). If your study included minors, state whether you obtained consent from parents or guardians. If the need for consent was waived by the ethics committee, please include this information.*

*Once you have amended this/these statement(s) in the Methods section of the manuscript, please add the same text to the “Ethics Statement” field of the submission form (via “Edit Submission”).*

*For additional information about PLOS ONE ethical requirements for human subjects research, please refer to* [*http://journals.plos.org/plosone/s/submission-guidelines#loc-human-subjects-research*](http://journals.plos.org/plosone/s/submission-guidelines#loc-human-subjects-research)*.*

***Response****:* Written informed consent was obtained from all participants, as indicated in the submission system (Information section) and reported in the manuscript (Methods, p. 8, lines 8–9; Ethics Declaration, p. 28, lines 2–3). No minors participated; thus, parental or guardian consent was not required.

3. *We note that you have selected “Clinical Trial” as your article type. PLOS ONE requires that all clinical trials are registered in an appropriate registry (the WHO list of approved registries is at* [*https://www.who.int/clinical-trials-registry-platform/network/primary-registries*](https://www.who.int/clinical-trials-registry-platform/network/primary-registries)*""* [*https://www.who.int/clinical-trials-registry-platform/network/primary-registries*](https://www.who.int/clinical-trials-registry-platform/network/primary-registries) *and more information on trial registration is at* [*http://www.icmje.org/about-icmje/faqs/clinical-trials-registration/*](http://www.icmje.org/about-icmje/faqs/clinical-trials-registration/)*).

   Please state the name of the registry and the registration number (e.g. ISRCTN or ClinicalTrials.gov) in the submission data and on the title page of your manuscript.
   a) Please provide the complete date range for participant recruitment and follow-up in the methods section of your manuscript.
   b) If you have not yet registered your trial in an appropriate registry, we now require you to do so and will need confirmation of the trial registry number before we can pass your paper to the next stage of review. Please include in the Methods section of your paper your reasons for not registering this study before enrolment of participants started. Please confirm that all related trials are registered by stating: “The authors confirm that all ongoing and related trials for this drug/intervention are registered”.

   Please see* [*http://journals.plos.org/plosone/s/submission-guidelines#loc-clinical-trials*](http://journals.plos.org/plosone/s/submission-guidelines#loc-clinical-trials) *for our policies on clinical trials.*

***Response:*** The trial was prospectively registered in the Iranian Registry of Clinical Trials (IRCT20100414003706N41) on 30 April 2022 (<https://trialsearch.who.int/Trial2.aspx?TrialID=IRCT20100414003706N41>;

<https://irct.behdasht.gov.ir/trial/62761>)

a) This information is reported on page 3 (lines 6-9) and included on the title page.
b) Participant recruitment and follow-up dates are fully specified in the manuscript. “Women were recruited from November 2022 to February 2023, with follow-up completed in July 2023” (Results, p. 14, lines 7–8).
4. *Please note that you must upload a completed CONSORT flowchart as figure 1 of your manuscript. Blank copies of this document and information regarding CONSORT can be found via the following link: (*[*https://www.equator-network.org/reporting-guidelines/consort/*](https://www.equator-network.org/reporting-guidelines/consort/)*).*

***Response:*** The CONSORT flowchart has now been uploaded as a figure for review.

5. *Please upload a copy of your trial study protocol as a supporting information file. By the study protocol, we mean the complete and detailed plan for the conduct and analysis of the trial that the ethics committee approved before the trial began. Please send this in the original language. If this is in a language other than English, please also provide a translation. Please detail any deviations from this study protocol in the Methods section of your manuscript. Your study protocol will be made available to the editors and reviewers, and will be published as supporting information with your manuscript if accepted for publication. (If you do not agree to this, we will not be able to publish your manuscript). If you have formally published a study protocol for your trial in a journal then you should cite this in your manuscript, but you still need to send us the original document.*

***Response:*** In accordance with the journal’s requirements, the complete study protocol, approved by the ethics committee before trial initiation, has been uploaded as a Supporting Information file. The original protocol is provided in Persian with an English abstract accessible through https://pazhoohan.tbzmed.ac.ir/webdocument/load.action?webdocument_code=10&masterCode=91069665, accompanied by an English translation.

As noted in our cover letter, a related companion manuscript with no overlapping results—submitted as an “Other” file and currently under review—was submitted simultaneously and disclosed to the journal. The protocol includes information about both manuscripts.

Any deviations from the approved protocol are clearly described in the Methods section (p. 7, lines 13–18). Specifically, approximately two months after recruitment began, slower-than-anticipated enrollment necessitated expanding the study setting from two teaching hospitals to all city-level hospitals with high delivery rates, following research council approval. This expansion included one social security hospital and six private or semi-private hospitals, while one hospital was excluded due to a lack of a permit. To account for potential differences by hospital type, participant allocation was stratified accordingly (teaching, social security, private, and semi-private).
