# Supplementary material for: Effect of midwifery students’ continuity of care program on women’s experiences of maternity care: A randomised controlled trial
Source: PLoS One. 2026 Jul 24;21(7):e0353118. doi: 10.1371/journal.pone.0353118 (PMC13399285; doi:10.1371/journal.pone.0353118)
Supplement: Attachment — Submitted filename: Response to Reviewers (2).docx [file pone.0353118.s008.docx]

**Dear Dr Reem Saeed Alghamdi,**

**Guest Editor, PLOS One**

We sincerely thank you and the reviewers for the careful evaluation of our revised manuscript (Manuscript ID: PONE-D-25-51531) entitled *“Effect of midwifery students’ continuity of care program on women’s experiences of maternity care: a randomised controlled trial.”*

We appreciate the opportunity to further improve the manuscript. We have addressed all comments and revised the manuscript accordingly. All changes are clearly marked in the tracked version of the manuscript, and the corresponding page and line numbers are provided for each modification.

We hope that the revised manuscript now meets the publication standards of *PLOS ONE*.

Yours sincerely,

Sakineh Mohammad-Alizadeh-Charandabi

***Response to the Academic Editor***

**Comment 1: If applicable, we recommend that you deposit your laboratory protocols in protocols.io to enhance the reproducibility of your results. Protocols.io assigns your protocol its own identifier (DOI) so that it can be cited independently in the future.**

**Response:** Thank you for this valuable suggestion. Although the study protocol was not registered in protocols.io, this manuscript was extracted from an extensive research project entitled “Implementation and evaluation of continuous care model by midwifery students during pregnancy, childbirth and postpartum: a mixed-methods design with Embedded Experimental Model”. The Persian version of the study protocol (including the English abstract) had been registered on the University website before obtaining ethical approval for conducting the study. To enhance transparency and reproducibility, both the Persian and English versions of the study protocol have been provided as supplementary materials. (Page 31, lines 1-6)

**Comment 2: As the corresponding author, your ORCID iD is verified in the submission system and will appear in the published article. PLOS supports the use of ORCID, and we encourage all coauthors to register for an ORCID iD and use it as well. Please encourage your coauthors to verify their ORCID iD within the submission system before final acceptance, as unverified ORCID iDs will not appear in the published article. Only the individual author can complete the verification step; PLOS staff cannot verify ORCID iDs on behalf of authors*.***

**Response:** Thank you for this suggestion. We have added ORCID IDs of all coauthors to the revised manuscript and the submission system. We all encouraged all coauthors to verify their ORCID IDs within the submission system before final acceptance.

***Response to Journal Requirements:***

**Comment 1: If the reviewer comments include a recommendation to cite specific previously published works, please review and evaluate these publications to determine whether they are relevant and should be cited. There is no requirement to cite these works unless the editor has indicated otherwise.**

**Response:** The reviewers have not recommended citing any specific previously published works.

**Comment 2: Please review your reference list to ensure that it is complete and correct. If you have cited papers that have been retracted, please include the rationale for doing so in the manuscript text, or remove these references and replace them with relevant current references. Any changes to the reference list should be mentioned in the rebuttal letter that accompanies your revised manuscript. If you need to cite a retracted article, indicate the article’s retracted status in the References list and also include a citation and full reference for the retraction notice.**

**Response:** No retracted publications were included in the reference list. All references were verified for validity and currency before resubmission.

***Response to Reviewer #3***

**Comment 1: In line with CONSORT statement, baseline characteristics should be presented descriptively without statistical testing. Therefore, I suggest removing the P value for baseline variables from Table1 to avoid potential misinterpretation.**

**Response:** We thank the reviewer for this methodological suggestion. In accordance with CONSORT guidelines, p-values for baseline characteristics have been removed. Table 1 now presents baseline variables descriptively only to avoid misinterpretation. (Table 1, page 17)

***Response to Reviewer #5***

***Abstract***

**Comment 1: Why authors started recruitment of women at 26–29 weeks?**

**Response:** Continuity-of-care models typically span pregnancy, labour, and the postpartum period; however, there is no consensus on the optimal gestational age for initiating such models [1, 2]. In this study, participants were recruited at 26–29 weeks’ gestation. The intervention was started immediately after the recruitment for the intervention group to ensure adequate time to establish continuity relationships and deliver the intervention prior to labour, while maintaining follow-up through childbirth and the postpartum period. This clarification has been added to the Methods section (page 10, lines 7-9). We have also acknowledged in the Limitations section that earlier initiation of the intervention may have increased exposure to continuity of care and potentially enhanced intervention effects (page 29, lines 10-12).

1. Sandall J, Fernandez Turienzo C, Devane D, Soltani H, Gillespie P, Gates S, Jones LV, Shennan AH, Rayment-Jones H: **Midwife continuity of care models versus other models of care for childbearing women**. *The Cochrane database of systematic reviews* 2024, **4**(4):Cd004667.

2. Bradford BF, Wilson AN, Portela A, McConville F, Fernandez Turienzo C, Homer CSE: **Midwifery continuity of care: A scoping review of where, how, by whom and for whom?** *PLOS global public health* 2022, **2**(10):e0000935

**Comment 2: Why women in the intervention group also received routine care?**

**Response:** We thank the reviewer for this important comment. The Midwifery Student Continuity of Care (MSCOC) intervention was implemented as a supplementary model integrated into the existing maternity care system, rather than as a replacement for routine services. Accordingly, participants in the intervention group received routine antenatal, intrapartum, and postpartum care in line with national guidelines, alongside continuity-of-care support delivered by trained midwifery students. This design ensured ethical provision of routine maternity care to all participants while enabling evaluation of the added effect of the MSCOC intervention within the current healthcare context. We have clarified this in the Methods section (page 12, lines 11-21; page 13, lines 1-16).

**Comment 3: How many prenatal care women received by students?**

**Response:** According to the protocol, the continuity-of-care intervention delivered by midwifery students comprised approximately nine antenatal contacts, including two in-person sessions and multiple scheduled telephone/video consultations during pregnancy. Continuity was maintained during labour, followed by four structured postpartum follow-up sessions up to six weeks after birth. This description has been clarified in the Methods section (page 10, lines 10–20).

We also reported adherence to the intervention in the Results section, showing that 34 women (76%) in the intervention group attended at least six antenatal sessions (page 18, lines 4-5).

**Comment 4: Did students implement prenatal care under the supervision?**

**Response:** Midwifery students did not provide routine antenatal care independently and did not replace licensed healthcare providers. All routine antenatal care was delivered by licensed midwives or obstetricians according to national guidelines. The role of the midwifery students was limited to providing supplementary continuity-of-care support. The students were supervised by the principal investigator, who monitored students’ performance and provided ongoing guidance and feedback throughout the study. This clarification has been added to the Methods section (page 12, lines 11-14).

***Methods***

**Comment 1: Please write the title of the large mixed method study.**

**Response:**

We thank the reviewer for this helpful suggestion. We have added the title of the overarching mixed-methods project: “Implementation and evaluation of a continuous care model by midwifery students during pregnancy, childbirth, and postpartum: a mixed-methods study with an embedded experimental design.” We have also clarified that the present manuscript reports the quantitative experimental component of this larger study. This information has been included in the Methods section (page 7, lines 2–9).

**Comment 2: What were the inclusion/exclusion criteria?**

**Response:**
The inclusion and exclusion criteria have been clarified in the manuscript. Eligible participants were women with singleton pregnancies at 26–29 weeks’ gestation, at least six years of education, a history of up to two prior vaginal births without prior cesarean section, and willingness for vaginal delivery at participating hospitals. Exclusion criteria included medical or obstetric high-risk conditions, current pregnancy complications, major fetal abnormalities, recent major stressful events, lack of smartphone or internet access, or participation in another clinical trial. (page 7, lines 16-21; page 8, lines 1-6)

**Comment 3: While the number of prenatal cares according to Iranian guideline is 8, why authors considered 9 antenatal cares?**

**Response:** We thank the reviewer for this valuable comment. The nine antenatal sessions included in the MSCOC intervention were supplementary continuity-of-care support sessions delivered by midwifery students and were separate from the routine antenatal visits recommended in the Iranian national guideline. The number of sessions was determined according to the educational structure and continuity objectives of the intervention to facilitate relationship-building and provide ongoing support throughout late pregnancy. Women in both study groups continued to receive routine antenatal care according to national guidelines. This clarification has been added to the Methods section (page 10, lines 10-20; page 11, lines 8-18, page 12, lines 11-14).

**Comment 4: What do you mean about phone/video sessions?**

**Response:** We have clarified the structure of the continuity-of-care intervention in the Methods section. According to the protocol, the continuity of care provided by students included about nine antenatal care episodes (two in-person and several scheduled phone/video sessions), birth attendance, and four postnatal follow-ups up to six weeks postpartum. (Page 10, lines 10–12).

Phone/video sessions were conducted through telephone calls or online communication platforms according to women’s preference and accessibility and were intended to provide counselling, education, emotional support, follow-up of maternal concerns, and continuity of communication between scheduled face-to-face visits. These sessions were considered part of the continuity-of-care approach to maintain ongoing contact and support throughout pregnancy and the postpartum period. We have clarified this issue in the revised manuscript (page 11, lines 1–3).

**Comment 5: In what gestational age did you start prenatal care?**

**Response:** The continuity-of-care intervention was initiated at 26–29 weeks of gestation, immediately after recruitment and baseline assessment. We have clarified this information in the Methods section (page 10, lines 7-9 and 12-13).

**Comment 6: This is not clear who conducted the prenatal cares? Students or health care providers?**

**Response:** Routine antenatal care was provided by licensed healthcare providers (midwives or obstetricians) according to national guidelines in both study groups. In addition, women in the intervention group received supplementary continuity-of-care support from trained final-year midwifery students as part of the MSCOC intervention. The students did not replace healthcare providers or independently deliver routine maternity care; rather, they provided supportive continuity-of-care activities under supervision. This clarification has been added to the Methods section (page 12, lines 11-21).

**Comment 7: This is not clear that what exactly students involved in? continuous prenatal care, or educational classes for women?**

**Response:** We thank the reviewer for this important clarification. The midwifery students were involved in providing continuous antenatal, intrapartum, and postnatal care as part of the MSCOC intervention, not limited to educational classes. Specifically, according to the protocol, during pregnancy, the students delivered approximately nine structured antenatal contacts (including two in-person assessments and multiple phone/video follow-ups), which included education (e.g., childbirth preparation, breathing and relaxation techniques, and breastfeeding counseling) with individualized continuity care, psychosocial support, and risk screening. In addition, they provided continuous support during labor and delivery and conducted four postnatal follow-ups up to six weeks postpartum. Thus, the intervention was a comprehensive continuity-of-care model rather than a standalone educational program. This clarification has been strengthened in the revised Methods section. (page 10, lines 10-20; page 11; page 12, lines 1-10). Adherence to the protocol was reported in the Results section (page 18, lines 4-7; page 19, lines 1-2).

**Comment 8: Who delivered women?**

**Response:** We clarified that deliveries were conducted by hospital midwives or obstetric staff according to hospital routine practice. Midwifery students attended labour only as support persons and had no role in clinical decision-making or delivery management. (page 12, lines 4-5)

**Comment 9: For each questionnaire/scale that you used, please write if the Iranian version was validated (you only reported reliability)?**

**Response:** We used validated Iranian versions of all questionnaires/scales in this study. Specifically, validation details for the SMMS, SCIB, and BSES instruments in Iranian populations are provided in the Methods section (page 14, lines 15–18; page 15, lines 1-5, 9-13, 15-16, 19-20).

**Comment 10: How could students and their supervisors attend 27 public health centers to recruit women?**

**Response:** The recruitment process did not require simultaneous attendance at all 27 public health centres. Instead, recruitment was conducted in a rotational and scheduled manner across the centres affiliated with the Tabriz public health system from November 2022 to February 2023 (page 16, line 17).

To recruit participants, the PI identified eligible women in advance through the SIB system and coordinated with the centres. On each recruitment day, visits were planned only to 2–3 geographically close health centres, depending on the distribution of eligible participants. Therefore, the research team (PI and 1–3 (usually two) trained midwifery students) physically presented only at the selected centres scheduled for that specific day, not all centres. We have now clarified this procedure in the revised manuscript (page 8, lines 18-21)

**Comment 11: What do you mean about modified intention-to-treat approach? Is it per protocol approach?**

**Response:** We thank the reviewer for this important methodological clarification. We would like to emphasize that the analysis was not a per-protocol approach. A modified intention-to-treat (mITT) framework was used, with participants analysed according to their original random allocation group, regardless of intervention adherence. Outcome-specific exclusions were applied only when the outcome was inherently inapplicable. Specifically, women who did not experience labour pain were excluded from analyses of instruments requiring labour experience (CEQ-2, SCIB, and EMC-LB). Likewise, women without a vaginal birth were excluded from analyses of admission-to-birth duration.

All other outcomes were analysed in the full randomized sample. This approach was adopted to preserve randomization integrity while ensuring the clinical relevance of outcome measurement. It has been clarified in the revised manuscript to prevent misinterpretation. (page 16, lines 8-10)

**Comment 12: Authors stated that deliveries took place in teaching, social security, or private hospitals. How students allowed to attend deliveries in the private hospitals?**

**Response:** We would like to clarify that student attendance during labour in private and semi-private hospitals was permitted after formal institutional approval and coordination with hospital administration (page 8, lines 1-6). Importantly, the same MSCOC labour-support intervention was implemented across all hospital types (teaching, Social Security, and private/semi-private) under an identical protocol. In all settings, midwifery students provided only continuity-of-care support during labour, including continuous bedside presence, emotional encouragement, breathing and relaxation guidance, and assistance with positioning. They were not involved in any clinical decision-making or delivery procedures.

All deliveries were conducted exclusively by licensed midwives and obstetric staff according to routine hospital practice. Students worked under the supervision of the principal investigator and/or the responsible registered midwife in each setting to ensure safety and adherence to the protocol. Allocation was stratified by hospital type to control for potential confounding effects of care setting differences. (page 11, lines 19-21; page 12, lines 1-5)

**Results**

**Comment 1: Authors stated that students only attended in 47% of deliveries, then few lines later they stated that except for one case students attended in all deliveries.**

**Response:** We thank the reviewer for this important clarification. We intended to indicate that the same primary student midwife who provided antenatal and postpartum continuity-of-care support also attended the woman during labour and childbirth in nearly all cases. The exception was one case in which a student who was not the primary care provider during pregnancy attended the delivery. This has been clarified in the revised manuscript to avoid misinterpretation. (page 19, lines 1-2)

**Comment 2: The title of table 3 should be during pregnancy and postpartum period.**

**Response:** We thank the reviewer for this suggestion. The experiences of maternity care during pregnancy were assessed at three time points: pre-intervention, at 35–36 weeks’ gestation, and at 40–50 days postpartum. Therefore, we think the current table title accurately reflects the study design and outcome measurement across pregnancy and the postpartum period. No change has been made to the table title.
