# Supplementary material for: Effect of midwifery students’ continuity of care program on women’s experiences of maternity care: A randomised controlled trial
Source: PLoS One. 2026 Jul 24;21(7):e0353118. doi: 10.1371/journal.pone.0353118 (PMC13399285; doi:10.1371/journal.pone.0353118)
Supplement: Peer review history

# Author comment: Author response to Decision Letter 0

**DOI:** 10.1371/journal.pone.0353118.r001

30 Sep 2025

Attachment Submitted filename: Response to the queries.docx

# Peer review: Decision Letter 0

**DOI:** 10.1371/journal.pone.0353118.r002

**Authors:** Forough Mortazavi

16 Nov 2025

PONE-D-25-51531

Effect of midwifery students’ continuity of care program on women’s experiences of maternity care: a randomised controlled trial

PLOS ONE

Dear Dr. Mohammad-Alizadeh-Charandabi,

Thank you for submitting your manuscript to PLOS ONE. After careful consideration, we feel that it has merit but does not fully meet PLOS ONE’s publication criteria as it currently stands. Therefore, we invite you to submit a revised version of the manuscript that addresses the points raised during the review process.<pre>Overall, the reviewers support the study’s relevance but emphasize the need for methodological clarity, statistical rigor, consistent reporting, and contextual depth—particularly regarding timing, sample handling, outcome analysis, and the Iranian maternity care context.</pre>Please submit your revised manuscript by Dec 31 2025 11:59PM. If you will need more time than this to complete your revisions, please reply to this message or contact the journal office at plosone@plos.org. When you're ready to submit your revision, log on to https://www.editorialmanager.com/pone/ and select the 'Submissions Needing Revision' folder to locate your manuscript file.

Please include the following items when submitting your revised manuscript:

- A rebuttal letter that responds to each point raised by the academic editor and reviewer(s). You should upload this letter as a separate file labeled 'Response to Reviewers'.
- A marked-up copy of your manuscript that highlights changes made to the original version. You should upload this as a separate file labeled 'Revised Manuscript with Track Changes'.
- An unmarked version of your revised paper without tracked changes. You should upload this as a separate file labeled 'Manuscript'.

-->If you would like to make changes to your financial disclosure, please include your updated statement in your cover letter. Guidelines for resubmitting your figure files are available below the reviewer comments at the end of this letter.

If applicable, we recommend that you deposit your laboratory protocols in protocols.io to enhance the reproducibility of your results. Protocols.io assigns your protocol its own identifier (DOI) so that it can be cited independently in the future. For instructions see: https://journals.plos.org/plosone/s/submission-guidelines#loc-laboratory-protocols. Additionally, PLOS ONE offers an option for publishing peer-reviewed Lab Protocol articles, which describe protocols hosted on protocols.io. Read more information on sharing protocols at https://plos.org/protocols?utm_medium=editorial-email&utm_source=authorletters&utm_campaign=protocols.

We look forward to receiving your revised manuscript.

Kind regards,

Forough Mortazavi

Academic Editor

PLOS ONE

Journal Requirements:

When submitting your revision, we need you to address these additional requirements.

1. Please ensure that your manuscript meets PLOS ONE's style requirements, including those for file naming. The PLOS ONE style templates can be found at

https://journals.plos.org/plosone/s/file?id=wjVg/PLOSOne_formatting_sample_main_body.pdf and

https://journals.plos.org/plosone/s/file?id=ba62/PLOSOne_formatting_sample_title_authors_affiliations.pdf

2. We note that the original protocol that you have uploaded as a Supporting Information file contains an institutional logo. As this logo is likely copyrighted, we ask that you please remove it from this file and upload an updated version upon resubmission.

3. Thank you for stating the following in the Acknowledgments Section of your manuscript:

“The authors express their gratitude to the Vice-Chancellor for Research of Tabriz University of Medical Sciences for financial support. They would also like to sincerely thank the midwifery students who participated in this trial as care providers and the women who took part in the study. Furthermore, they acknowledge the valuable assistance offered by the Clinical Research Development Unit of Taleghani Hospital, Tabriz University of Medical Sciences, Tabriz, Iran.”

We note that you have provided funding information that is not currently declared in your Funding Statement. However, funding information should not appear in the Acknowledgments section or other areas of your manuscript. We will only publish funding information present in the Funding Statement section of the online submission form.

Please remove any funding-related text from the manuscript and let us know how you would like to update your Funding Statement. Currently, your Funding Statement reads as follows:

“This research was funded by the Vice-Chancellor for Research at Tabriz University of Medical Sciences in Tabriz, Iran (Scientific Grant No. 69665, E.J., M.M., S.A., L.D., S.M.A.C.). The funders were not involved in the study design, participant recruitment, data collection and analysis, data interpretation, manuscript preparation, or the decision to submit the article for publication.”

Please include your amended statements within your cover letter; we will change the online submission form on your behalf.

4. In the online submission form, you indicated that “The corresponding author will provide de-identified participant datasets for research purposes to qualified researchers affiliated with academic institutions and other entities upon reasonable request, immediately after the publication of the findings.”

All PLOS journals now require all data underlying the findings described in their manuscript to be freely available to other researchers, either 1. In a public repository, 2. Within the manuscript itself, or 3. Uploaded as supplementary information.

This policy applies to all data except where public deposition would breach compliance with the protocol approved by your research ethics board. If your data cannot be made publicly available for ethical or legal reasons (e.g., public availability would compromise patient privacy), please explain your reasons on resubmission and your exemption request will be escalated for approval.

5. Your ethics statement should only appear in the Methods section of your manuscript. If your ethics statement is written in any section besides the Methods, please move it to the Methods section and delete it from any other section. Please ensure that your ethics statement is included in your manuscript, as the ethics statement entered into the online submission form will not be published alongside your manuscript.

6. Please include a caption for figure 1.

7. Please ensure that you refer to Figure 1 in your text as, if accepted, production will need this reference to link the reader to the figure.

8. Please include captions for your Supporting Information files at the end of your manuscript, and update any in-text citations to match accordingly. Please see our Supporting Information guidelines for more information: http://journals.plos.org/plosone/s/supporting-information.

9. If the reviewer comments include a recommendation to cite specific previously published works, please review and evaluate these publications to determine whether they are relevant and should be cited. There is no requirement to cite these works unless the editor has indicated otherwise.

10. Please review your reference list to ensure that it is complete and correct. If you have cited papers that have been retracted, please include the rationale for doing so in the manuscript text, or remove these references and replace them with relevant current references. Any changes to the reference list should be mentioned in the rebuttal letter that accompanies your revised manuscript. If you need to cite a retracted article, indicate the article’s retracted status in the References list and also include a citation and full reference for the retraction notice.

**Additional Editor Comments:**

Dear Authors,

Congratulations on your hard work on the midwifery students’ continuity of care study. My comments are as follows:

- Information about the care received by the control group is essential.

- Were mothers in the control group free to attend childbirth preparation classes?

- Did mothers in the intervention group have the option of being accompanied during labor by a midwife or the delivering physician?

- The reasons for elective cesarean section should be compared between the two groups.

- Why weren’t women who requested an elective cesarean section excluded from the study during recruitment?

[Note: HTML markup is below. Please do not edit.]

Reviewers' comments:

Reviewer's Responses to Questions

**Comments to the Author**

1. Is the manuscript technically sound, and do the data support the conclusions?

The manuscript must describe a technically sound piece of scientific research with data that supports the conclusions. Experiments must have been conducted rigorously, with appropriate controls, replication, and sample sizes. The conclusions must be drawn appropriately based on the data presented.

Reviewer #1: No

Reviewer #2: Yes

Reviewer #3: Yes

**********

2. Has the statistical analysis been performed appropriately and rigorously?

Reviewer #1: I Don't Know

Reviewer #2: No

Reviewer #3: I Don't Know

**********

3. Have the authors made all data underlying the findings in their manuscript fully available?

The PLOS Data policy requires authors to make all data underlying the findings described in their manuscript fully available without restriction, with rare exception (please refer to the Data Availability Statement in the manuscript PDF file). The data should be provided as part of the manuscript or its supporting information, or deposited to a public repository. For example, in addition to summary statistics, the data points behind means, medians and variance measures should be available. If there are restrictions on publicly sharing data—e.g. participant privacy or use of data from a third party—those must be specified.

Reviewer #1: No

Reviewer #2: No

Reviewer #3: Yes

**********

4. Is the manuscript presented in an intelligible fashion and written in standard English?

PLOS ONE does not copyedit accepted manuscripts, so the language in submitted articles must be clear, correct, and unambiguous. Any typographical or grammatical errors should be corrected at revision, so please note any specific errors here.

Reviewer #1: Yes

Reviewer #2: Yes

Reviewer #3: Yes

**********

5. Review Comments to the Author

Please use the space provided to explain your answers to the questions above. You may also include additional comments for the author, including concerns about dual publication, research ethics, or publication ethics. (Please upload your review as an attachment if it exceeds 20,000 characters)

Reviewer #1: Given that assessing outcomes 40-50 days after delivery can affect the accuracy of individuals' responses and the effectiveness of interventions, the reason for choosing this time and its justification are important.

Didn't the difference in the students who served as instructors and interventionists affect the results?

In cases where the trainer was not present at the time of delivery or was notified later, the sample had to be excluded from the study.

Reviewer #2: Clarify the sample size was determined based on a t-test.

Table 1: Add parity. Present Table 1 for the randomized set (Intervention n=45; Control n=48), as comparisons of maternity-care experience scores were conducted in the randomized set.

Given so many outcomes, adjust p-values for multiplicity.

Clearly justify use of last observation carried forward, stating its assumption of no change after the last measurement.

Reviewer #3: p.14, Lines 13–16

Breech presentation is typically considered an indication for elective cesarean section. Shouldn’t cases involving breech presentation be categorized accordingly under elective cesarean sections?

p.14, Lines 20–21

The sentence states: “Between-group comparisons of women who experienced labor pain showed that the intervention and control groups were similar in the baseline characteristics (Table 1).”

However, on p.14, line 13, it is noted that 8 women in the intervention group and 13 women in the control group did not experience labor pain and underwent cesarean sections. This raises the question: does Table 1 include women who did not experience labor pain?

Is it correct to interpret that ultimately, 8 out of 37 women in the intervention group and 13 out of 35 women in the control group underwent cesarean sections?

p.15, Line 13

The manuscript states: “Midwifery students in Iran have limited opportunities to gain practical experience in midwifery-centered care during their four-year bachelor course.”

Is it common in Iran for individuals to enter a four-year midwifery program directly after graduating from high school? In many countries, midwifery education requires prior clinical experience as a registered nurse. Were the midwifery students in this study individuals with no prior hospital experience? It would be helpful to include a brief description of the students’ educational and clinical backgrounds.

p.20, Line 13

In addition to reporting the average time from admission to delivery for both groups, including the shortest and longest durations would enrich the analysis and provide a more comprehensive understanding of the variability in labor experiences.

p.23, Line 10

The font size of the word “essential” appears smaller than the surrounding text. Please check for formatting inconsistencies.

p.25, Line 2

Iran’s cesarean section rate of 52% is notably high. It seems likely that factors beyond medical necessity—such as maternal preference—may contribute to this rate. Would the authors consider discussing potential sociocultural or systemic influences on cesarean section rates in Iran?

**********

6. PLOS authors have the option to publish the peer review history of their article (what does this mean?). If published, this will include your full peer review and any attached files.

If you choose “no”, your identity will remain anonymous but your review may still be made public.

**Do you want your identity to be public for this peer review?**  For information about this choice, including consent withdrawal, please see our Privacy Policy.

Reviewer #1: No

Reviewer #2: No

Reviewer #3: No

**********

[NOTE: If reviewer comments were submitted as an attachment file, they will be attached to this email and accessible via the submission site. Please log into your account, locate the manuscript record, and check for the action link "View Attachments". If this link does not appear, there are no attachment files.]

To ensure your figures meet our technical requirements, please review our figure guidelines: https://journals.plos.org/plosone/s/figures

You may also use PLOS’s free figure tool, NAAS, to help you prepare publication quality figures: https://journals.plos.org/plosone/s/figures#loc-tools-for-figure-preparation.

NAAS will assess whether your figures meet our technical requirements by comparing each figure against our figure specifications.

# Author comment: Author response to Decision Letter 1

**DOI:** 10.1371/journal.pone.0353118.r003

7 Dec 2025

Dear Scientific Editor, PLOS One

We greatly appreciate your and the reviewers’ insightful critiques of our manuscript (Manuscript PONE-D-25-51531) titled "Effect of midwifery students’ continuity of care program on women’s experiences of maternity care: a randomised controlled trial" and your constructive comments and suggestions. We have diligently addressed all of your suggestions and believe that the manuscript has significantly improved as a result. We hope that you will now consider it suitable for publication in the Journal. Below, you will find our detailed point-by-point responses to the comments. We also made all necessary changes in the manuscript as you suggested.

Yours sincerely,

Sakineh Mohammad-Alizadeh-Charandabi

Response to the Academic Editor

Comment 1: A rebuttal letter that responds to each point raised by the academic editor and reviewer(s). You should upload this letter as a separate file labeled 'Response to Reviewers'.

Response: We thank the academic editor and reviewers for their time and valuable feedback on our manuscript. A detailed rebuttal letter titled “Response to Reviewers” has been prepared and uploaded as a separate file.

Comment 2: A marked-up copy of your manuscript that highlights changes made to the original version. You should upload this as a separate file labeled 'Revised Manuscript with Track Changes'.

Response: We have uploaded a marked-up copy of the manuscript with all changes highlighted. The file is labeled “Revised Manuscript with Track Changes” and clearly indicates all modifications made in response to the comments.

Comment 3: An unmarked version of your revised paper without tracked changes. You should upload this as a separate file labeled 'Manuscript'.

Response: An unmarked version of the revised manuscript, without tracked changes, has been uploaded as a separate file labeled “Manuscript”.

Comment 4: If you would like to make changes to your financial disclosure, please include your updated statement in your cover letter.

Response: We included our updated financial disclosure statement in the cover letter.

Comment 5: Guidelines for resubmitting your figure files are available below the reviewer comments at the end of this letter.

Response: We used the guidelines for resubmitting our figure file.

Comment 6: If applicable, we recommend that you deposit your laboratory protocols in protocols.io to enhance the reproducibility of your results.

Response: This manuscript has been extracted from an extensive research project entitled “Implementation and evaluation of continuous care model by midwifery students during pregnancy, childbirth and postpartum: a mixed-methods design with Embedded Experimental Model”. The Persian version of the study protocol (including the English abstract) was registered on the University website before acceptance and receipt of an ethical code for the research conduct. The Persian and English versions of the protocol have been provided in the supplementary materials. (Page 29, line 8)

Response to Journal Requirements:

Comment 1: Please ensure that your manuscript meets PLOS ONE's style requirements, including those for file naming.

Response: We have reviewed the PLOS ONE style requirements and ensured that the manuscript and accompanying files adhere to the specified guidelines, including proper file-naming conventions. All files have been renamed accordingly to the journal's requirements.

Comment 2: We note that the original protocol that you have uploaded as a Supporting Information file contains an institutional logo. As this logo is likely copyrighted, we ask that you please remove it from this file and upload an updated version upon resubmission.

Response: We removed the institutional logo from the original protocol in the Supporting Information file, as requested. An updated version of the protocol without the logo has been uploaded upon resubmission. (Page 29, line 8)

Comment 3: Thank you for stating the following in the Acknowledgments Section of your manuscript: “The authors express their gratitude to the Vice-Chancellor for Research of Tabriz University of Medical Sciences for financial support. They would also like to sincerely thank the midwifery students who participated in this trial as care providers and the women who took part in the study. Furthermore, they acknowledge the valuable assistance offered by the Clinical Research Development Unit of Taleghani Hospital, Tabriz University of Medical Sciences, Tabriz, Iran.”

We note that you have provided funding information that is not currently declared in your Funding Statement. However, funding information should not appear in the Acknowledgments section or other areas of your manuscript. We will only publish funding information present in the Funding Statement section of the online submission form. Please remove any funding-related text from the manuscript and let us know how you would like to update your Funding Statement. Currently, your Funding Statement reads as follows: “This research was funded by the Vice-Chancellor for Research at Tabriz University of Medical Sciences in Tabriz, Iran (Scientific Grant No. 69665, E.J., M.M., S.A., L.D., S.M.A.C.). The funders were not involved in the study design, participant recruitment, data collection and analysis, data interpretation, manuscript preparation, or the decision to submit the article for publication.” Please include your amended statements within your cover letter; we will change the online submission form on your behalf.

Response: In compliance with your suggestion, we have removed the funding-related text from the Acknowledgments section of the manuscript. The revised Acknowledgments now read: “The authors sincerely thank the midwifery students who served as care providers in this trial, as well as the women who participated in the study. Furthermore, they acknowledge the valuable assistance offered by the Clinical Research Development Unit of Taleghani Hospital, Tabriz University of Medical Sciences, Tabriz, Iran.” (page 29, line 19)

Regarding the Funding Statement, we would like to update it as follows: “This research was funded by the Vice-Chancellor for Research at Tabriz University of Medical Sciences in Tabriz, Iran (Scientific Grant No. 69665, S.M.A.C.). The funder has no role in the study design, participant recruitment, data collection and analysis, data interpretation, manuscript preparation, or the decision to submit the paper for publication.” We kindly ask that you update the online submission form with this amended statement.

Comment 4: In the online submission form, you indicated that “The corresponding author will provide de-identified participant datasets for research purposes to qualified researchers affiliated with academic institutions and other entities upon reasonable request, immediately after the publication of the findings.”

All PLOS journals now require all data underlying the findings described in their manuscript to be freely available to other researchers, either 1. In a public repository, 2. Within the manuscript itself, or 3. Uploaded as supplementary information. This policy applies to all data except where public deposition would breach compliance with the protocol approved by your research ethics board. If your data cannot be made publicly available for ethical or legal reasons (e.g., public availability would compromise patient privacy), please explain your reasons on resubmission and your exemption request will be escalated for approval.

Response: We prepared all data underlying the findings described in this manuscript (including variable and value definitions) as an Excel file and uploaded it as an S1 Appendix. (page 29, line 9)

Comment 5: Your ethics statement should only appear in the Methods section of your manuscript. If your ethics statement is written in any section besides the Methods, please move it to the Methods section and delete it from any other section. Please ensure that your ethics statement is included in your manuscript, as the ethics statement entered into the online submission form will not be published alongside your manuscript.

Response: We moved the ethics statement to the Methods section (page 7, lines 5-6; page 8, lines 10-11) and removed it from other sections as requested.

Comment 6: Please include a caption for figure.

Response: We have added the following caption to the Figure:

"Figure: Flow diagram of the trial

MSCOC: Midwifery Student Continuity of Care

* Individuals who experienced labour pain.

**Individuals who had a normal birth." (page 15, lines 18-21)

Comment 7: Please ensure that you refer to Figure 1 in your text as, if accepted, production will need this reference to link the reader to the figure.

Response: We have referred to Figure in the text on page 15, line 17. This ensures proper linking of the figure within the manuscript.

Comment 8: Please include captions for your Supporting Information files at the end of your manuscript, and update any in-text citations to match accordingly. Please see our Supporting Information guidelines for more information: http://journals.plos.org/plosone/s/supporting-information.

Response: We added captions for all Supporting Information files at the end of the manuscript (page 29, line 8).

Comment 9: If the reviewer comments include a recommendation to cite specific previously published works, please review and evaluate these publications to determine whether they are relevant and should be cited. There is no requirement to cite these works unless the editor has indicated otherwise.

Response: The reviewers have not recommended citing any specific previously published works.

Comment 10: Please review your reference list to ensure that it is complete and correct. If you have cited papers that have been retracted, please include the rationale for doing so in the manuscript text, or remove these references and replace them with relevant current references. Any changes to the reference list should be mentioned in the rebuttal letter that accompanies your revised manuscript. If you need to cite a retracted article, indicate the article’s retracted status in the References list and also include a citation and full reference for the retraction notice.

Response: We have carefully rechecked the reference list, and none of the cited papers have been retracted. In response to the reviewers’ comments, we have added several new references, all of which are marked with track changes and highlighted in the manuscript.

Response to Additional Editor Comments:

Comment 1: Information about the care received by the control group is essential.

Response: Both groups received routine care throughout pregnancy, labor, and the postpartum period. We have added details about this care and the childbirth preparation classes at the end of the Intervention section (pages 11, lines 9-22; page 12, lines 1-10).

Comment 2: Were mothers in the control group free to attend childbirth preparation classes?

Response: Yes, both groups were free to participate in childbirth preparation classes. We have added this information to the manuscript (page 11, lines 9-10). However, most pregnant women in Iran do not attend the classes.

Among the women who experienced labor pain, 2 women (5.4%) in the MSCOC group attended the classes, compared to 5 women (14.3%) in the control group. The frequency of participation was included in Table 1.

Comment 3: Did mothers in the intervention group have the option of being accompanied during labor by a midwife or the delivering physician?

Response: In Iran, physicians typically do not accompany women during labor. The accompanying midwife, usually unfamiliar with the woman beforehand, attends the labor at the mother’s request as a private midwife. She provides ongoing emotional and psychological support but does not participate in clinical decision-making or medical care.

Both groups had the option to be accompanied during labor by a private midwife or a female family member, depending on hospital policies. This information has been added to the manuscript (page 11, lines 10-12).

Among women who experienced labor pain, 2 (5.4%) in the MSCOC group were accompanied by a midwife, and 9 (24.3%) by a family member. In the control group, 4 (11.4%) were accompanied by a midwife, and 8 (23%) by a family member. The frequencies were also included in Table 1.

Comment 4: The reasons for elective cesarean section should be compared between the two groups.

Response: Among women who chose elective cesarean sections, two in the MSCOC group did so with their obstetrician's encouragement, while one chose it at her own request. In the control group, four women underwent the procedure with the obstetrician's encouragement, one was motivated by her mother-in-law, and six at their own request. Also, one woman in the intervention group and two women in the control group had cesarean sections due to breech presentation. This information has been added to the manuscript. (page 22, lines 14-17).

Comment 5: Why weren’t women who requested an elective cesarean section excluded from the study during recruitment?

Response: As stated in the Participants section of the manuscript, one of the eligibility criteria was "willingness to have a vaginal birth at one of nine city-level main hospitals" (page 7, line 15). Therefore, women who requested an elective cesarean section were excluded during recruitment. However, since our study followed an intention-to-treat design, women who later changed their preference and requested an elective cesarean section during the study were not excluded and followed-up until 40-50 days after delivery.

Response to Reviewer #1:

Comment 1: Given that assessing outcomes 40-50 days after delivery can affect the accuracy of individuals' responses and the effectiveness of interventions, the reason for choosing this time and its justification are important.

Response: Midwife-led continuity of care begins during pregnancy and extends through labor, often into the early postpartum period [1, 2]. Accordingly, our intervention started at 26–29 weeks of gestation and continued until 20–30 days postpartum. Therefore, outcomes which may also be influenced by postnatal care, such as the experience of maternity care [3] and breastfeeding self-efficacy [4], were assessed 20 to 30 days after the intervention ended.

Childbirth experience was our primary outcome. The Childbirth Experience Questionnaire (CEQ-2) is typically administered between 4 and 8 weeks postpartum [5-7]. Research suggests that measuring the childbirth experience too soon may yield overly positive responses, influenced by the immediate happiness of having a healthy child. Allowing a period of reflection leads to a more stable and accurate representation of a woman's birth experience [7, 8].

An explanatory note regarding this timing has been added to the revised manuscript. (page 12, lines 13-14 and 18-20)

Comment 2: Didn't the difference in the students who served as instructors and interventionists affect the results?

Response: We have described the continuity of care during labor and delivery in the intervention section of the manuscript (page 10, lines 18-22). Except for one case, the same student midwife who conducted the educational sessions during pregnancy and the postpartum period also attended the woman during labor and childbirth as a supporter. In none of the cases, the student had no role in decision-making for medical interventions or in childbirth (page 17, lines 7-9)

Comment 3: In cases where the trainer was not present at the time of delivery or was notified later, the sample had to be excluded from the study.

Response: We followed a pre-planned modified intention-to-treat approach, following all recruited participants until the end of the trial and assessing all outcomes that could be assessed, without considering their protocol adherence. All participants were included in the analysis, in the groups to which they were originally assigned, excluding only those whose outcomes could not be assessed (page 15, lines 3-4). However, detail of protocol adherence was described in the Results section of the manuscript (page 17, lines 4-9).

Response to Reviewer #2

Comment 1: Clari

Attachment Submitted filename: Response to Reviewers.docx

# Peer review: Decision Letter 1

**DOI:** 10.1371/journal.pone.0353118.r004

**Authors:** Reem Saeed Alghamdi

24 Apr 2026

PONE-D-25-51531R1

Effect of midwifery students’ continuity of care program on women’s experiences of maternity care: a randomised controlled trial

PLOS One

Dear Dr. Mohammad-Alizadeh-Charandabi,

Thank you for submitting your manuscript to PLOS ONE. After careful consideration, we feel that it has merit but does not fully meet PLOS ONE’s publication criteria as it currently stands. Therefore, we invite you to submit a revised version of the manuscript that addresses the points raised during the review process.

Please submit your revised manuscript by Jun 08 2026 11:59PM. If you will need more time than this to complete your revisions, please reply to this message or contact the journal office at plosone@plos.org. When you're ready to submit your revision, log on to https://www.editorialmanager.com/pone/ and select the 'Submissions Needing Revision' folder to locate your manuscript file.

Please include the following items when submitting your revised manuscript:

- A letter that responds to each point raised by the academic editor and reviewer(s). You should upload this letter as a separate file labeled 'Response to Reviewers'.
- A marked-up copy of your manuscript that highlights changes made to the original version. You should upload this as a separate file labeled 'Revised Manuscript with Track Changes'.
- An unmarked version of your revised paper without tracked changes. You should upload this as a separate file labeled 'Manuscript'.

If you would like to make changes to your financial disclosure, please include your updated statement in your cover letter. Guidelines for resubmitting your figure files are available below the reviewer comments at the end of this letter.

If applicable, we recommend that you deposit your laboratory protocols in protocols.io to enhance the reproducibility of your results. Protocols.io assigns your protocol its own identifier (DOI) so that it can be cited independently in the future. For instructions see: https://journals.plos.org/plosone/s/submission-guidelines#loc-laboratory-protocols. Additionally, PLOS ONE offers an option for publishing peer-reviewed Lab Protocol articles, which describe protocols hosted on protocols.io. Read more information on sharing protocols at https://plos.org/protocols?utm_medium=editorial-email&utm_source=authorletters&utm_campaign=protocols.

As the corresponding author, your ORCID iD is verified in the submission system and will appear in the published article. PLOS supports the use of ORCID, and we encourage all coauthors to register for an ORCID iD and use it as well. Please encourage your coauthors to verify their ORCID iD within the submission system before final acceptance, as unverified ORCID iDs will not appear in the published article. *Only*  the individual author can complete the verification step; PLOS staff *cannot*  verify ORCID iDs on behalf of authors.

We look forward to receiving your revised manuscript.

Kind regards,

Reem Saeed Alghamdi, PhD., Consultant Midwife

Guest Editor

PLOS One

Journal Requirements:

If the reviewer comments include a recommendation to cite specific previously published works, please review and evaluate these publications to determine whether they are relevant and should be cited. There is no requirement to cite these works unless the editor has indicated otherwise.

Please review your reference list to ensure that it is complete and correct. If you have cited papers that have been retracted, please include the rationale for doing so in the manuscript text, or remove these references and replace them with relevant current references. Any changes to the reference list should be mentioned in the rebuttal letter that accompanies your revised manuscript. If you need to cite a retracted article, indicate the article’s retracted status in the References list and also include a citation and full reference for the retraction notice.

[Note: HTML markup is below. Please do not edit.]

Reviewers' comments:

Reviewer's Responses to Questions

**Comments to the Author**

1. If the authors have adequately addressed your comments raised in a previous round of review and you feel that this manuscript is now acceptable for publication, you may indicate that here to bypass the “Comments to the Author” section, enter your conflict of interest statement in the “Confidential to Editor” section, and submit your "Accept" recommendation.

Reviewer #3: All comments have been addressed

Reviewer #4: All comments have been addressed

Reviewer #5: All comments have been addressed

**********

2. Is the manuscript technically sound, and do the data support the conclusions?

The manuscript must describe a technically sound piece of scientific research with data that supports the conclusions. Experiments must have been conducted rigorously, with appropriate controls, replication, and sample sizes. The conclusions must be drawn appropriately based on the data presented.

Reviewer #3: Yes

Reviewer #4: (No Response)

Reviewer #5: Yes

**********

3. Has the statistical analysis been performed appropriately and rigorously?

Reviewer #3: I Don't Know

Reviewer #4: (No Response)

Reviewer #5: Yes

**********

4. Have the authors made all data underlying the findings in their manuscript fully available?

The PLOS Data policy requires authors to make all data underlying the findings described in their manuscript fully available without restriction, with rare exception (please refer to the Data Availability Statement in the manuscript PDF file). The data should be provided as part of the manuscript or its supporting information, or deposited to a public repository. For example, in addition to summary statistics, the data points behind means, medians and variance measures should be available. If there are restrictions on publicly sharing data—e.g. participant privacy or use of data from a third party—those must be specified.

Reviewer #3: Yes

Reviewer #4: (No Response)

Reviewer #5: Yes

**********

5. Is the manuscript presented in an intelligible fashion and written in standard English?

PLOS ONE does not copyedit accepted manuscripts, so the language in submitted articles must be clear, correct, and unambiguous. Any typographical or grammatical errors should be corrected at revision, so please note any specific errors here.

Reviewer #3: Yes

Reviewer #4: (No Response)

Reviewer #5: Yes

**********

6. Review Comments to the Author

Please use the space provided to explain your answers to the questions above. You may also include additional comments for the author, including concerns about dual publication, research ethics, or publication ethics. (Please upload your review as an attachment if it exceeds 20,000 characters)

Reviewer #3: The revised version is now easier to understand.

In line with CONSORT statement, baseline characteristics should be presented descriptively without statistical testing. Therefore, I suggest removing the P value for baseline variables from Table1 to avoid potential misinterpretation.

Reviewer #4: (No Response)

Reviewer #5: Thank you as you responded the comments of other reviewers in the previous round, however, there are still some minor comments that should be addressed by authors:

Thank you for inviting me to review this manuscript. Please see my comments as follows:

Abstract

1. Why authors started recruitment of women at 26-29 weeks?

2. Why women in the intervention group also received routine care?

3. How many prenatal care women received by students?

4. Did students implement prenatal care under the supervision?

Methods

1. Please write the title of the large mixed method study.

2. What were the inclusion/exclusion criteria?

3. While the number of prenatal cares according to Iranian guideline is 8, why authors considered 9 antenatal cares?

4. What do you mean about phone/video sessions?

5. In what gestational age did you start prenatal care?

6. This is not clear who conducted the prenatal cares? Students or health care providers?

7. This is not clear that what exactly students involved in? continuous prenatal care, or educational classes for women?

8. Who delivered women?

9. For each questionnaire/scale that you used, please write if the Iranian version was validated (you only reported reliability)?

10. How could students and their supervisors attend 27 public health centers to recruit women?

11. What do you mean about modified intention-to-treat approach? Is it per protocol approach?

12. Authors stated that deliveries took place in teaching, social security, or private hospitals. How students allowed to attend deliveries in the private hospitals?

Results

1. Authors stated that students only attended in 47% of deliveries, then few lines later they stated that except for one case students attended in all deliveries.

2. The title of table 3 should be during pregnancy and postpartum period.

**********

7. PLOS authors have the option to publish the peer review history of their article (what does this mean?). If published, this will include your full peer review and any attached files.

If you choose “no”, your identity will remain anonymous but your review may still be made public.

**Do you want your identity to be public for this peer review?**  For information about this choice, including consent withdrawal, please see our Privacy Policy.

Reviewer #3: No

Reviewer #4: No

Reviewer #5: No

**********

[NOTE: If reviewer comments were submitted as an attachment file, they will be attached to this email and accessible via the submission site. Please log into your account, locate the manuscript record, and check for the action link "View Attachments". If this link does not appear, there are no attachment files.]

To ensure your figures meet our technical requirements, please review our figure guidelines: https://journals.plos.org/plosone/s/figures

You may also use PLOS’s free figure tool, NAAS, to help you prepare publication quality figures: https://journals.plos.org/plosone/s/figures#loc-tools-for-figure-preparation.

NAAS will assess whether your figures meet our technical requirements by comparing each figure against our figure specifications.

# Author comment: Author response to Decision Letter 2

**DOI:** 10.1371/journal.pone.0353118.r005

30 May 2026

Dear Dr Reem Saeed Alghamdi,

Guest Editor, PLOS One

We sincerely thank you and the reviewers for the careful evaluation of our revised manuscript (Manuscript ID: PONE-D-25-51531) entitled “Effect of midwifery students’ continuity of care program on women’s experiences of maternity care: a randomised controlled trial.”

We appreciate the opportunity to further improve the manuscript. We have addressed all comments and revised the manuscript accordingly. All changes are clearly marked in the tracked version of the manuscript, and the corresponding page and line numbers are provided for each modification.

We hope that the revised manuscript now meets the publication standards of PLOS ONE.

Yours sincerely,

Sakineh Mohammad-Alizadeh-Charandabi

Response to the Academic Editor

Comment 1: If applicable, we recommend that you deposit your laboratory protocols in protocols.io to enhance the reproducibility of your results. Protocols.io assigns your protocol its own identifier (DOI) so that it can be cited independently in the future.

Response: Thank you for this valuable suggestion. Although the study protocol was not registered in protocols.io, this manuscript was extracted from an extensive research project entitled “Implementation and evaluation of continuous care model by midwifery students during pregnancy, childbirth and postpartum: a mixed-methods design with Embedded Experimental Model”. The Persian version of the study protocol (including the English abstract) had been registered on the University website before obtaining ethical approval for conducting the study. To enhance transparency and reproducibility, both the Persian and English versions of the study protocol have been provided as supplementary materials. (Page 31, lines 1-6)

Comment 2: As the corresponding author, your ORCID iD is verified in the submission system and will appear in the published article. PLOS supports the use of ORCID, and we encourage all coauthors to register for an ORCID iD and use it as well. Please encourage your coauthors to verify their ORCID iD within the submission system before final acceptance, as unverified ORCID iDs will not appear in the published article. Only the individual author can complete the verification step; PLOS staff cannot verify ORCID iDs on behalf of authors.

Response: Thank you for this suggestion. We have added ORCID IDs of all coauthors to the revised manuscript and the submission system. We all encouraged all coauthors to verify their ORCID IDs within the submission system before final acceptance.

Response to Journal Requirements:

Comment 1: If the reviewer comments include a recommendation to cite specific previously published works, please review and evaluate these publications to determine whether they are relevant and should be cited. There is no requirement to cite these works unless the editor has indicated otherwise.

Response: The reviewers have not recommended citing any specific previously published works.

Comment 2: Please review your reference list to ensure that it is complete and correct. If you have cited papers that have been retracted, please include the rationale for doing so in the manuscript text, or remove these references and replace them with relevant current references. Any changes to the reference list should be mentioned in the rebuttal letter that accompanies your revised manuscript. If you need to cite a retracted article, indicate the article’s retracted status in the References list and also include a citation and full reference for the retraction notice.

Response: No retracted publications were included in the reference list. All references were verified for validity and currency before resubmission.

Response to Reviewer #3

Comment 1: In line with CONSORT statement, baseline characteristics should be presented descriptively without statistical testing. Therefore, I suggest removing the P value for baseline variables from Table1 to avoid potential misinterpretation.

Response: We thank the reviewer for this methodological suggestion. In accordance with CONSORT guidelines, p-values for baseline characteristics have been removed. Table 1 now presents baseline variables descriptively only to avoid misinterpretation. (Table 1, page 17)

________________________________________

Response to Reviewer #5

Abstract

Comment 1: Why authors started recruitment of women at 26–29 weeks?

Response: Continuity-of-care models typically span pregnancy, labour, and the postpartum period; however, there is no consensus on the optimal gestational age for initiating such models [1, 2]. In this study, participants were recruited at 26–29 weeks’ gestation. The intervention was started immediately after the recruitment for the intervention group to ensure adequate time to establish continuity relationships and deliver the intervention prior to labour, while maintaining follow-up through childbirth and the postpartum period. This clarification has been added to the Methods section (page 10, lines 7-9). We have also acknowledged in the Limitations section that earlier initiation of the intervention may have increased exposure to continuity of care and potentially enhanced intervention effects (page 29, lines 10-12).

1. Sandall J, Fernandez Turienzo C, Devane D, Soltani H, Gillespie P, Gates S, Jones LV, Shennan AH, Rayment-Jones H: Midwife continuity of care models versus other models of care for childbearing women. The Cochrane database of systematic reviews 2024, 4(4):Cd004667.

2. Bradford BF, Wilson AN, Portela A, McConville F, Fernandez Turienzo C, Homer CSE: Midwifery continuity of care: A scoping review of where, how, by whom and for whom? PLOS global public health 2022, 2(10):e0000935

Comment 2: Why women in the intervention group also received routine care?

Response: We thank the reviewer for this important comment. The Midwifery Student Continuity of Care (MSCOC) intervention was implemented as a supplementary model integrated into the existing maternity care system, rather than as a replacement for routine services. Accordingly, participants in the intervention group received standard antenatal, intrapartum, and postpartum care in line with national guidelines, alongside continuity-of-care support delivered by trained midwifery students. This design ensured ethical provision of routine maternity care to all participants while enabling evaluation of the added effect of the MSCOC intervention within the current healthcare context. We have clarified this in the Methods section (page 12, lines 11-21; page 13, lines 1-16).

Comment 3: How many prenatal care women received by students?

Response: According to the protocol, the continuity-of-care intervention delivered by midwifery students comprised approximately nine antenatal contacts, including two in-person sessions and multiple scheduled telephone/video consultations during pregnancy. Continuity was maintained during labour, followed by four structured postpartum follow-up sessions up to six weeks after birth. This description has been clarified in the Methods section (page 10, lines 10–20).

We also reported adherence to the intervention in the Results section, showing that 34 women (76%) in the intervention group attended at least six antenatal sessions (page 18, lines 4-5).

Comment 4: Did students implement prenatal care under the supervision?

Response: Midwifery students did not provide routine antenatal care independently and did not replace licensed healthcare providers. All standard antenatal care was delivered by licensed midwives or obstetricians according to national guidelines. The role of the midwifery students was limited to providing supplementary continuity-of-care support. The students were supervised by the principal investigator, who monitored students’ performance and provided ongoing guidance and feedback throughout the study. This clarification has been added to the Methods section (page 12, lines 11-14).

Methods

Comment 1: Please write the title of the large mixed method study.

Response:

We thank the reviewer for this helpful suggestion. We have added the title of the overarching mixed-methods project: “Implementation and evaluation of a continuous care model by midwifery students during pregnancy, childbirth, and postpartum: a mixed-methods study with an embedded experimental design.” We have also clarified that the present manuscript reports the quantitative experimental component of this larger study. This information has been included in the Methods section (page 7, lines 2–9).

Comment 2: What were the inclusion/exclusion criteria?

Response:

The inclusion and exclusion criteria have been clarified in the manuscript. Eligible participants were women with singleton pregnancies at 26–29 weeks’ gestation, at least six years of education, a history of up to two prior vaginal births without prior cesarean section, and willingness for vaginal delivery at participating hospitals. Exclusion criteria included medical or obstetric high-risk conditions, current pregnancy complications, major fetal abnormalities, recent major stressful events, lack of smartphone or internet access, or participation in another clinical trial. (page 7, lines 16-21; page 8, lines 1-6)

Comment 3: While the number of prenatal cares according to Iranian guideline is 8, why authors considered 9 antenatal cares?

Response: We thank the reviewer for this valuable comment. The nine antenatal sessions included in the MSCOC intervention were supplementary continuity-of-care support sessions delivered by midwifery students and were separate from the routine antenatal visits recommended in the Iranian national guideline. The number of sessions was determined according to the educational structure and continuity objectives of the intervention to facilitate relationship-building and provide ongoing support throughout late pregnancy. Women in both study groups continued to receive routine antenatal care according to national guidelines. This clarification has been added to the Methods section (page 10, lines 10-20; page 11, lines 8-18, page 12, lines 11-14).

Comment 4: What do you mean about phone/video sessions?

Response: We have clarified the structure of the continuity-of-care intervention in the Methods section. According to the protocol, the continuity of care provided by students included about nine antenatal care episodes (two in-person and several scheduled phone/video sessions), birth attendance, and four postnatal follow-ups up to six weeks postpartum. (Page 10, lines 10–12).

Phone/video sessions were conducted through telephone calls or online communication platforms according to women’s preference and accessibility and were intended to provide counselling, education, emotional support, follow-up of maternal concerns, and continuity of communication between scheduled face-to-face visits. These sessions were considered part of the continuity-of-care approach to maintain ongoing contact and support throughout pregnancy and the postpartum period. We have clarified this issue in the revised manuscript (page 11, lines 1–3).

Comment 5: In what gestational age did you start prenatal care?

Response: The continuity-of-care intervention was initiated at 26–29 weeks of gestation, immediately after recruitment and baseline assessment. We have clarified this information in the Methods section (page 10, lines 7-9 and 12-13).

Comment 6: This is not clear who conducted the prenatal cares? Students or health care providers?

Response: Routine antenatal care was provided by licensed healthcare providers (midwives or obstetricians) according to national guidelines in both study groups. In addition, women in the intervention group received supplementary continuity-of-care support from trained final-year midwifery students as part of the MSCOC intervention. The students did not replace healthcare providers or independently deliver routine maternity care; rather, they provided supportive continuity-of-care activities under supervision. This clarification has been added to the Methods section (page 12, lines 11-21).

Comment 7: This is not clear that what exactly students involved in? continuous prenatal care, or educational classes for women?

Response: We thank the reviewer for this important clarification. The midwifery students were involved in providing continuous antenatal, intrapartum, and postnatal care as part of the MSCOC intervention, not limited to educational classes. Specifically, according to the protocol, during pregnancy, the students delivered approximately nine structured antenatal contacts (including two in-person assessments and multiple phone/video follow-ups), which included education (e.g., childbirth preparation, breathing and relaxation techniques, and breastfeeding counseling) with individualized continuity care, psychosocial support, and risk screening. In addition, they provided continuous support during labor and delivery and conducted four postnatal follow-ups up to six weeks postpartum. Thus, the intervention was a comprehensive continuity-of-care model rather than a standalone educational program. This clarification has been strengthened in the revised Methods section. (page 10, lines 10-20; page 11; page 12, lines 1-10). Adherence to the protocol was reported in the Results section (page 18, lines 4-7; page 19, lines 1-2).

Comment 8: Who delivered women?

Response: We clarified that deliveries were conducted by hospital midwives or obstetric staff according to hospital routine practice. Midwifery students attended labour only as support persons and had no role in clinical decision-making or delivery management. (page 12, lines 4-5)

Comment 9: For each questionnaire/scale that you used, please write if the Iranian version was validated (you only reported reliability)?

Response: We used validated Iranian versions of all questionnaires/scales in this study. Specifically, validation details for the SMMS, SCIB, and BSES instruments in Iranian populations are provided in the Methods section (page 14, lines 15–18; page 15, lines 1-5, 9-13, 15-16, 19-20).

Comment 10: How could students and their supervisors attend 27 public health centers to recruit women?

Response: The recruitment process did not require simultaneous attendance at all 27 public health centres. Instead, recruitment was conducted in a rotational and scheduled manner across the centres affiliated with the Tabriz public health system from November 2022 to February 2023 (page 16, line 17).

To recruit participants, the PI identified eligible women in advance through the SIB system and coordinated with the centres. On each recruitment day, visits were planned only to 2–3 geographically close health centres, depending on the distribution of eligible participants. Therefore, the research team (PI and 1–3 (usually two) trained midwifery students) physically presented only at the selected centres scheduled for that specific day, not all centres. We have now clarified this procedure in the revised manuscript (page 8, lines 18-21)

Comment 11: What do you mean about modified intention-to-treat approach? Is it per protocol approach?

Response: We thank the reviewer for this important methodological clarification. We would like to emphasize that the analysis was not a per-protocol approach. A modified intention-to-treat (mITT) framework was used, with participants analysed according to their original random allocation group, regardless of intervention adherence. Outcome-specific exclusions were applied only when the outcome was inherently inapplicable. Specifically, women who did not experience labour pain were excluded from analyses of instruments requiring labour experience (CEQ-2, SCIB, and EMC-LB). Likewise, women without a vaginal birth were excluded from analyses of admission-to-birth duration.

All other outcomes were analysed in the full randomized sample. This approach was adopted to preserve randomization integrity while ensuring the clinical relevance of outcome measurement. It has been clarified in the revised manuscript to prevent misinterpretation. (page 16, lines 8-10)

Comment 12: Authors stated that deliveries took place in teaching, social security, or private hospitals. How students allowed to attend deliveries in the private hospitals?

Attachment Submitted filename: Response to Reviewers (2).docx

# Peer review: Decision Letter 2

**DOI:** 10.1371/journal.pone.0353118.r006

**Authors:** Reem Saeed Alghamdi

18 Jun 2026

Effect of midwifery students’ continuity of care program on women’s experiences of maternity care: a randomised controlled trial

PONE-D-25-51531R2

Dear Dr. Mohammad-Alizadeh-Charandabi,

We’re pleased to inform you that your manuscript has been judged scientifically suitable for publication and will be formally accepted for publication once it meets all outstanding technical requirements.

Within one week, you’ll receive an e-mail detailing the required amendments. When these have been addressed, you’ll receive a formal acceptance letter and your manuscript will be scheduled for publication.

An invoice will be generated when your article is formally accepted. Please note, if your institution has a publishing partnership with PLOS and your article meets the relevant criteria, all or part of your publication costs will be covered. Please make sure your user information is up-to-date by logging into Editorial Manager at Editorial Manager® and clicking the ‘Update My Information' link at the top of the page. For questions related to billing, please contact billing support.

If your institution or institutions have a press office, please notify them about your upcoming paper to help maximize its impact. If they’ll be preparing press materials, please inform our press team as soon as possible -- no later than 48 hours after receiving the formal acceptance. Your manuscript will remain under strict press embargo until 2 pm Eastern Time on the date of publication. For more information, please contact onepress@plos.org.

Kind regards,

Reem Saeed Alghamdi, PhD., Consultant Midwife

Guest Editor

PLOS One

Additional Editor Comments (optional):

Reviewers' comments:

Reviewer's Responses to Questions

**Comments to the Author**

1. If the authors have adequately addressed your comments raised in a previous round of review and you feel that this manuscript is now acceptable for publication, you may indicate that here to bypass the “Comments to the Author” section, enter your conflict of interest statement in the “Confidential to Editor” section, and submit your "Accept" recommendation.

Reviewer #2: All comments have been addressed

**********

2. Is the manuscript technically sound, and do the data support the conclusions?

The manuscript must describe a technically sound piece of scientific research with data that supports the conclusions. Experiments must have been conducted rigorously, with appropriate controls, replication, and sample sizes. The conclusions must be drawn appropriately based on the data presented.

Reviewer #2: (No Response)

**********

3. Has the statistical analysis been performed appropriately and rigorously?

Reviewer #2: (No Response)

**********

4. Have the authors made all data underlying the findings in their manuscript fully available?

The PLOS Data policy requires authors to make all data underlying the findings described in their manuscript fully available without restriction, with rare exception (please refer to the Data Availability Statement in the manuscript PDF file). The data should be provided as part of the manuscript or its supporting information, or deposited to a public repository. For example, in addition to summary statistics, the data points behind means, medians and variance measures should be available. If there are restrictions on publicly sharing data—e.g. participant privacy or use of data from a third party—those must be specified.

Reviewer #2: (No Response)

**********

5. Is the manuscript presented in an intelligible fashion and written in standard English?

PLOS ONE does not copyedit accepted manuscripts, so the language in submitted articles must be clear, correct, and unambiguous. Any typographical or grammatical errors should be corrected at revision, so please note any specific errors here.

Reviewer #2: (No Response)

**********

6. Review Comments to the Author

Please use the space provided to explain your answers to the questions above. You may also include additional comments for the author, including concerns about dual publication, research ethics, or publication ethics. (Please upload your review as an attachment if it exceeds 20,000 characters)

Reviewer #2: (No Response)

**********

7. PLOS authors have the option to publish the peer review history of their article (what does this mean?). If published, this will include your full peer review and any attached files.

If you choose “no”, your identity will remain anonymous but your review may still be made public.

**Do you want your identity to be public for this peer review?**  For information about this choice, including consent withdrawal, please see our Privacy Policy.

Reviewer #2: No

**********

# Editor report: Acceptance letter

**DOI:** 10.1371/journal.pone.0353118.r007

**Authors:** Reem Saeed Alghamdi

PONE-D-25-51531R2

PLOS One

Dear Dr. Mohammad-Alizadeh-Charandabi,

I'm pleased to inform you that your manuscript has been deemed suitable for publication in PLOS One. Congratulations! Your manuscript is now being handed over to our production team.

At this stage, our production department will prepare your paper for publication. This includes ensuring the following:

* All references, tables, and figures are properly cited

* All relevant supporting information is included in the manuscript submission,

* There are no issues that prevent the paper from being properly typeset

You will receive further instructions from the production team, including instructions on how to review your proof when it is ready. Please keep in mind that we are working through a large volume of accepted articles, so please give us a few days to review your paper and let you know the next and final steps.

Lastly, if your institution or institutions have a press office, please let them know about your upcoming paper now to help maximize its impact. If they'll be preparing press materials, please inform our press team within the next 48 hours. Your manuscript will remain under strict press embargo until 2 pm Eastern Time on the date of publication. For more information, please contact onepress@plos.org.

You will receive an invoice from PLOS for your publication fee after your manuscript has reached the completed accept phase. If you receive an email requesting payment before acceptance or for any other service, this may be a phishing scheme. Learn how to identify phishing emails and protect your accounts at https://explore.plos.org/phishing.

If we can help with anything else, please email us at customercare@plos.org.

Thank you for submitting your work to PLOS ONE and supporting open access.

Kind regards,

PLOS ONE Editorial Office Staff

on behalf of

Dr. Reem Saeed Alghamdi

Guest Editor

PLOS One
